# Stoichioproteomics reveal oxygen usage bias, key proteins and pathways in glioma

**DOI:** 10.1186/s12920-019-0571-y

**Published:** 2019-08-29

**Authors:** Yongqin Yin, Bo Li, Kejie Mou, Muhammad T. Khan, Aman C. Kaushik, Dongqing Wei, Yu-Juan Zhang

**Affiliations:** 1 Chongqing Key Laboratory of Vector Insects, Institute of Entomology and Molecular Biology, College of Life Sciences, Chongqing Normal University, Shapingba, University City, Chongqing, 401331 People’s Republic of China; 2 Department of Neurosurgery, Bishan Hospital, Bishan, Chongqing, 402760 China; 3 Shanghai Jiao Tong University, Shanghai, China; 4 Peng Cheng Laboratory, Vanke Cloud City Phase I Building 8, Xili Street, Nanshan District, Shenzhen, Guangdong 518055 China; 5 Capital University of Science & Technology, Islamabad, Pakistan

**Keywords:** Hypoxia, Elements content, Proteome, Glioma

## Abstract

**Background:**

The five-year survival rate and therapeutic effect of malignant glioma is low. Identification of key/associated proteins and pathways in glioma is necessary for developing effective diagnosis and targeted therapy of glioma. In addition, Glioma involves hypoxia-specific microenvironment, whether hypoxia restriction influences the stoichioproteomic characteristics of expressed proteins is unknown.

**Methods:**

In this study, we analyzed the most comprehensive immunohistochemical data from 12 human glioma samples and 4 normal cell types of cerebral cortex, identified differentially expressed proteins (DEPs), and researched the oxygen contents of DEPs, highly and lowly expressed proteins. Further we located key genes on human genome to determine their locations and enriched them for key functional pathways.

**Results:**

Our results showed that although no difference was detected on whole proteome, the average oxygen content of highly expressed proteins is 6.65% higher than that of lowly expressed proteins in glioma. A total of 1480 differentially expressed proteins were identified in glioma, including 226 up regulated proteins and 1254 down regulated proteins. The average oxygen content of up regulated proteins is 2.56% higher than that of down regulated proteins in glioma. The localization of differentially expressed genes on human genome showed that most genes were on chromosome 1 and least on Y. The up regulated proteins were significantly enriched in pathways including cell cycle, pathways in cancer, oocyte meiosis, DNA replication etc. Functional dissection of the up regulated proteins with high oxygen contents showed that 51.28% of the proteins were involved in cell cycle and cyclins.

**Conclusions:**

Element signature of oxygen limitation could not be detected in glioma, just as what happened in plants and microbes. Unsaved use of oxygen by the highly expressed proteins and DEPs were adapted to the fast division of glioma cells. This study can help to reveal the molecular mechanism of glioma, and provide a new approach for studies of cancer-related biomacromolecules. In addition, this study lays a foundation for application of stoichioproteomics in precision medicine.

**Electronic supplementary material:**

The online version of this article (10.1186/s12920-019-0571-y) contains supplementary material, which is available to authorized users.

## Background

Glioma is the most popular primary malignant tumor in neurosurgery [1], and its five-year survival rate is low, less than 10% [2]. As having been reported earlier [3], malignant glioma cells can extensively invade normal brain tissue, and form highly proliferative glioma cell clusters [4]. Moreover, the high infiltration of glioma is one of its features, which makes it extremely difficult to cure completely [5]. Seizure and cognitive disorder are the most commonly observed symptoms of glioma in adults [6]. Beside the lesion of glioma, these additional problems caused by glioma also haunt patients [7, 8].

**Fig. 1:**
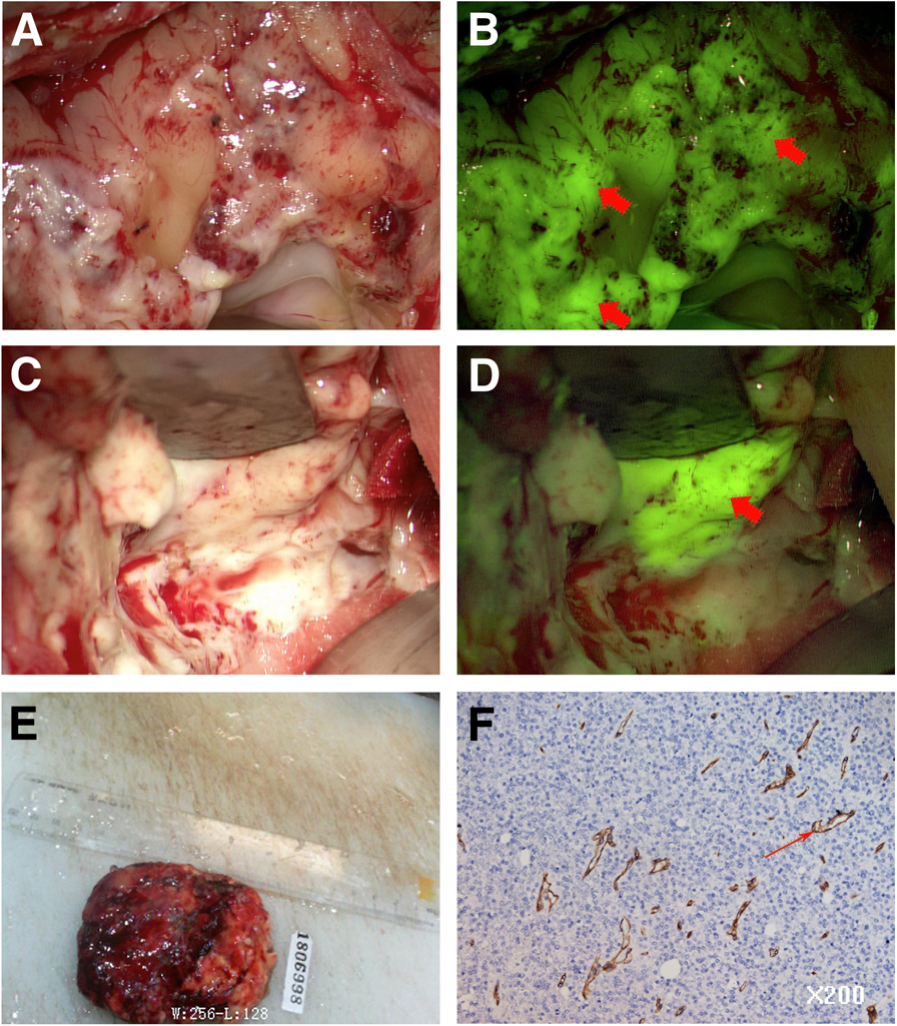
A case of recurrent glioblastoma multiforme (GBM). It is difficult to distinguish tumor from normal brain tissue under a white light field (**a**: Preoperative tumor lesion. **c**: the tumor lesions during procedures). But easy to distinguish tumor tissue from normal brain tissue under a fluorescence microscope (**b** and **d**: Strong presence of yellow fluorescence in the tumor lesions during procedures). **e**: Excised whole tumor tissue. **f** Postoperative pathological examination showed that largely decreased and discontinuous expression of claudin-5 was observed in the endothelial cells from the tumor lesions. The short red arrows refer to the tumor lesions. The long red arrow refers to the expression of claudin-5 in the endothelial cells

Traditionally, surgery is the most frequently suggested therapy approach in treating glioma [9]. But surgical treatments can sometimes cause serious postoperative complications, including cerebral vascular injury, seizures, hematomas, and so on [10]. With the advancement of medical science, more and more methods are available for glioma treatments nowadays, such as the technical adjuncts applied in resection of glioma [9]. For example, Yellow fluorescein-guided surgery is an important intra-operative visualization technique, which can provide surgeon with information to perform the best possible resection of gliomas [11] (Fig. 1). However, the safety and recovery from surgical complications are uncertain. Targeted therapy also provides a new method for glioma treatment nowadays [12]. Due to the gradual progression of the disease, the prevention and intervention can be conducted in the early stage of the disease by detecting the target genes, the early diagnosis of glioma can gain precious time for oncologists to effect therapy treatments [13].

Up to date, there have been many studies on key genes and targeted therapy in glioma [12, 14, 15], but the key genes used for targeted therapy in glioma are far from being adequate. Chunhai Huang et al. have screened key genes related to glioma pathways by gene chip [16], and Yanyan Tang et al. have identified key genes and pathways in glioma by RNA-seq [15]. However, identification of key genes from proteomic study of glioma is under-investigated.

It is commonly accepted that cancer tissue has a hypoxic microenvironment [17], which has become a key topic of cancer physiology research and treatment for cancer [18]. The occurrence of cancer hypoxia is caused by insufficient supply of oxygen (O_2_), and then lead to cancer proliferation and deterioration. The occurrence of cancer hypoxia is strongly associated with tumor propagation, malignant progression, insufficient supply of oxygen (O_2_) in microenvironment [19, 20]. Furthermore, low-level oxygen can result in increased cell invasiveness, and promote tumor metastasis [21, 22], which ultimately leads to resistance to therapy [23]. Hypoxia has become the key topic of tumor physiology research and tumor treatment. However, the mechanism of hypoxia of glioma is still unknown and scientists are eager to explore it. As a rapidly progressing disease without effective therapies [24], understanding the adaptive mechanism of hypoxia in microenvironment might provide a shortcut for glioma treatment.

Stoichioproteomics is an emerging interdisciplinary field. By assessment of differential usage of key elements (e.g., nitrogen, N) in proteins, it provides an entire new perspective for investigating interactions of proteins evolution and environment [25]. Nutrient limitation theory suggests that natural selection caused by limited element supplies might bias to monomer usage to reduce the corresponding element costs, because amino acids and nucleotides are different in element counts [26]. This has been evidenced in plants and microbes. For example, in microbes [27–29], proteins that respond to N limitation in microbes use reduced amounts of N-rich amino acids [30]. In addition, in N rich environments, proteins have higher nitrogen levels in plants [31, 32].

At present, the research on glioma hypoxia is focused on microscopic, physiological, cellular and molecular research. Stoichioproteomic characteristics of glioma have not been studied, and whether the oxygen contents of proteins are lessened in a hypoxia microenvironment of glioma is unknown. Immunohistochemical method is the most accurate protein quantification method. At present, huge proteomic data based on immunohistochemical method offered by Human Protein Atlas project (HPA) [33] are available, which gives us an opportunity to screen key proteins associated with glioma from the proteome perspective, and provides us with a possibility to study the relationship between element content and protein expression level in hypoxia glioma cancer.

In this paper, we studied the oxygen characteristics of proteomes of glioma and normal cells in cerebral cortex, to test whether a hypoxia environment lessened proteins’ oxygen usage as predicted by the resource limitation theory. In addition, we identified the key proteins, genome locations and pathways associated with glioma. The results obtained here could be used for developing molecularly targeted therapy and precision medical treatment for glioma.

## Method

### Data resource

A case of recurrent glioblastoma multiforme (GBM) was provided by department of neurosurgery, Bishan Hospital, Chongqing. Tumor resection was performed by fluorescence visualization with a YELLOW 560 nm filter on a Pentero 900 microscope (Carl Zeiss, Oberkochen, Germany). Immunohistochemical staining with claudin-5 antibody (Invitrogen, Camarillo, CA, USA) were used for postoperative pathological examination.

Human genome sequences and annotation (version GRCh38.p7) were obtained from NCBI (ftp://ftp.ncbi.nih.gov/genomes/H_sapiens/). Proteomic data based on immunohistochemical analysis of glioma samples (with 13,083 proteins expressed) from 12 glioma patients and 4 kinds of normal cell types (Endothelial cells. Glial cells, Neuronal cells, Neuropil cells, with 12,918, 12,918, 12,919, 12,906 proteins expressed respectively) in cerebral cortex tissues from normal people were retrieved from Human Protein Atlas project (http://www.proteinatlas.org/).

### Protein expression evaluation

Protein expression evaluations were based on immunohistochemical data from HPA, which includes the assessment of staining intensity (negative, weak, medium or strong) and fraction of staining cells (< 25%, 25–75% or > 75%) (Additional file 1: Table S1). According to HPA, the degree of a protein’s expression is measured to 4 levels: not detected, low, medium and high, which were further converted to score: 0, 3, 6, 12. In order to make use of experimental data as much as possible, the expression value of 0 were replaced by 0.1 in differentially expressed proteins identification, which is a conventional processing method used in RNA-Seq [34]. In order to ensure accuracy of experiments, only protein with expression score in at least 3 glioma samples and 3 normal cell type samples were used in our study. The above procedures were realized by our in-lab developed Perl scripts.

### Principal component analysis (PCA)

PCA is a dimensionality reduction method [35], and currently it is one of the most popular methods used for judging whether several groups of samples are divisible or not, which is widely used in the field of life science research [36, 37]. In our study, PCA of expressional patterns could indicate relationships among groups of variables in a data set and show relationships that might exist between proteomes of glioma and normal cerebral cortex cells. PCA was performed by R packages “FactoMineR” [38] and “Factoextra”.

### Identification of highly expressed, lowly expressed and differentially expressed proteins (DEPs)

In proteomes of glioma and four kinds of normal cerebral cortex cells, top 1%, top 3%, top 5% proteins with high expression scores and threshold (with maximum expression score) proteins were selected as highly expressed proteins, and bottom 1%, bottom 3%, bottom 5% proteins with low expression scores and threshold (with expression score ≤0.1) were marked as lowly expressed proteins.

The expression data between glioma and four normal cerebral cortex proteomes were analyzed by “edgeR” [39] and “limma” [40] package in statistical software R (version 3.4.1) [41] to screen the differential expressed proteins. Proteins with Log2 ratio equal or greater than 1 were considered as up regulated proteins, and Log2 ratio equal or less than − 1 as down regulated proteins. *P* < 0.05 was used as a criterion for “statistically significant” and FDR level (q-value< 0.01) was used as a criterion for false positive rejections.

$$ Log2 ratio= Log2\left(\frac{theexpressionscoreofproteinsinglioma}{theexpressionscoreofproteinsinnormalcells}\right) $$

### Estimation of elemental contents on amino acid side chains

The following algorithms were used for calculating element compositions, see detail in our published paper [42].

$$ \left[\ast \mathrm{frequency}\right]=\sum \mathrm{wi}\times \mathrm{pi}/\mathrm{L} $$

In formula, wi is the number of elements in the side chain of amino acids (between 0 and 10), pi is the number of i (a amino acid) in a protein sequence, and L is the sequence length. The content of an element in multiple sequences is the average value of the element content on the side chain of amino acids in all sequences. In this study we only considered oxygen and carbon element. A carbon: oxygen ratio (C:O) was calculated as the ratio of nitrogen atoms to carbon atoms for each protein.

### Functional enrichment

Gene Set Enrichment Analysis (GSEA) (http://software.broadinstitute.org/gsea/index.jsp) is a web-based software application for integrating, analyzing, and understanding statistically significant, concordant differences between two interested biological samples [43]. KEGG annotations and enrichment for human genes were extracted and performed by GSEA.

### Statistics and visualizations

We used statistical software R (version 3.4.1) [41] for statistical analysis and packages “ggplot2” [44], “ggrepel” [45], “grid” [46] for visualization. In addition, R package “circlize” [47] was used to locate genes on genome and “Clusterprofiler” [48] was used to plot functional enrichment results.

## Results

### Principal component analysis and differentially expressed protein (DEPs) identification

**Fig. 2:**
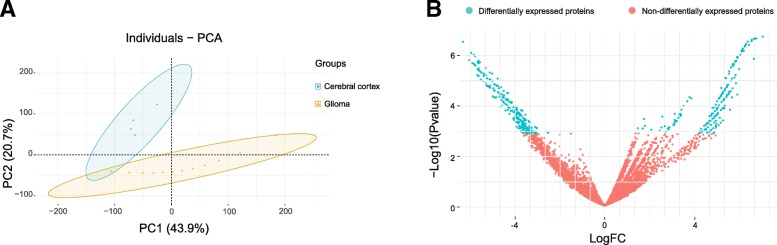
Principal components analysis (PCA) and DEPs identification. **a** PCA plot of the glioma and normal cerebral cortex proteomes. **b** DEPs identified between glioma and normal cerebral cortex proteomes

To explore the expression differences between glioma cells and normal cells, the proteomic data of 12 glioma samples were compared with that of 4 cerebral cortex cell types, using the principal components analysis (PCA) (Fig. 2). This analysis summarized the variations in glioma and cerebral cortex samples, based on the expression differences in proteins, and generated plots that separated samples. Twelve glioma samples were indicated as yellow triangles, and 4 cerebral cortex cell types were indicated as green dots. Glioma and cerebral cortex samples were clustered respectively, and 64.6% of variance was explained by the first two principal components. We found that glioma samples were separated from normal cerebral cortex samples, which revealed that glioma and cerebral cortex samples were expressional indistinguishable at the overall proteomic level (Fig. 2a), and expressional differences between them could be used for further analysis.

In order to explore the occurrence and development mechanism of glioma, differentially expressed proteins were identified between glioma and 4 normal cerebral cortex samples, with established criteria (*P* < 0.05 and FDR level < 0.01). A total of 1480 differentially expressed proteins were obtained, which consisted of 226 up regulated proteins and 1254 down regulated proteins in glioma (Additional file 1: Table S4, Fig. 2b).

### Oxygen and carbon contents in all proteins expressed in glioma and normal cerebral cortex cells

**Fig. 3:**
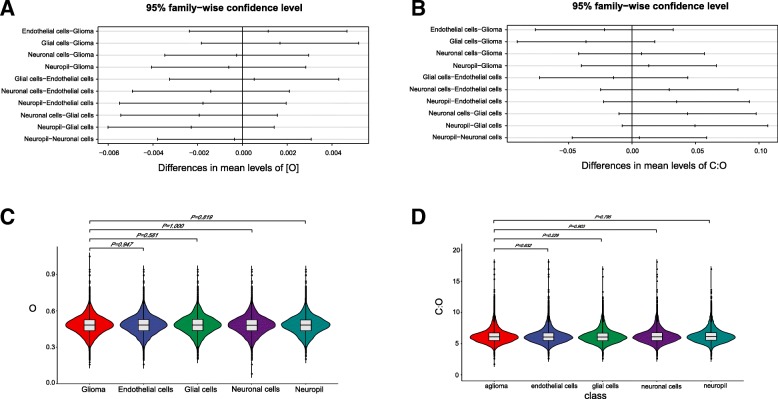
Distribution of element (oxygen and carbon) contents of all expressed proteins and its multiple variance in glioma and 4 normal cerebral cortex cells. **a** Tukey’s multiple comparisons of oxygen contents among different proteomes. **b** Tukey’s multiple comparisons of carbon contents among different proteomes. **c** Oxygen contents of all expressed proteins. **d** Carbon contents of all expressed proteins. Statistic results of Kolmogorov-Smirnov test were shown

After retrieving proteomic data of glioma and four normal cerebral cortex samples, we calculated and compared oxygen contents and C:O ratios of proteins. The average oxygen content of all expressed proteins was 0.482 in glioma, and 0.483, 0.484, 0.482, 0.481 in four normal cerebral cortex samples (Additional file 1: Table S2). No significant differences of oxygen contents and C:O ratios among the selected groups were detected (Fig. 3a-b, Additional file 1: Table S2). The average oxygen content in proteins expressed in glioma was not significantly different from that of the normal cerebral cortex samples (*P* > 0.05, Fig. 3c). The average C:O ratio of proteins expressed in glioma was also similar to that of the normal cerebral cortex samples (*P* > 0.05, Fig. 3d). Since no differences of oxygen contents were found in this step, we only analyzed oxygen contents in proteins. We did not associate it with the protein’s expression levels.

### Patterns of oxygen contents in highly and lowly expressed proteins

To better explore the association between a protein’s oxygen content and its expression level, we further calculated and compared the oxygen contents in the highly and lowly expressed proteins in glioma and four kinds of normal cerebral cortex cells. To ensure the result reliability, the highly and lowly expressed proteins were screened out based on the top/bottom 1% proteins, top/bottom 3% expressed proteins, the top/bottom 5% expressed proteins and proteins with threshold expression score of ≥maximum/≤0.1. Meanwhile the C:O ratio in each protein was calculated and used as a control for oxygen content comparisons. As the principal element of organisms, carbon is a suitable control for element composition analysis [49].

**Fig. 4:**
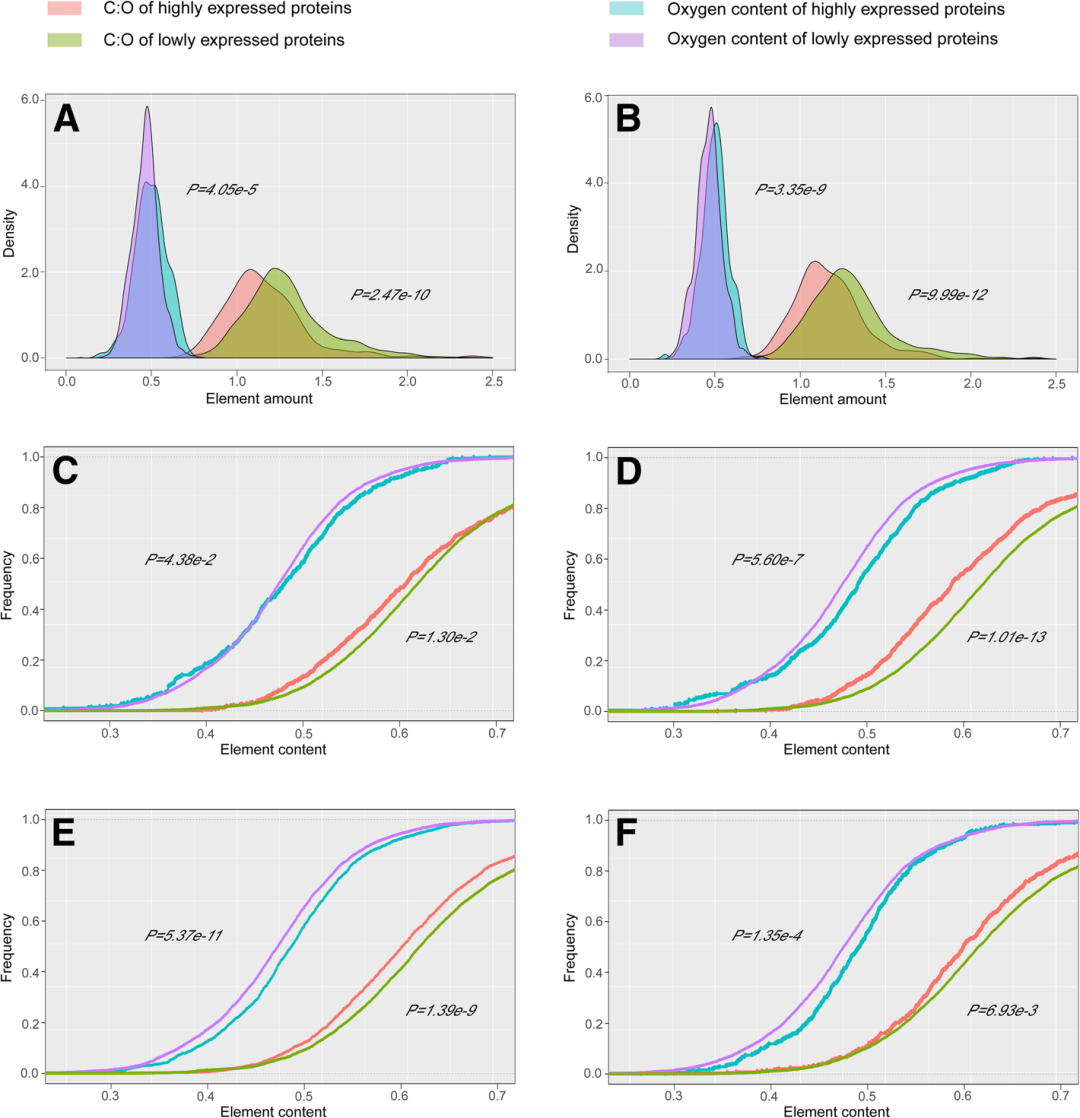
Distribution of C:O ratio of highly expressed and lowly expressed proteins in glioma and 4 cerebral cortex samples. (**a**) Glioma. (**b**) Glioma (top/bottom 3%). (**c**) Endothelial cells. (**d**) Glial cells. (**e**) Neuronal cells. (**f**) Neuropil. (For **a**,**c**,**d**,**e**,**f**, proteins with threshold expression score of ≥maximum/≤0.1 were studied. In addition, to make them better presented, the value of C:O ratio were divided by 5 in **a** and **b**, and divided by 10 in **c**, **d**, **e** and **f**

Our results showed that, in both glioma and 4 kinds of normal cerebral cortex cells, the oxygen contents of highly (proteins with maximum threshold expression score) and lowly (proteins with threshold expression score of ≤0.1) expressed proteins were significantly different (Fig. 4, Additional file 1: Table S3). Average oxygen content of highly expressed proteins is 6.65% higher than that of lowly expressed proteins in glioma, and this trend was less pronounced in endothelial, glial, neuronal, and neuropil cells (with 2.43% averaged) (Additional file 1: Table S3). Of the 5 samples considered, the distributions of oxygen content of the highly and lowly expressed proteins are entirely nonoverlapping (0.477 in the lowest highly expressed proteins vs. 0.475 for the highest lowly expressed proteins; Additional file 1: Table S3). All results were agreed for proteins expressed at the top/bottom 1, 3% or 5%, or proteins with a preset threshold score, and significant difference of oxygen contents could be detected in the majority (Fig. 4, Additional file 1: Figures S2, S3, S4, S5, S6, S7, S8, S9, S10, S11, S12, S13, S14, S15, S16).

Meanwhile the C:O ratio in each protein was calculated and used as another indicator for oxygen content changes. As carbon-to-nitrogen ratios had been used as an indicator for nitrogen limitation of plants and other organisms [50], this method could reduce the deviation caused by side chain lengths. Our results showed that the C:O ratio of highly expressed proteins (5.820, 6.291, 6.129, 6.153 and 6.117) was 4.50% lower than that of lowly expressed proteins (6.488, 6.362, 6.375, 6.390 and 6.339) in both glioma and 4 kinds of normal cerebral cortex cell types, and the C:O ratio of highly and lowly expressed proteins in both glioma and 4 normal samples were significantly different (*p* = 2.47e-10,1.30e-2, 1.01e-13, 1.39e-9 and 6.93e-3, Ks test, Additional file 1: Table S3) and their distributions were largely separated (Fig. 4).

All together, these results suggested that there was an association existed between proteins’ oxygen contents and their expression levels in glioma and four normal cerebral cortex samples. On average, oxygen content was 3.27% higher in the highly expressed proteins than that in the lowly expressed proteins.

### Patterns of oxygen contents in up regulated and down regulated proteins in glioma

**Fig. 5:**
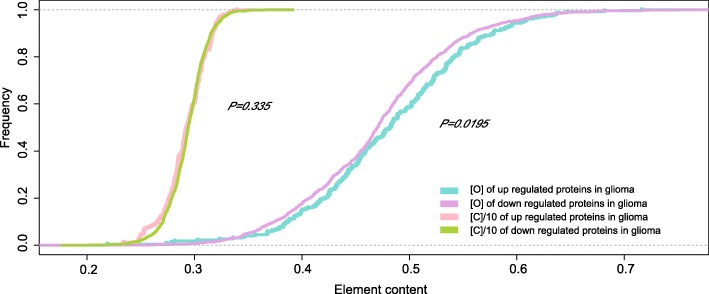
Distribution of oxygen and carbon contents of DEPs between glioma and four cerebral cortex samples. (In order to present results within the same scale, the values of carbon content were reduced by ten times)

After observing the fact that oxygen contents of the highly expressed proteins were higher than that of the lowly expressed proteins in glioma and four cerebral cortex samples, we checked whether this phenomenon existed between up regulated and down regulated proteins. After screening the differentially expressed proteins, we identified 226 up regulated and 1254 down regulated proteins in glioma. We then calculated and compared the oxygen contents in these proteins. The average oxygen content (0.481) of up regulated proteins was 2.56% higher than that (0.469) of the down regulated proteins in glioma, and the difference was statistically supported (*p* = 0.0195, Ks test, Additional file 1: Table S5). The distribution of oxygen contents in up and down regulated proteins was largely separated (Fig. 5).

Meanwhile the carbon contents were calculated and used as a control for oxygen content calculations. The average carbon contents of DEPs in glioma and cerebral cortex tissues weren’t significantly different (*p* = 0.335, Ks test, Additional file 1: Table S5). As it was shown, the distribution of carbon contents in the up and the down regulated proteins virtually overlapped, which indicated no significant carbon content differences between glioma and four cerebral cortex proteomes (Fig. 5).

### Localization of differentially expressed genes on genome

**Fig. 6:**
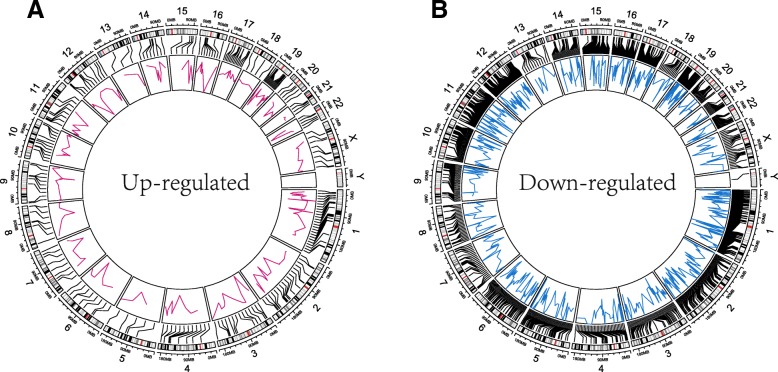
Genome localization of genes encoding DEPs. **a** Genes encoding up regulated proteins. **b** Genes encoding down regulated proteins

As described above, the oxygen contents of up regulated proteins were higher than that of the down regulated proteins in glioma, which is of great significance for deciphering the mechanism of oxygen element usage bias. Meanwhile, differentially expressed genes (DEGs) could help us to better understand the mechanism of glioma. Those genes coding the up or down regulated proteins were commonly considered as key disease-associated genes. We further determined the genome location of genes encoding these up and down regulated proteins. 226 up regulated genes and 1254 down regulated genes were respectively located on human genome (Fig. 6). Most differentially expressed genes were distributed on chromosome 1, with 25 up regulated and 108 down regulated genes respectively, following with 17 up, 68 down regulated genes on chromosome 2, and 15 up, 67 down regulated genes located on chromosome 19. Fewest differentially expressed genes were distributed on chromosome Y, with only 1 gene being located.

Among these key genes, ZSWIM5 was the most up regulated, which plays a possible role in nerve conduction formation [51], and was considered as high cytoplasmic expression gene of interest for human glioma [52]. In addition, CCR9, ADGRE5 and MEOX1 were significantly up regulated. CCR9 associated with T lymphocyte development when bound to its specific ligand, and is highly expressed in variety of cancers [53]. MEOX1 plays key role in regulating somite development, which associated with cancer progression [54]. ADGRE5 was closely related with tumor cell adhesion, migration, angiogenesis, and apoptosis [55]. These key genes play important roles in glioma proliferation.

### Functional enrichment and dissection

After locating the key genes on human genome, we further analyzed functional enrichments of these key genes, which could help us to efficiently examine large gene lists in a network context. KEGG enrichments were performed respectively for genes of up regulated proteins and down regulated proteins to check the specific pathways enriched by these two gene groups. A total of 14 pathways were enriched by genes of up regulated proteins, 62 pathways were enriched by genes of down regulated proteins, with FDR value less than 0.05 and *p* value less than 0.05 as the threshold for enrichment analysis.

**Fig. 7:**
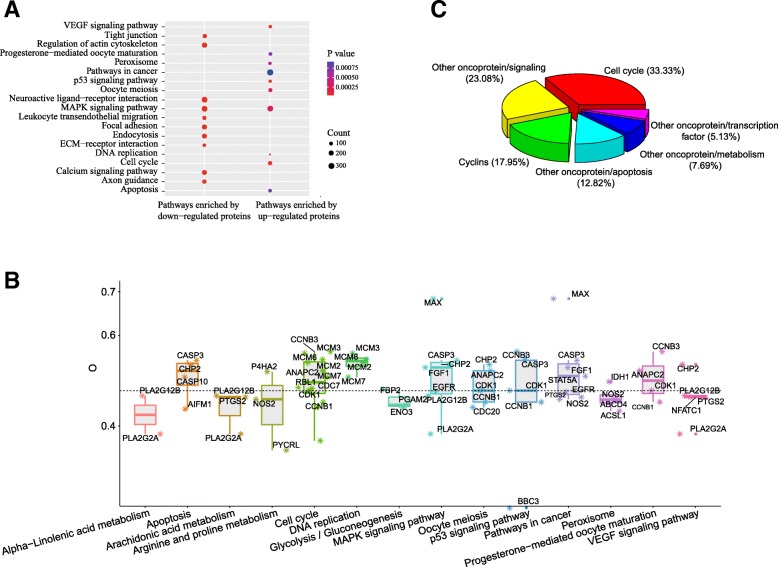
Functional dissections of DEPs. **a** KEGG enrichment of up regulated proteins and down regulated proteins. **b** Oxygen content of up regulated proteins in ten pathways enriched by up regulated proteins. **c** Functional dissection of proteins, with oxygen content equal or higher than 0.482 in up regulated proteins enriched pathways

The top 10 enriched pathways of up regulated proteins were illustrated in Fig. 7a and Additional file 1: Table S6. The most enriched pathway was cell cycle, which were enriched by 12 proteins. Other proteins were enriched in MAPK signaling pathway (7 proteins), pathways in cancer (7 proteins), p53 signaling pathway (5 proteins), VEGF signaling pathway (5 proteins), oocyte meiosis (5 proteins), DNA replication (4 proteins), peroxisome (4 proteins), progesterone-mediated oocyte maturation (4 proteins), apoptosis (4 proteins), arginine and proline metabolism (3 proteins), arachidonic acid metabolism (3 proteins), glycolysis/gluconeogenesis (3 proteins) and alpha-Linolenic acid metabolism (2 proteins). In addition, the top ten enriched pathways of down regulated proteins were presented in Fig. 7a and Additional file 1: Table S6. Twenty-eight proteins were enriched in Focal adhesion, and 26 proteins were enriched in regulation of actin cytoskeleton and neuroactive ligand-receptor interaction. Other proteins were enriched in MAPK signaling pathway (25 proteins), tight junction (21 proteins), calcium signaling pathway (21 proteins), endocytosis (20 proteins), leukocyte transendothelial migration (17 proteins), axon guidance (17 proteins) and ECM-receptor interaction (14 proteins).

These pathways enriched by up regulated proteins and down regulated proteins could help us to explain the pathogenetic mechanisms of glioma. Peroxisome is an important site for biological oxidation and energy metabolism. Up-regulation of this pathway increases the energy metabolism of glioma cells [56], which supplies adequate energy to the rapid proliferation of glioma cells. Peroxisomes are also major oxygen users and the oxygen produced from hydrogen peroxide is used within the organelle [57]. In addition, p53 signaling pathway and apoptosis have also be determined to be closely related to the occurrence of glioma. Pathways enriched by down regulated proteins suggested that neuroactive-ligand receptor interaction pathway and axon guidance were associated with the occurrence of glioma. As having been reported, the interruption of neuroactive-ligand receptor interaction pathway could cause some neuron related diseases [58]. Proper function of axon guidance is essential for avoiding neurological disorders [59]. So the disorders of neuroactive ligand-receptor interaction and axon guidance inevitably lead to glioma.

Further, we dissected these pathways enriched by genes of up regulated proteins for understanding which kind of up regulated proteins consumed more oxygen in the hypoxic microenvironment of glioma. The functions of those proteins with oxygen content higher than the average (of five proteomes, 0.482) were checked one by one manually (Fig. 7b, Additional file 1: Table S7). We found that, most of these proteins with high oxygen contents were involved in cell cycle (33.33%), followed by signaling (23.08%), cyclins (17.95%), apoptosis (12.82%), metabolism (7.69%), and transcription factor (5.13%) (Fig. 7c).

## Discussion

A total of 226 up regulated proteins and 1243 down regulated proteins were identified in glioma, and genes encoding these key proteins were located on human genome, which showed the distribution characteristics of key genes. These marker proteins could lead to new ways for glioma diagnosis and treatments. The genome location of key genes laid foundation for genome study of glioma, and allowed for studies of the upstream and the downstream regulations of key genes, which could ultimately improve the understanding of the molecular mechanism of glioma.

An association of oxygen content with protein expression level was detected in both glioma and 4 kinds of normal cerebral cortex cells, and this association was more pronounced in glioma cells. However, no carbon content differences between the highly and the lowly expressed proteins were detected. In addition, oxygen contents of up regulated proteins were 2.56% higher than that of the down regulated proteins in glioma, which was contrary to the resource limitation theory.

Resource limitation theory [30] could not be applied to human glioma cells. Since our results suggested that cancer hypoxia microenvironment didn’t lessen the oxygen contents in the up regulated proteins as predicted by the theory. On the basis of this theory, if the environment lacks a particular element, the corresponding-element-rich proteins should have reduced abundances [30]. In agreement with this theory, proteins that respond to N limitation in microbes decrease the use of N-rich amino acids [30] and proteins with high nitrogen contents in plants live in nitrogen-rich environments [31]. On the contrary, we found that in the hypoxia microenvironment of glioma, oxygen contents of highly expressed proteins were higher than that of the lowly expressed proteins. Moreover, compared with normal cerebral cortex cells, up regulated proteins’ oxygen contents were 2.56% higher than that of the down regulated proteins in glioma. These findings indicated that highly expressed and up regulated proteins did not save the use of oxygen in the glioma hypoxia microenvironment.

Functional enrichment and dissection of up regulated proteins play key roles in explaining the mechanism of oxygen usage bias. Firstly, by functional enrichment, cell cycle, oocyte meiosis DNA replication were firstly enriched out, which were closely related to cell division and cytoskeleton, and consistent with the characteristics of rapid proliferation and division of cancer cells. Unregulated cell cycle leads malignant proliferation, which is the most typical feature of cancer [60], and error-prone phenotypes in DNA replication often lead to serious, unexpected spread of cancer. Our previous studies have shown that cytoskeleton proteins possess much acidic amino acids and high oxygen contents [26]. Rapid proliferation and division of tumor cells require frequent dissociation and recombination of cytoskeleton. Up-regulation of these pathways consume more oxygen, which cause oxygen usage bias in glioma cells. Secondly, functional dissection of up regulated proteins with high oxygen contents showed that 51.28% of the proteins were involved in cell cycle and cyclins, which further suggested that proteins involved in cell cycle and cyclins were up regulated and consumed more oxygen in glioma cells.

We attempted to explain the molecular mechanism of oxygen usage bias detected in glioma cells from a function point of view. The oxygen content differences of up regulated and down regulated proteins might be closely related to their functions. More oxygen consumed by the up regulated proteins could be explained by the need for faster division and numerous activities of cytoskeleton. In addition, large amount of oxygen consumed by the up regulated proteins might have played a role in aggravating hypoxia microenvironment in glioma tissue. Deviated from resource limitation theory, the increased amount of oxygen consumed by the up regulated proteins was not caused by a hypoxic microenvironment. On the contrary, the increased amount of oxygen consumed by the up regulated proteins promoted the formation of a hypoxic microenvironment in glioma.

KEGG enrichment of DEPs helps to clarify the molecular mechanism of glioma. VEGF signaling pathway is a key to initiate tumor angiogenesis [61], and new vessels to transport sufficient nutrition and oxygen for tumor cell, which ensures the stately physiological activity of the tumor cell [62]. Studies have shown that VEGF-A signaling pathway is an effective target for therapy, and the tumor-related diagnosis could be studied according to endothelial growth factor (VEGF) family [63].

Focal adhesion and tight junction play critical roles in adhesion [64], and down-regulation of focal adhesion and tight junction pathways decreased the adhesion between glioma cells and rendered cells enhanced migration abilities. Tight junction and leukocyte transendothelial migration play vital roles in immunity surveillance [65, 66]. Down regulation of these pathways helped glioma cells proliferate in the body without being eliminated by the immune system.

Interestingly, MAPK signaling pathway was enriched by both up regulated and down regulated proteins in our study. Constitutive activating MAPK signaling pathway often leads to promotion of abnormal cell growth and tumorigenesis [67]. However, suppressing the MAPK signaling pathway could not only suppresses migration and invasion of malignant glioma cells, but also inhibit the viability and promote the senescence and apoptosis of glioma cells [68, 69]. This could explain why this pathway was repeated enriched.

## Conclusion

In this report, stoichioproteomic characteristics of proteome in glioma and 4 normal cerebral cortex cells were studied. We found that the oxygen contents of highly expressed proteins were 3.27% higher than that of lowly expressed proteins in all samples. Besides, we identified 226 up regulated proteins and 1254 down regulated proteins in glioma, and found that oxygen contents of up regulated proteins were 2.56% higher than that of down regulated proteins, which was contrary to the resource limitation theory. Genome location of key genes showed that most of the gens were located on chromosome 1, and least on chromosome Y. Pathway enrichment results were closely related to proliferation, energy and immune, such as cell cycle, peroxisome, focal adhesion and so on. Functional dissection showed that 51.28% of the up regulated proteins with high oxygen contents were involved in cell cycle and cyclins. Our discovery on oxygen content bias in glioma proteomes will provide new insights in studies of hypoxic microenvironment and disease-related biological macromolecules. Key proteins and pathways detected in this study will play crucial roles in revealing the molecular mechanism of glioma and oxygen usages bias, and establish a foundation for application of stoichioproteomics in precision medicine.

## Additional file

Additional file 1: **Figure S1.** Flow chart of the entire experimental process. **Figure S2.** Distribution of oxygen and C:O ratio of top/bottom 1% expressed proteins in glioma. **Figure S3.** Distribution of oxygen and C:O ratio of top/bottom 1% expressed proteins in endothelial cells. **Figure S4.** Distribution of oxygen and C:O ratio of top/bottom 1% expressed proteins in glial cells. **Figure S5.** Distribution of oxygen and C:O ratio of top/bottom 1% expressed proteins in neuronal cells. **Figure S6.** Distribution of oxygen and C:O ratio of top/bottom 1% expressed proteins in neuropil. **Figure S7.** Distribution of oxygen and C:O ratio of top/bottom 3% expressed proteins in glioma. **Figure S8.** Distribution of oxygen and C:O ratio of top/bottom 3% expressed proteins in endothelial cells. **Figure S9.** Distribution of oxygen and C:O ratio of top/bottom 3% expressed proteins in glial cells. **Figure S10.** Distribution of oxygen and C:O ratio of top/bottom 3% expressed proteins in neuronal cells. **Figure S11.** Distribution of oxygen and C:O ratio of top/bottom 3% expressed proteins in neuropil. **Figure S12.** Distribution of oxygen and C:O ratio of top/bottom 5% expressed proteins in glioma. **Figure S13.** Distribution of oxygen and C:O ratio of top/bottom 5% expressed proteins in endothelial cells. **Figure S14.** Distribution of oxygen and C:O ratio of top/bottom 5% expressed proteins in glial cells. **Figure S15.** Distribution of oxygen and C:O ratio of top/bottom 5% expressed proteins in neuronal cells. **Figure S16.** Distribution of oxygen and C:O ratio of top/bottom 5% expressed proteins in neuropil cells. **Table S1.** Evaluation of protein expression scores. **Table S2.** Oxygen content and C:O ratio of all proteins expressed in glioma and normal cerebral cortex. **Table S3.** Oxygen content and C:O ratio of highly and lowly expressed proteins in glioma and normal cerebral cortex. **Table S4.** Up and down regulated proteins in glioma. **Table S5.** Oxygen content (O) and carbon content (C) of up regulated proteins and down regulated proteins in glioma. **Table S6.** Pathways enriched by genes encoding up regulated and down regulated proteins. **Table S7.** Functional dissection of up regulated proteins in glioma. (DOCX 1952 kb)

## Data Availability

The dataset analyzed in the current study are available in the [Human Protein Atlas project] repository, [http://www.proteinatlas.org/]. The human genome sequences and annotation (version GRCh38.p7) used in the current study are available in the [National Center of Biotechnology Information] repository, [ftp://ftp.ncbi.nih.gov/genomes/H_sapiens/].

## Abbreviations

C: Carbon content
C:O ratio: The ratio of carbon content to oxygen content
DEGs: Differentially expressed genes
DEPs: Differentially expressed proteins
O: Oxygen content
