# Supplementary material for: Stoichioproteomics reveal oxygen usage bias, key proteins and pathways in glioma
Source: BMC Med Genomics. 2019 Aug 29;12:125. doi: 10.1186/s12920-019-0571-y (PMC6716898; doi:10.1186/s12920-019-0571-y)
Supplement: Supplementary file 1 — Figure S1. Flow chart of the entire experimental process. Figure S2. Distribution of oxygen and C:O ratio of top/bottom 1% expressed proteins in glioma. Figure S3. Distribution of oxygen and C:O ratio of top/bottom 1% expressed proteins in endothelial cells. Figure S4. Distribution of oxygen and C:O ratio of top/bottom 1% expressed proteins in glial cells. Figure S5. Distribution of oxygen and C:O ratio of top/bottom 1% expressed proteins in neuronal cells. Figure S6. Distribution of oxygen and C:O ratio of top/bottom 1% expressed proteins in neuropil. Figure S7. Distribution of oxygen and C:O ratio of top/bottom 3% expressed proteins in glioma. Figure S8. Distribution of oxygen and C:O ratio of top/bottom 3% expressed proteins in endothelial cells. Figure S9. Distribution of oxygen and C:O ratio of top/bottom 3% expressed proteins in glial cells. Figure S10. Distribution of oxygen and C:O ratio of top/bottom 3% expressed proteins in neuronal cells. Figure S11. Distribution of oxygen and C:O ratio of top/bottom 3% expressed proteins in neuropil. Figure S12. Distribution of oxygen and C:O ratio of top/bottom 5% expressed proteins in glioma. Figure S13. Distribution of oxygen and C:O ratio of top/bottom 5% expressed proteins in endothelial cells. Figure S14. Distribution of oxygen and C:O ratio of top/bottom 5% expressed proteins in glial cells. Figure S15. Distribution of oxygen and C:O ratio of top/bottom 5% expressed proteins in neuronal cells. Figure S16. Distribution of oxygen and C:O ratio of top/bottom 5% expressed proteins in neuropil cells. Table S1. Evaluation of protein expression scores. Table S2. Oxygen content and C:O ratio of all proteins expressed in glioma and normal cerebral cortex. Table S3. Oxygen content and C:O ratio of highly and lowly expressed proteins in glioma and normal cerebral cortex. Table S4. Up and down regulated proteins in glioma. Table S5. Oxygen content (O) and carbon content (C) of up regulated proteins and down regulated proteins [file 12920_2019_571_MOESM1_ESM.docx]

Supplementary Figures and Tables for

**Stoichioproteomics reveal oxygen usage bias, key proteins and pathways in glioma**

Yongqin Yin^1#^, Bo Li^1#^, Kejie Mou^2#^, Muhammad T. Khan^3,4^, Aman C. Kaushik^3^, Dongqing Wei^3^*, Yu-Juan Zhang^1^*

*Correspondence:

Prof. Yu-Juan Zhang, College of Life Sciences, Chongqing Normal University, University city, Chongqing 401331, PR China, Tel/Fax: 86-23-65910315; e-mail: zhangyj@cqnu.edu.cn

Prof. Dongqing Wei, Shanghai Jiao Tong University, China, e-mail: dqwei@sjtu.edu.cn

**This PDF file includes**

Figures. S1 to S16.

Tables. S1 to S7.

**Figures**


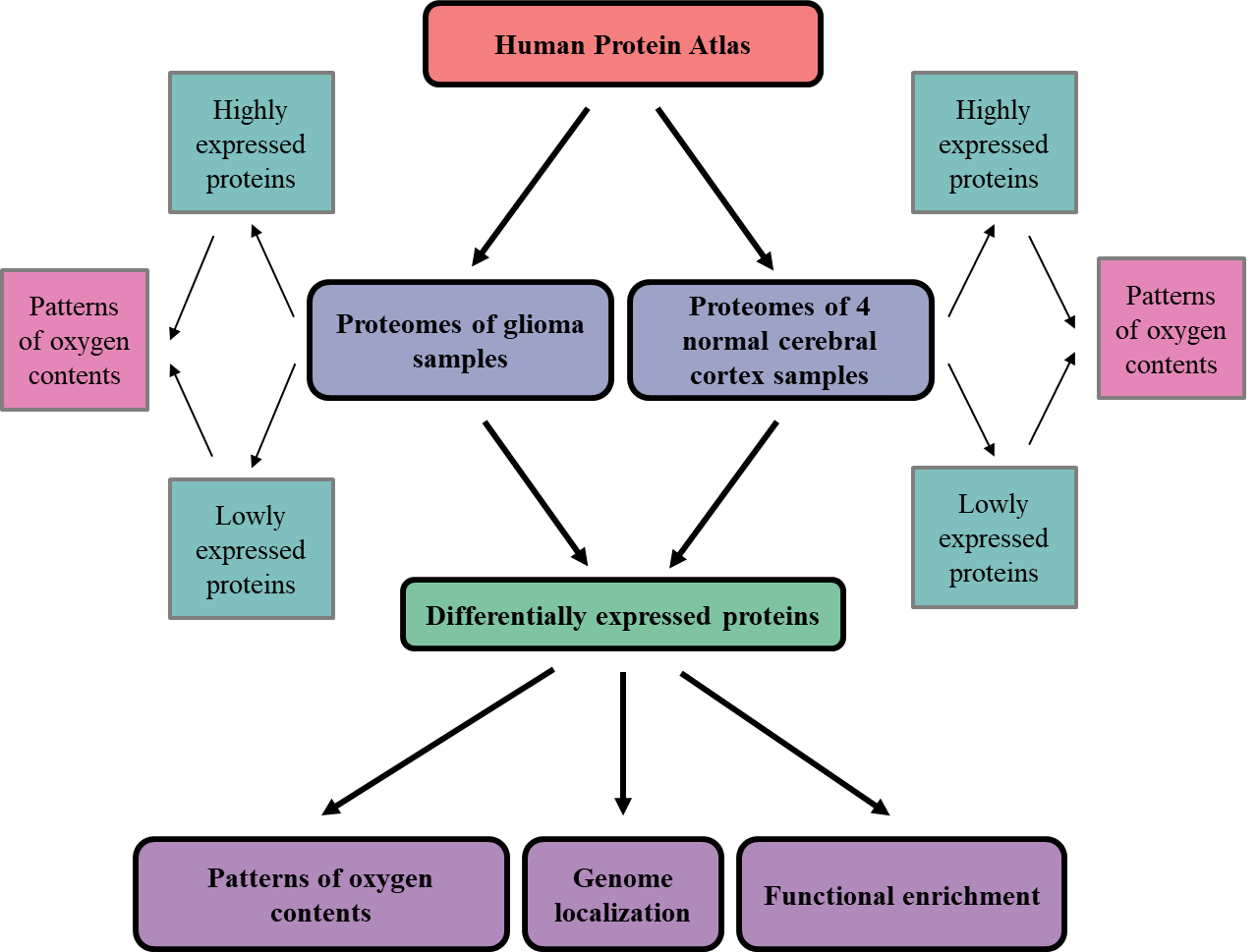


**Figure S1.** Flow chart of the entire experimental process.

**
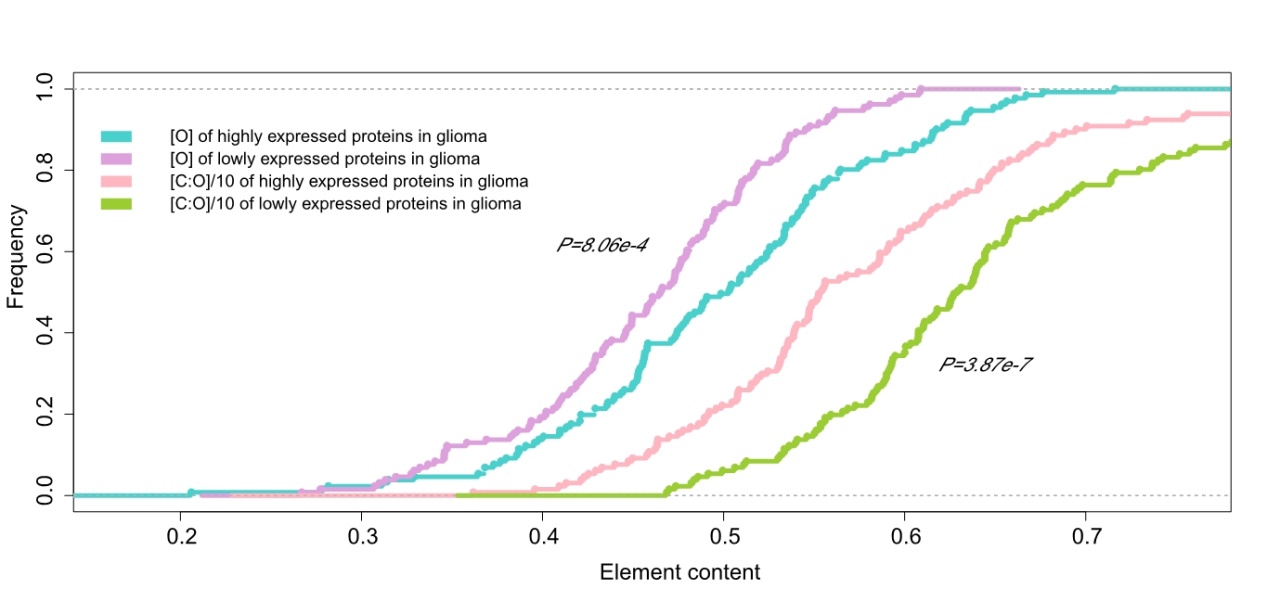
**

**Figure S2.** Distribution of oxygen and C:O ratio of top/bottom 1% expressed proteins in glioma.

**
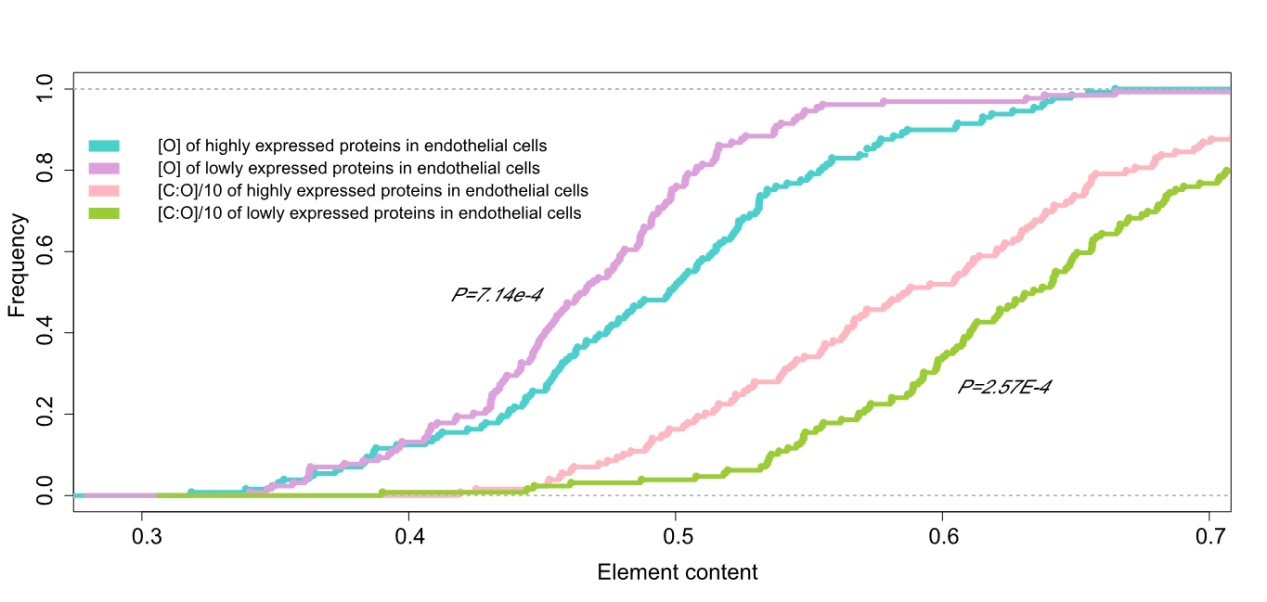
**

**Figure S3.** Distribution of oxygen and C:O ratio of top/bottom 1% expressed proteins in endothelial cells.

**
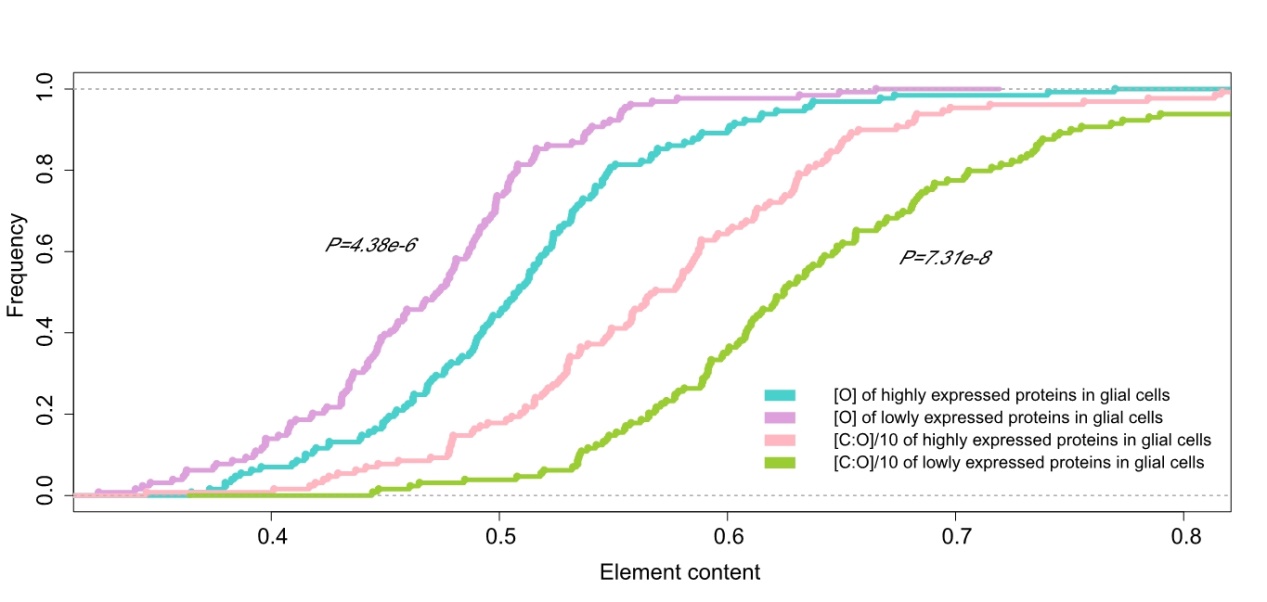
**

**Figure S4.** Distribution of oxygen and C:O ratio of top/bottom 1% expressed proteins in glial cells.

**
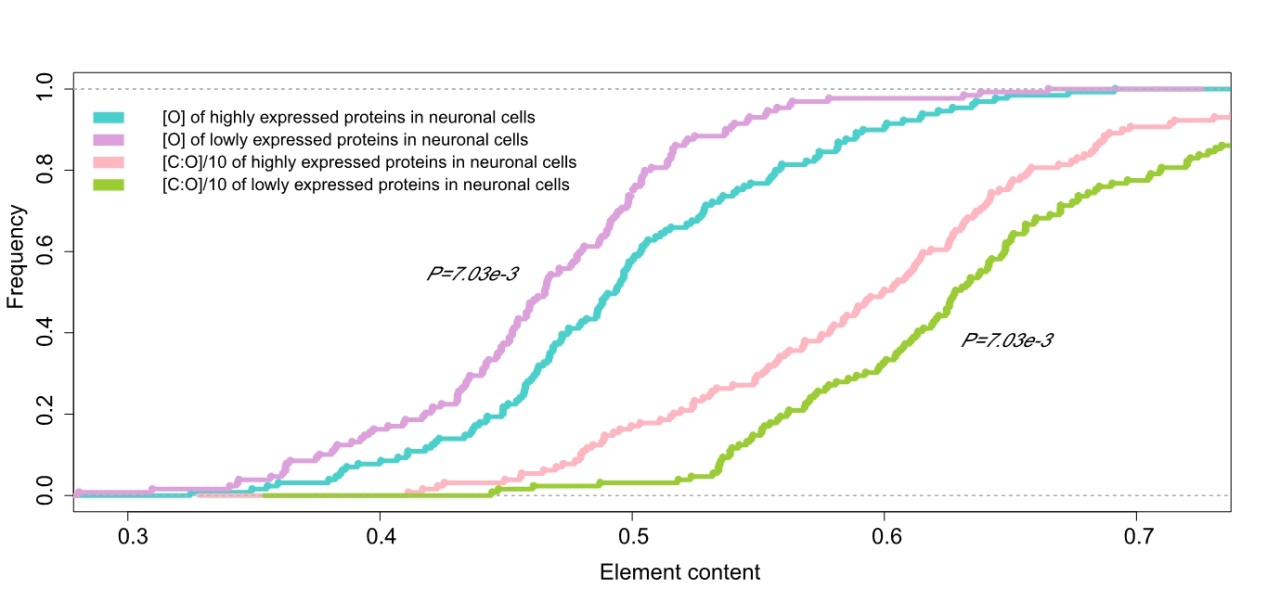
**

**Figure S5.** Distribution of oxygen and C:O ratio of top/bottom 1% expressed proteins in neuronal cells.

**
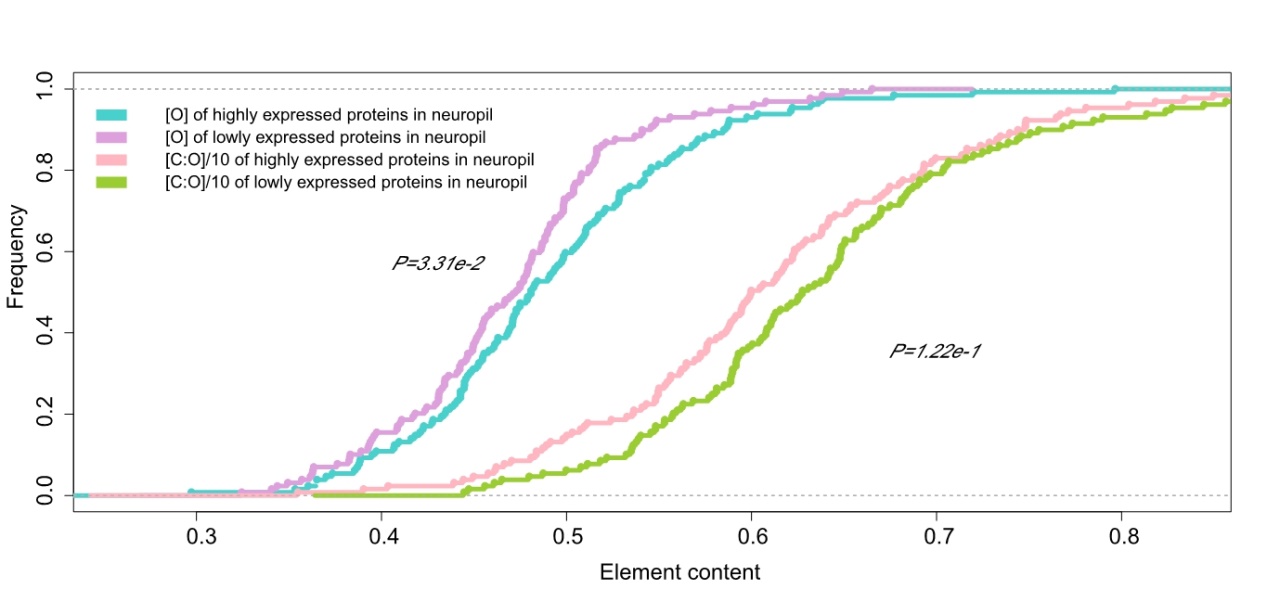
**

**Figure S6.** Distribution of oxygen and C:O ratio of top/bottom 1% expressed proteins in neuropil.

**
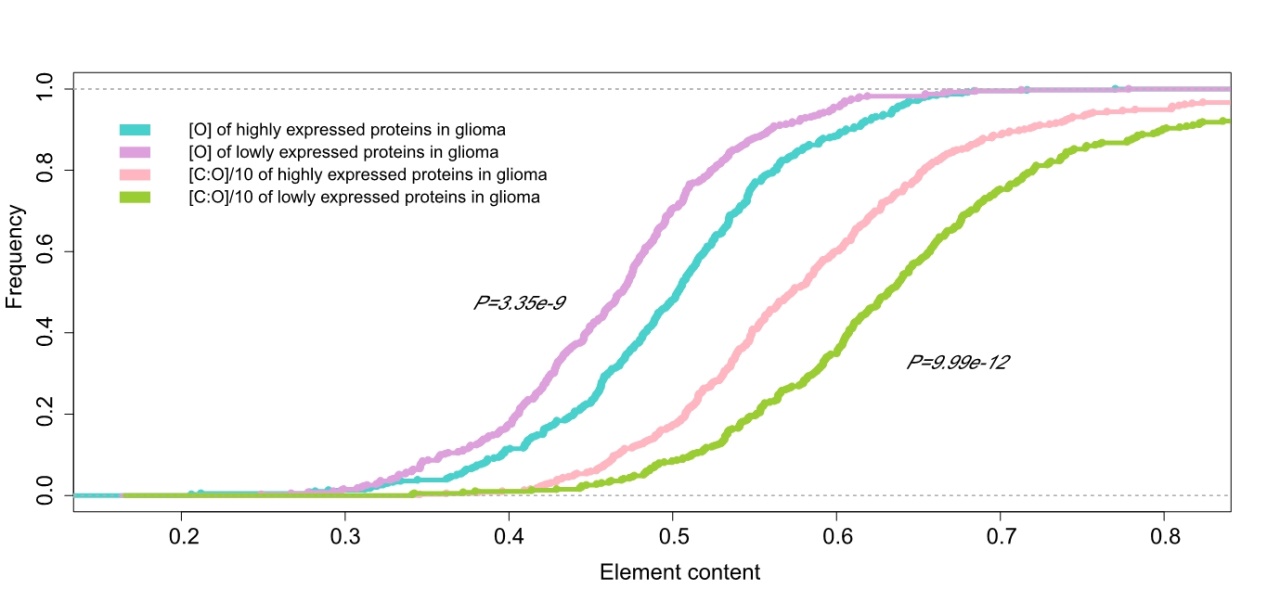
**

**Figure S7.** Distribution of oxygen and C:O ratio of top/bottom 3% expressed proteins in glioma.

**
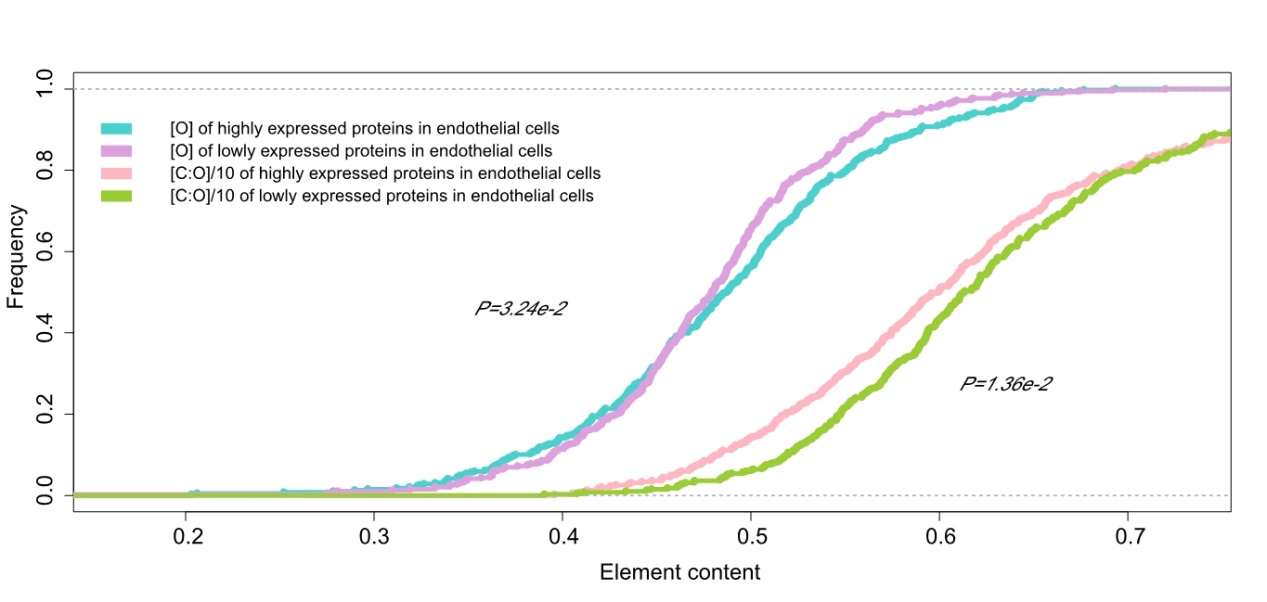
**

**Figure S8.** Distribution of oxygen and C:O ratio of top/bottom 3% expressed proteins in endothelial cells.

**
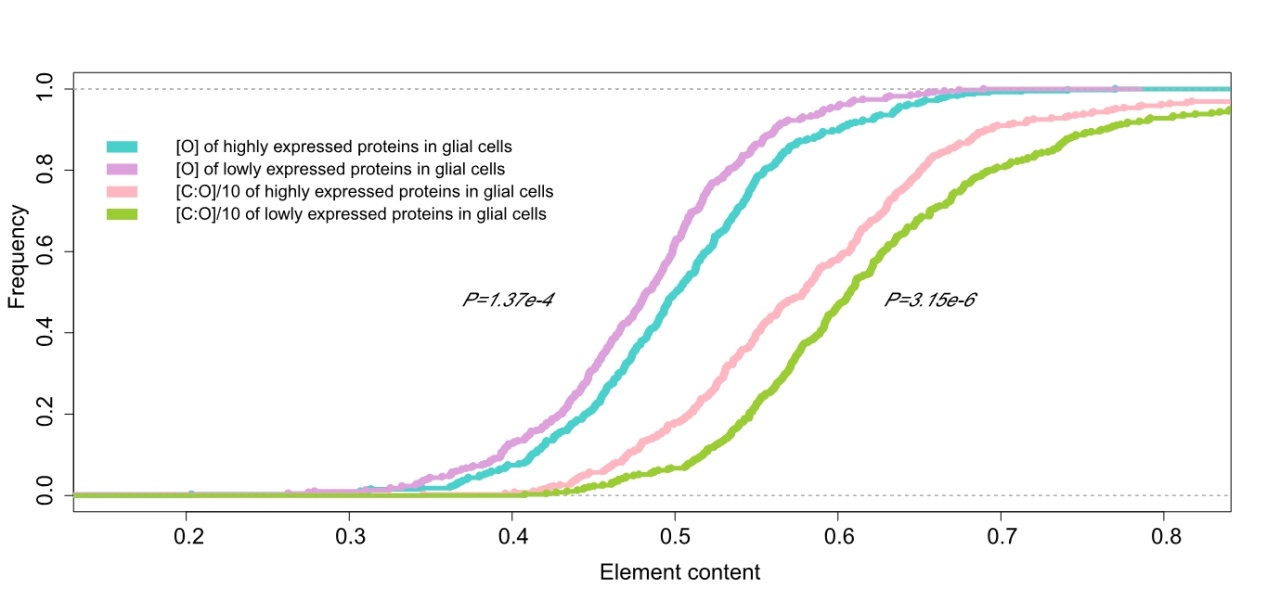
**

**Figure S9.** Distribution of oxygen and C:O ratio of top/bottom 3% expressed proteins in glial cells.

**
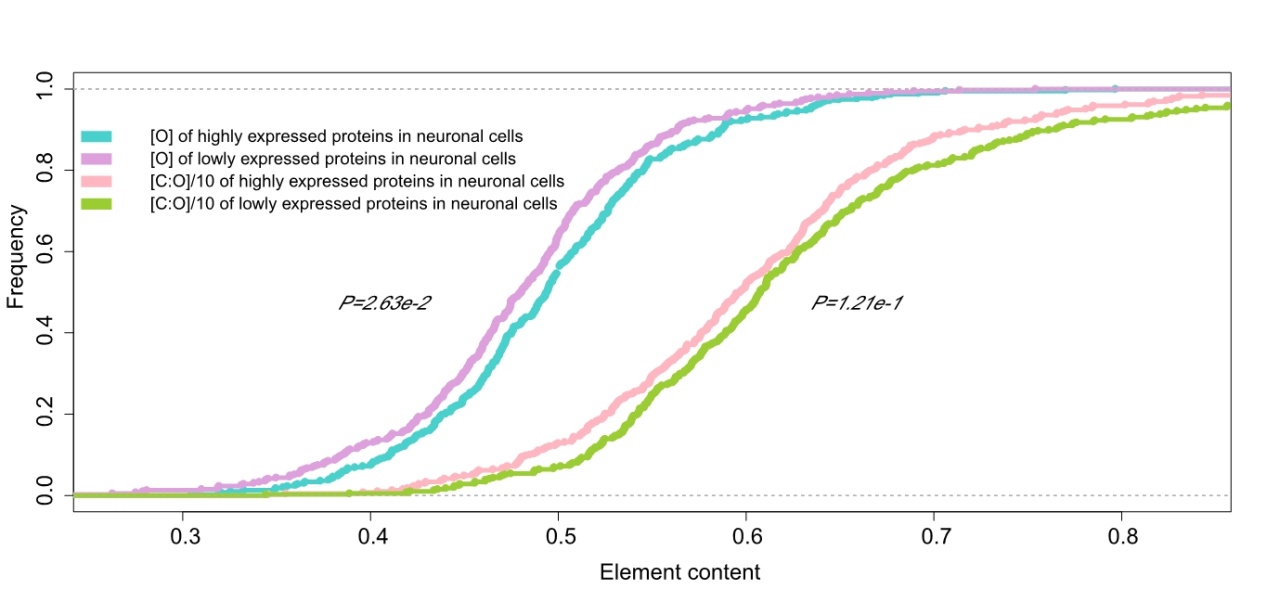
**

**Figure S10.** Distribution of oxygen and C:O ratio of top/bottom 3% expressed proteins in neuronal cells.

**
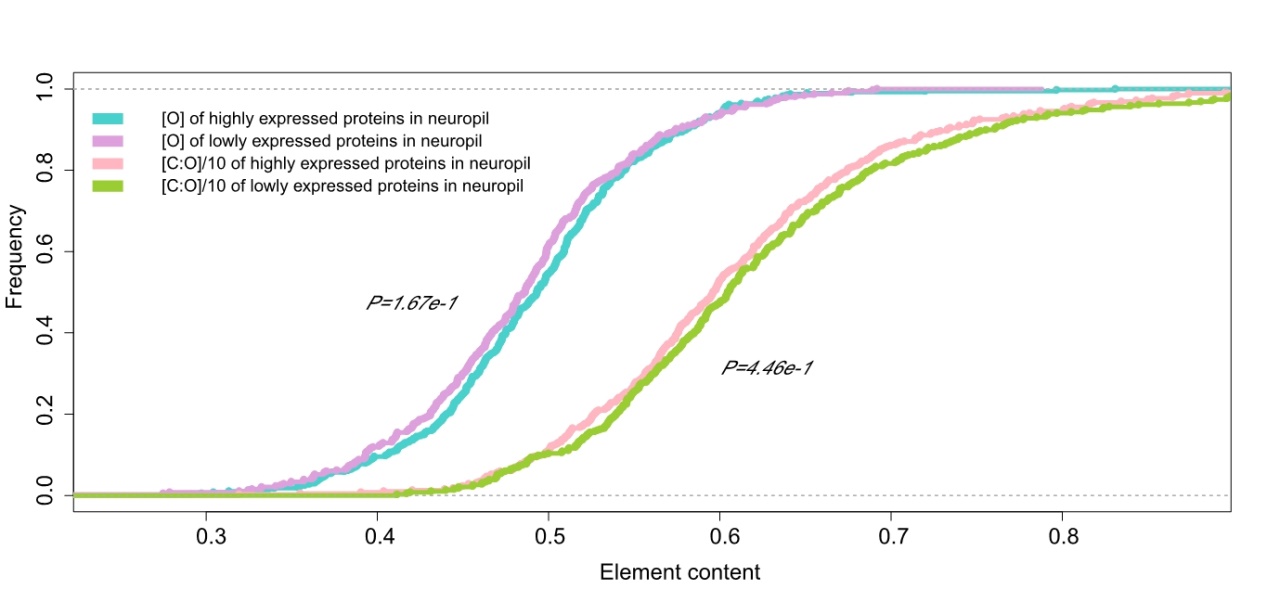
**

**Figure S11.** Distribution of oxygen and C:O ratio of top/bottom 3% expressed proteins in neuropil.

**
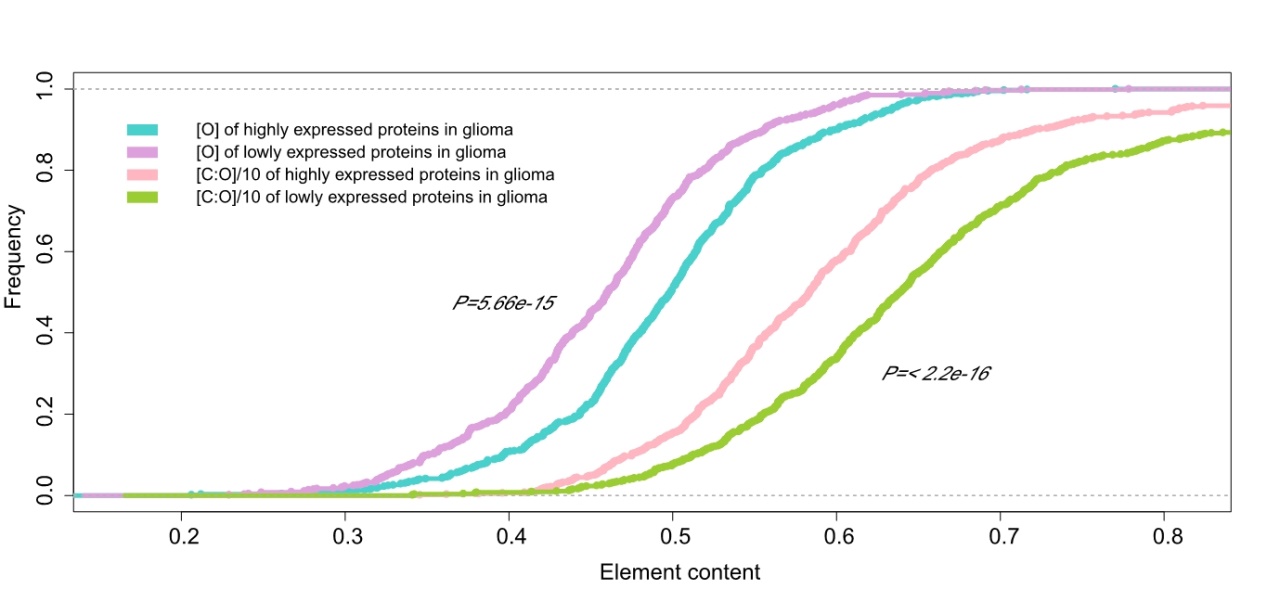
**

**Figure S12.** Distribution of oxygen and C:O ratio of top/bottom 5% expressed proteins in glioma.

**
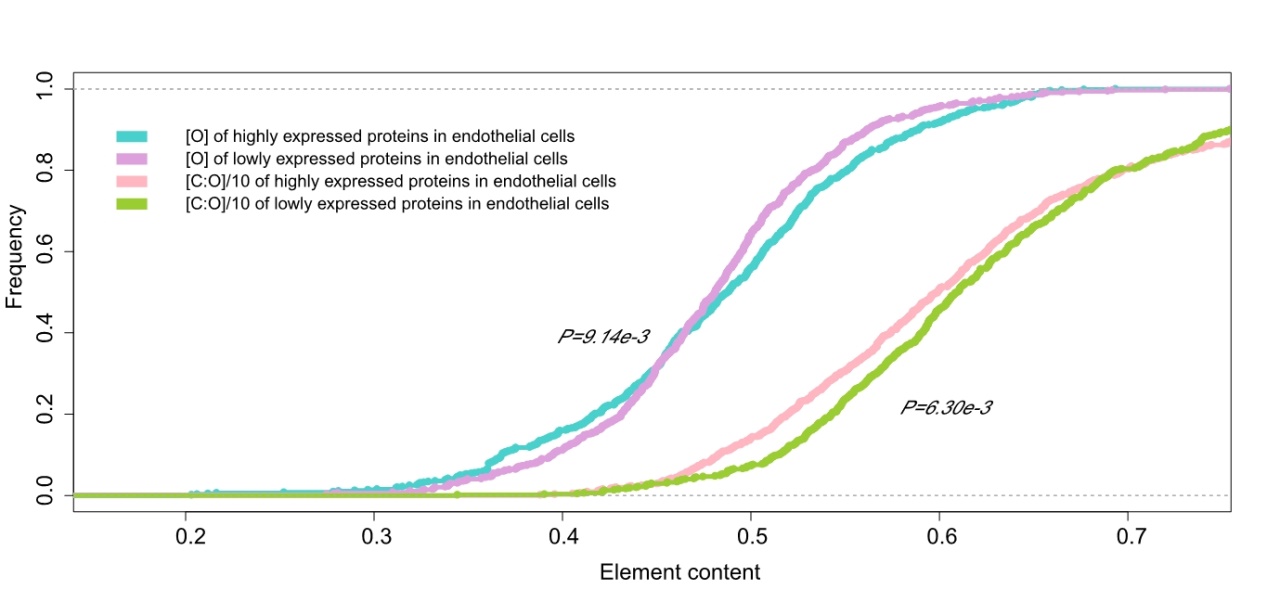
**

**Figure S13.** Distribution of oxygen and C:O ratio of top/bottom 5% expressed proteins in endothelial cells.

**
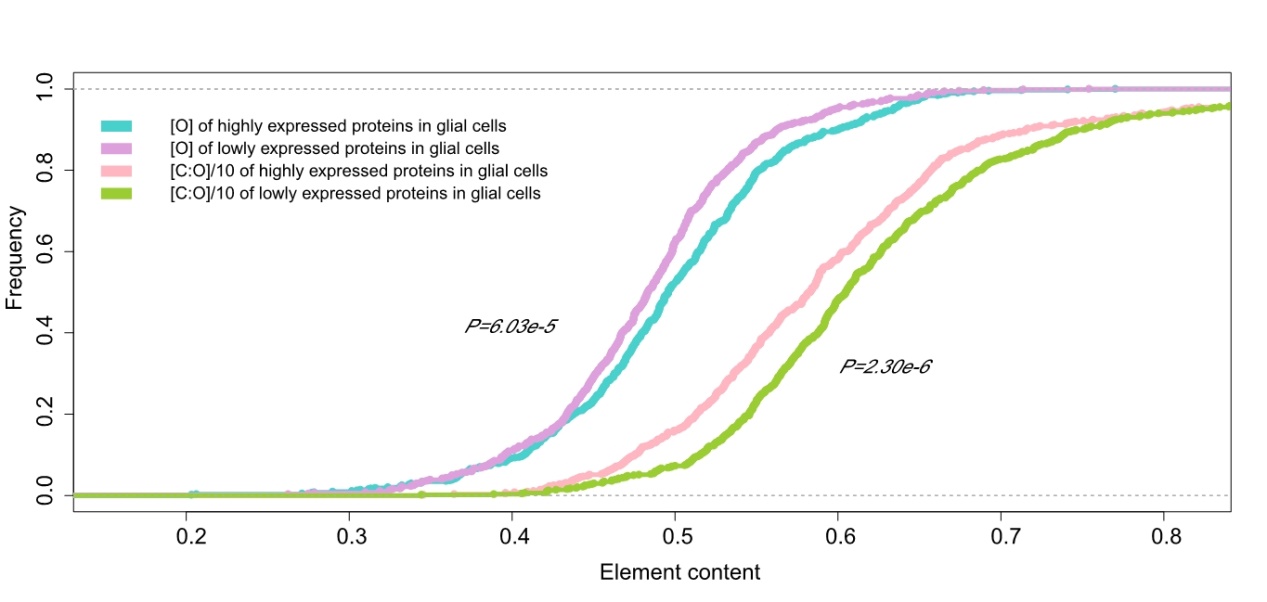
**

**Figure S14.** Distribution of oxygen and C:O ratio of top/bottom 5% expressed proteins in glial cells.

**
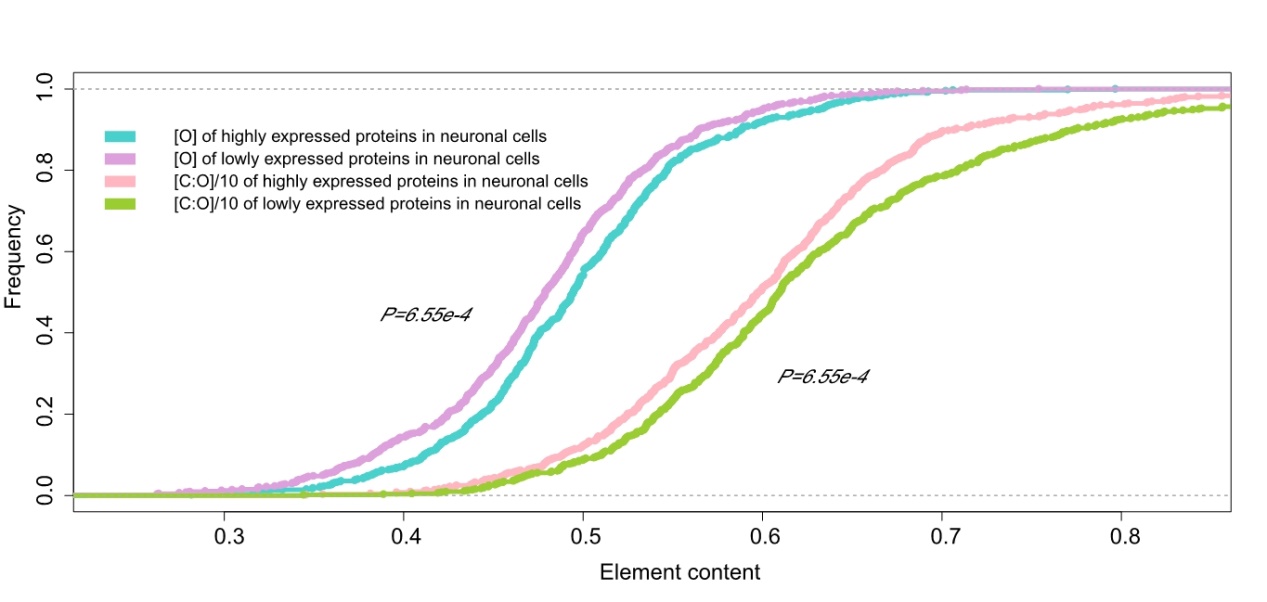
**

**Figure S15.** Distribution of oxygen and C:O ratio of top/bottom 5% expressed proteins in neuronal cells.

**
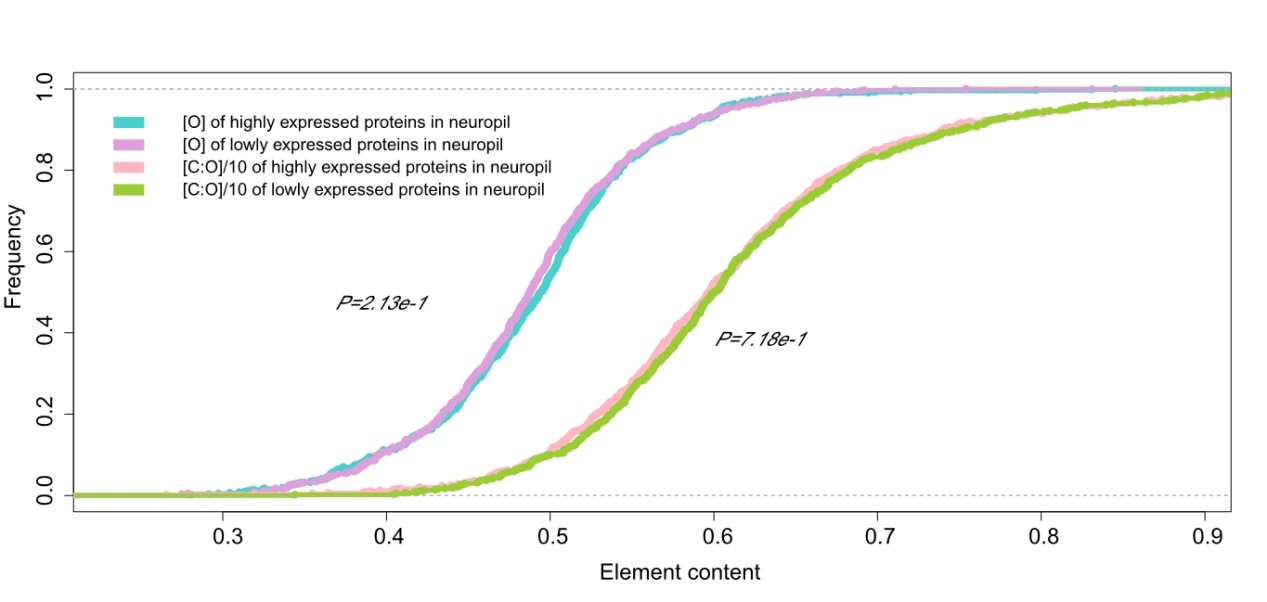
**

**Figure S16.** Distribution of oxygen and C:O ratio of top/bottom 5% expressed proteins in neuropil.

**Tables**

**Table S1. Evaluation of protein expression scores.**

| **Staining intensity** | **Staining cells** | **Degree** | **Expression score** |
| --- | --- | --- | --- |
| Negative | — | Not detected | 0 |
| Weak | <25% | Low | 3*0.625=1.875 |
|  | 25-75% or >75% |  |  |
| Moderate | <25% | Medium | 6*0.625=3.75 |
|  | 25-75% or >75% |  |  |
| Strong | <25% | High | 12*0.625=7.5 |
|  | 25-75% or >75% |  |  |

**Table S2. Oxygen content and C:O ratio of all proteins expressed in glioma and cerebral cortex.**

|  | **Number of genes** | **Mean [O]** | **Mean [C：O]** |
| --- | --- | --- | --- |
| Endothelial cells | 5925 | 0.483 | 6.205 |
| Glial cells | 5975 | 0.484 | 6.191 |
| Neuronal cells | 8323 | 0.482 | 6.234 |
| Neuropil | 6384 | 0.481 | 6.24 |
| Glioma | 8187 | 0.482 | 6.227 |
| P value(K-S test) | —— | Endothelial cells & Glioma 0.9474 Glial cells & Glioma 0.5814 Neuronal cells & Glioma 0.9999 Neuropil & Glioma 0.8192 | Endothelial cells & Glioma 0.6315 Glial cells & Glioma 0.2288 Neuronal cells & Glioma 0.9027 Neuropil & Glioma 0.7953 |
| P value  (Wilcoxon test) | —— | Endothelial cells & Glioma 0.4528 Glial cells & Glioma 0.1891 Neuronal cells & Glioma 0.7635 Neuropil & Glioma 0.5903 | Endothelial cells & Glioma 0.226 Glial cells & Glioma 0.05028 Neuronal cells & Glioma 0.6872 Neuropil & Glioma 0.1848 |

**Table S3. Oxygen (O) content and C:O ratio of highly and lowly expressed proteins in glioma and cerebral cortex.**

| **Sample** | **Number of proteins** | **Scale** | **Highly expressed proteins** | | | **Lowly expressed proteins** | | | **Comparsion of [O] (P value) (Kolmogorov-Smirnov test)** | **Comparsion of [O] (P value) (Wilcoxon test)** | **Comparsion of [C:O] (P value) (Kolmogorov-Smirnov test)** | **Comparsion of [C:O] (P value) (Wilcoxon test)** | **Ratio of mean [O] increase of highly expressed proteins （proteins with maximum threshold expression score）to that of lowly expressed proteins （proteins with threshold expression score of ≤0.1）** |
| --- | --- | --- | --- | --- | --- | --- | --- | --- | --- | --- | --- | --- | --- |
|  |  |  | **Number of selected proteins** | **Mean [O]** | **Mean [C:O]** | **Number of selected proteins** | **Mean [O]** | **Mean [C:O** |  |  |  |  |  |
| glioma | 13083 | 1% | 131 | 0.497 | 5.810 | 131 | 0.458 | 6.547 | 8.06e-4 | 2.06e-4 | 3.87e-7 | 4.244e-8 | 6.65% |
|  |  | 3% | 392 | 0.499 | 5.878 | 392 | 0.465 | 6.475 | 3.35e-9 | 1.39e-10 | 9.99e-12 | 2.18e-14 |  |
|  |  | 5% | 654 | 0.497 | 5.961 | 654 | 0.457 | 6.593 | 5.66e-15 | < 2.2e-16 | < 2.2e-16 | < 2.2e-16 |  |
|  |  | highly expressed proteins: expression=0.1lowly expressed proteins:expression=12 | 127 | 0.497 | 5.820 | 3127 | 0.466 | 6.488 | 4.05e-5 | 4.26e-5 | 2.47e-10 | 3.99e-11 |  |
| endothelial cells | 12918 | 1% | 129 | 0.494 | 6.024 | 129 | 0.467 | 6.418 | 7.14e-4 | 9.40e-4 | 2.57e-4 | 1.75e-4 | 2.43% |
|  |  | 3% | 388 | 0.485 | 6.175 | 388 | 0.477 | 6.338 | 3.24e-2 | 1.50e-1 | 1.36e-2 | 9.34e-3 |  |
|  |  | 5% | 646 | 0.483 | 6.196 | 646 | 0.480 | 6.274 | 9.14e-3 | 1.73e-1 | 6.30e-3 | 2.00e-2 |  |
|  |  | highly expressed proteins: expression=0.1 lowly expressed proteins:expression=7.5 | 488 | 0.477 | 6.291 | 6993 | 0.474 | 6.362 | 4.38e-2 | 2.21e-1 | 1.30e-2 | 2.14e-2 |  |
| glial cells | 12918 | 1% | 129 | 0.508 | 5.721 | 129 | 0.468 | 6.408 | 4.38e-6 | 1.20e-6 | 7.31e-8 | 5.88e-9 |  |
|  |  | 3% | 388 | 0.502 | 5.876 | 388 | 0.479 | 6.295 | 1.37e-4 | 3.03e-5 | 3.15e-6 | 6.53e-8 |  |
|  |  | 5% | 646 | 0.497 | 5.951 | 646 | 0.482 | 6.234 | 6.03e-5 | 1.32e-4 | 2.30e-6 | 6.78e-8 |  |
|  |  | highly expressed proteins: expression=0.1 lowly expressed proteins:expression=7.5 | 800 | 0.486 | 6.129 | 6943 | 0.474 | 6.375 | 5.60e-7 | 2.04e-7 | 1.01e-13 | 3.30e-13 |  |
| neuronal cells | 12919 | 1% | 129 | 0.498 | 5.964 | 129 | 0.464 | 6.455 | 7.03e-3 | 1.50e-4 | 7.03e-3 | 3.13e-4 |  |
|  |  | 3% | 388 | 0.496 | 6.021 | 388 | 0.479 | 6.273 | 2.63e-2 | 3.41e-3 | 1.21e-1 | 1.74e-2 |  |
|  |  | 5% | 646 | 0.497 | 6.012 | 646 | 0.478 | 6.302 | 6.55e-4 | 1.76e-5 | 6.55e-4 | 1.43e-4 |  |
|  |  | highly expressed proteins: expression=0.1 lowly expressed proteins:expression=7.5 | 2050 | 0.488 | 6.153 | 4596 | 0.472 | 6.390 | 5.37e-11 | 9.80e-13 | 1.39e-9 | 8.15e-13 |  |
| Neuropil | 12906 | 1% | 129 | 0.490 | 6.120 | 129 | 0.469 | 6.374 | 3.31e-2 | 3.00e-2 | 1.22e-1 | 3.95e-2 |  |
|  |  | 3% | 387 | 0.492 | 6.069 | 387 | 0.484 | 6.227 | 1.67e-1 | 9.66e-2 | 4.46e-1 | 1.37e-1 |  |
|  |  | 5% | 645 | 0.491 | 6.099 | 645 | 0.487 | 6.169 | 2.13e-1 | 2.86e-1 | 7.18e-1 | 3.66E-1 |  |
|  |  | highly expressed proteins: expression=0.1 lowly expressed proteins:expression=7.5 | 487 | 0.490 | 6.117 | 6522 | 0.475 | 6.339 | 1.35e-4 | 5.24e-5 | 6.93e-3 | 1.18e-3 |  |

**Table S4. Up and down regulated proteins.**

| **ID** | **name** | **KO** | **logFC** | **logCPM** | **LR** | **PValue** | **FDR** | **O** | **C** |
| --- | --- | --- | --- | --- | --- | --- | --- | --- | --- |
| ENSG00000140718 | FTO | K19469 | 1.4736041 | 7.946524931 | 11.218 | 0.000810077 | 0.0075823 | 0.5425743 | 3.0970297 |
| ENSG00000170275 | CRTAP | K19606 | 1.6935952 | 8.244287315 | 11.174363 | 0.000829354 | 0.0077312 | 0.5137157 | 3.2219451 |
| ENSG00000079387 | SENP1 | K08592 | 1.7492562 | 7.948827905 | 12.851494 | 0.00033721 | 0.0043689 | 0.5481366 | 2.9875776 |
| ENSG00000100325 | ASCC2 | K18667 | 1.7922604 | 8.335946995 | 11.645886 | 0.000643447 | 0.0070064 | 0.5640687 | 3.0356671 |
| ENSG00000118513 | MYB | K09420 | 2.079368 | 7.818357157 | 10.969974 | 0.000926001 | 0.008494 | 0.5335085 | 2.9093298 |
| ENSG00000145375 | SPATA5 | K14575 | 2.4475256 | 7.536420277 | 10.824339 | 0.001001745 | 0.0091148 | 0.4826428 | 2.8331467 |
| ENSG00000169756 | LIMS1 | - | 2.5289276 | 7.574056145 | 11.361829 | 0.00074969 | 0.0074949 | 0.4031008 | 3.0361757 |
| ENSG00000136383 | ALPK3 | K08868 | 2.5696899 | 8.270010648 | 13.226296 | 0.000276049 | 0.0036385 | 0.4735186 | 2.5207132 |
| ENSG00000132780 | NASP | K11291 | 2.5696899 | 8.270010648 | 13.226091 | 0.000276079 | 0.0036385 | 0.715736 | 2.5850254 |
| ENSG00000188321 | ZNF559 | K09228 | 2.6710805 | 8.160961367 | 11.602049 | 0.000658792 | 0.0071396 | 0.4883721 | 3.0598007 |
| ENSG00000110987 | BCL7A | - | 2.7332247 | 7.477426795 | 10.690169 | 0.001077064 | 0.0095882 | 0.6363636 | 2.4761905 |
| ENSG00000188938 | FAM120AOS | - | 2.8171319 | 7.556280108 | 11.207315 | 0.000814755 | 0.0076013 | 0.28125 | 2.7304688 |
| ENSG00000115233 | PSMD14 | K03030 | 2.8258986 | 7.564527472 | 11.215045 | 0.000811368 | 0.0075882 | 0.4516129 | 2.9193548 |
| ENSG00000137947 | GTF2B | K03124 | 2.8452738 | 7.582680333 | 11.051444 | 0.000886182 | 0.0081877 | 0.4746835 | 2.7879747 |
| ENSG00000173436 | MINOS1 | K17784 | 2.8819282 | 7.617309516 | 11.409086 | 0.000730858 | 0.0074832 | 0.3717949 | 3.0897436 |
| ENSG00000125952 | MAX | K04453 | 3.0634189 | 6.922008096 | 11.791324 | 0.000595074 | 0.0065544 | 0.68125 | 2.78125 |
| ENSG00000183060 | LYSMD4 | - | 3.1007384 | 6.957317367 | 11.832861 | 0.000581945 | 0.0064286 | 0.4141414 | 2.8080808 |
| ENSG00000080839 | RBL1 | K04681 | 3.1007384 | 6.957317367 | 11.832774 | 0.000581972 | 0.0064286 | 0.4850187 | 3.0243446 |
| ENSG00000184436 | THAP7 | - | 3.1019079 | 7.465979626 | 12.162542 | 0.000487589 | 0.0054972 | 0.433657 | 2.8252427 |
| ENSG00000104320 | NBN | K10867 | 3.1175274 | 8.166338491 | 13.688221 | 0.000215804 | 0.0029318 | 0.5702918 | 2.9124668 |
| ENSG00000186591 | UBE2H | K10576 | 3.1249175 | 7.847619928 | 12.639767 | 0.000377627 | 0.0047009 | 0.5956284 | 3.0382514 |
| ENSG00000097046 | CDC7 | K02214 | 3.147655 | 7.002080954 | 12.406957 | 0.000427737 | 0.0049044 | 0.4825784 | 2.8972125 |
| ENSG00000112893 | MAN2A1 | K01231 | 3.1555977 | 7.517063345 | 12.427624 | 0.00042303 | 0.0048602 | 0.5017483 | 3.145979 |
| ENSG00000162430 | SEPN1 | K19874 | 3.1718689 | 7.024987493 | 12.372742 | 0.000435647 | 0.0049901 | 0.4661017 | 2.9983051 |
| ENSG00000134905 | CARS2 | K01883 | 3.2123466 | 7.063468491 | 12.474841 | 0.00041247 | 0.0047531 | 0.4237589 | 2.9007092 |
| ENSG00000003400 | CASP10 | K04400 | 3.2834558 | 7.131369957 | 13.024962 | 0.000307366 | 0.0040185 | 0.5076628 | 3.0076628 |
| ENSG00000114030 | KPNA1 | K15042 | 3.3404231 | 7.185976719 | 13.667289 | 0.000218223 | 0.0029576 | 0.5223048 | 2.9275093 |
| ENSG00000109738 | GLRB | K05196 | 3.3772911 | 7.712465465 | 13.355171 | 0.000257711 | 0.00342 | 0.4426559 | 3.1348089 |
| ENSG00000145725 | PPIP5K2 | K13024 | 3.3874604 | 7.230872889 | 13.670129 | 0.000217893 | 0.0029566 | 0.5211268 | 2.9710485 |
| ENSG00000116726 | PRAMEF12 | - | 3.3912972 | 7.801446681 | 13.472727 | 0.000242056 | 0.0032348 | 0.4161491 | 3.0559006 |
| ENSG00000139946 | PELI2 | K11964 | 3.4196982 | 7.262040051 | 14.426966 | 0.000145701 | 0.0026567 | 0.4642857 | 2.8404762 |
| ENSG00000105327 | BBC3 | K10132 | 3.5123308 | 7.351022239 | 15.061564 | 0.000104061 | 0.002226 | 0.2183908 | 2.3256705 |
| ENSG00000168066 | SF1 | K13095 | 3.6776904 | 7.510288863 | 15.882764 | 6.74E-05 | 0.0016606 | 0.3670134 | 2.7117385 |
| ENSG00000134201 | GSTM5 | K00799 | 3.7729153 | 7.602358609 | 16.609512 | 4.59E-05 | 0.0013705 | 0.4954128 | 3.3899083 |
| ENSG00000196132 | MYT1 | - | 3.8563843 | 7.682962996 | 16.375644 | 5.19E-05 | 0.0014528 | 0.6263066 | 2.6210801 |
| ENSG00000176879 | OR51G1 | K04257 | 4.2295531 | 5.41399293 | 10.726068 | 0.001056367 | 0.0095372 | 0.3021807 | 3.2647975 |
| ENSG00000177354 | C10orf71 | - | 4.2411743 | 5.426590171 | 10.443389 | 0.001230892 | 0.0096206 | 0.5337979 | 2.71777 |
| ENSG00000151790 | TDO2 | K00453 | 4.2411744 | 5.426590171 | 10.443782 | 0.00123063 | 0.0096206 | 0.5320197 | 3.2980296 |
| ENSG00000188425 | NANOS2 | K18760 | 4.2411745 | 5.426590171 | 10.443987 | 0.001230494 | 0.0096206 | 0.3985507 | 2.7608696 |
| ENSG00000131375 | CAPN7 | K08576 | 4.2874576 | 5.47057206 | 11.14105 | 0.000844381 | 0.0078458 | 0.500615 | 3.1328413 |
| ENSG00000138400 | MDH1B | - | 4.2874578 | 5.47057206 | 11.14166 | 0.000844103 | 0.0078458 | 0.4826255 | 3.1023166 |
| ENSG00000103522 | IL21R | K05075 | 4.3387825 | 5.520734268 | 10.587018 | 0.001138845 | 0.0095882 | 0.4888476 | 2.9163569 |
| ENSG00000156009 | MAGEA8 | - | 4.3502058 | 5.532154351 | 10.74456 | 0.001045863 | 0.0094499 | 0.5628931 | 2.8867925 |
| ENSG00000129151 | BBOX1 | K00471 | 4.3502058 | 5.532154351 | 10.744628 | 0.001045825 | 0.0094499 | 0.501292 | 3.1912145 |
| ENSG00000166387 | PPFIBP2 | - | 4.3502059 | 5.532154351 | 10.744874 | 0.001045686 | 0.0094499 | 0.533105 | 2.9166667 |
| ENSG00000168679 | SLC16A4 | K08181 | 4.4276555 | 5.606678764 | 11.678398 | 0.0006323 | 0.0069093 | 0.3921971 | 3.1396304 |
| ENSG00000164305 | CASP3 | K02187 | 4.4276555 | 5.606678764 | 11.678486 | 0.00063227 | 0.0069093 | 0.5451264 | 3.0072202 |
| ENSG00000115935 | WIPF1 | K19475 | 4.452337 | 5.631255408 | 11.0069 | 0.000907733 | 0.0083532 | 0.345098 | 2.4882353 |
| ENSG00000140993 | TIGD7 | - | 4.452337 | 5.631255408 | 11.00709 | 0.00090764 | 0.0083532 | 0.5118397 | 3.132969 |
| ENSG00000138413 | IDH1 | K00031 | 4.4673917 | 5.645845041 | 11.036559 | 0.000893325 | 0.0082404 | 0.4975845 | 3.0193237 |
| ENSG00000163110 | PDLIM5 | - | 4.4673918 | 5.645845041 | 11.036982 | 0.000893121 | 0.0082404 | 0.4688 | 2.736 |
| ENSG00000113578 | FGF1 | K18496 | 4.4969693 | 5.673776234 | 11.783616 | 0.000597543 | 0.006569 | 0.5290323 | 3.0064516 |
| ENSG00000164494 | PDSS2 | K12505 | 4.5052456 | 5.682220084 | 11.003657 | 0.000909323 | 0.0083544 | 0.4536341 | 2.8796992 |
| ENSG00000164708 | PGAM2 | K01834 | 4.505246 | 5.682220084 | 11.004843 | 0.000908741 | 0.0083544 | 0.4466403 | 3.0316206 |
| ENSG00000105671 | DDX49 | K14778 | 4.5114884 | 5.688817611 | 11.075814 | 0.00087461 | 0.0080873 | 0.4223602 | 3.0082816 |
| ENSG00000125207 | PIWIL1 | K02156 | 4.5442158 | 5.719952733 | 12.058751 | 0.000515498 | 0.0057722 | 0.4599303 | 3.0836237 |
| ENSG00000119688 | ABCD4 | K05678 | 4.5442159 | 5.719952733 | 12.059104 | 0.000515401 | 0.0057722 | 0.4570957 | 3.140264 |
| ENSG00000114790 | ARHGEF26 | K13744 | 4.5556234 | 5.731966687 | 10.488885 | 0.001200948 | 0.0095882 | 0.52124 | 2.8840413 |
| ENSG00000063515 | GSC2 | K09325 | 4.5885518 | 5.763614306 | 11.400134 | 0.000734388 | 0.0074849 | 0.2780488 | 2.5219512 |
| ENSG00000184007 | PTP4A2 | K18041 | 4.5888846 | 5.763237684 | 12.149408 | 0.000491034 | 0.0055306 | 0.4251497 | 3.0958084 |
| ENSG00000103310 | ZP2 | K19927 | 4.5891649 | 5.764736054 | 10.56593 | 0.001151911 | 0.0095882 | 0.4832215 | 2.8885906 |
| ENSG00000130479 | MAP1S | K10429 | 4.5893028 | 5.764341118 | 11.402196 | 0.000733573 | 0.0074832 | 0.4249292 | 2.6515581 |
| ENSG00000151726 | ACSL1 | K01897 | 4.589303 | 5.764341118 | 11.402931 | 0.000733283 | 0.0074832 | 0.4326648 | 3.0057307 |
| ENSG00000122477 | LRRC39 | - | 4.6280954 | 5.800999061 | 12.047445 | 0.000518634 | 0.0057905 | 0.5073746 | 3.1474926 |
| ENSG00000112486 | CCR6 | K04181 | 4.6596781 | 5.832368213 | 12.49197 | 0.000408705 | 0.0047288 | 0.3930481 | 3.1898396 |
| ENSG00000123570 | RAB9B | K07900 | 4.6596783 | 5.832368213 | 12.492741 | 0.000408536 | 0.0047288 | 0.5671642 | 2.9701493 |
| ENSG00000122180 | MYOG | K18483 | 4.6596784 | 5.832368213 | 12.493095 | 0.000408459 | 0.0047288 | 0.53125 | 2.8928571 |
| ENSG00000163817 | SLC6A20 | K05048 | 4.6671329 | 5.838920599 | 13.276899 | 0.000268697 | 0.0035576 | 0.3952703 | 3.1486486 |
| ENSG00000105894 | PTN | K16642 | 4.6674829 | 5.841067691 | 10.676844 | 0.00108485 | 0.0095882 | 0.4345238 | 2.8869048 |
| ENSG00000050405 | LIMA1 | - | 4.6888597 | 5.860682249 | 12.510819 | 0.000404602 | 0.0047009 | 0.6065789 | 2.8065789 |
| ENSG00000124207 | CSE1L | K18423 | 4.6888598 | 5.860682249 | 12.511183 | 0.000404523 | 0.0047009 | 0.4850669 | 3.161689 |
| ENSG00000187258 | NPSR1 | K08376 | 4.6888598 | 5.860682249 | 12.511416 | 0.000404473 | 0.0047009 | 0.4384615 | 3.2461538 |
| ENSG00000129646 | QRICH2 | - | 4.6888599 | 5.860682249 | 12.511547 | 0.000404445 | 0.0047009 | 0.4768491 | 2.6987372 |
| ENSG00000138592 | USP8 | K11839 | 4.7114983 | 5.882639281 | 12.520889 | 0.000402427 | 0.0047009 | 0.5635063 | 2.9991055 |
| ENSG00000176566 | DCAF4L2 | - | 4.7243276 | 5.895825814 | 10.749512 | 0.001043068 | 0.0094469 | 0.4202532 | 2.8911392 |
| ENSG00000134057 | CCNB1 | K05868 | 4.7324423 | 5.903762804 | 11.795218 | 0.000593831 | 0.006547 | 0.4526559 | 2.9722864 |
| ENSG00000138375 | SMARCAL1 | K14440 | 4.7324425 | 5.903762804 | 11.795787 | 0.000593649 | 0.006547 | 0.4769392 | 2.9308176 |
| ENSG00000126561 | STAT5A | K11223 | 4.7329673 | 5.904741329 | 10.942697 | 0.000939733 | 0.0086131 | 0.5100756 | 3.070529 |
| ENSG00000010671 | BTK | K07370 | 4.7419122 | 5.911695564 | 13.407345 | 0.000250641 | 0.0033378 | 0.5151515 | 3.1428571 |
| ENSG00000112818 | MEP1A | K01395 | 4.7419124 | 5.911695564 | 13.408103 | 0.00025054 | 0.0033378 | 0.5155039 | 3.0594315 |
| ENSG00000112118 | MCM3 | K02541 | 4.7666412 | 5.936128231 | 13.741197 | 0.000209802 | 0.0029001 | 0.5603751 | 2.8991794 |
| ENSG00000106809 | OGN | K08126 | 4.7666413 | 5.936128231 | 13.74168 | 0.000209748 | 0.0029001 | 0.5234899 | 3.1577181 |
| ENSG00000130957 | FBP2 | K03841 | 4.7666413 | 5.936128231 | 13.741838 | 0.00020973 | 0.0029001 | 0.480826 | 2.820059 |
| ENSG00000188100 | FAM25A | - | 4.7940713 | 5.963384818 | 12.858935 | 0.000335872 | 0.0043565 | 0.505618 | 2.494382 |
| ENSG00000189090 | FAM25G | - | 4.7940713 | 5.963384818 | 12.859217 | 0.000335821 | 0.0043565 | 0.505618 | 2.494382 |
| ENSG00000073756 | PTGS2 | K11987 | 4.7940714 | 5.963384818 | 12.859354 | 0.000335797 | 0.0043565 | 0.4635762 | 3.1705298 |
| ENSG00000167754 | KLK5 | K09617 | 4.8004247 | 5.968519119 | 14.700099 | 0.00012604 | 0.0024494 | 0.4163823 | 2.7337884 |
| ENSG00000115163 | CENPA | K11495 | 4.8198821 | 5.987920285 | 13.780231 | 0.000205487 | 0.0029001 | 0.3357143 | 3.0571429 |
| ENSG00000182533 | CAV3 | K12959 | 4.8326587 | 6.002265444 | 11.211826 | 0.000812777 | 0.007589 | 0.4238411 | 3.2317881 |
| ENSG00000144554 | FANCD2 | K10891 | 4.8326588 | 6.002265444 | 11.212111 | 0.000812652 | 0.007589 | 0.507138 | 3.057104 |
| ENSG00000111639 | MRPL51 | K17432 | 4.8530157 | 6.020830489 | 12.973755 | 0.000315888 | 0.0041206 | 0.2734375 | 3.3125 |
| ENSG00000122779 | TRIM24 | K08881 | 4.8558529 | 6.025132657 | 10.395878 | 0.00126297 | 0.0097978 | 0.5133333 | 2.8428571 |
| ENSG00000073111 | MCM2 | K02540 | 4.8681378 | 6.037321124 | 10.5168 | 0.001182941 | 0.0095882 | 0.539823 | 2.9048673 |
| ENSG00000117399 | CDC20 | K03363 | 4.8770853 | 6.044956134 | 12.224989 | 0.000471537 | 0.0053373 | 0.4408818 | 2.7875752 |
| ENSG00000108515 | ENO3 | K01689 | 4.8894257 | 6.05534563 | 14.879676 | 0.000114591 | 0.0022981 | 0.4400922 | 2.8018433 |
| ENSG00000181013 | C17orf47 | - | 4.8894259 | 6.05534563 | 14.880864 | 0.000114519 | 0.0022981 | 0.5017544 | 2.8421053 |
| ENSG00000130038 | CRACR2A | K17199 | 4.8894259 | 6.05534563 | 14.880999 | 0.000114511 | 0.0022981 | 0.5896033 | 2.9411765 |
| ENSG00000131196 | NFATC1 | K04446 | 4.9040973 | 6.07093398 | 13.252472 | 0.000272221 | 0.0035959 | 0.4623542 | 2.7264051 |
| ENSG00000156709 | AIFM1 | K04727 | 4.9040975 | 6.07093398 | 13.253192 | 0.000272116 | 0.0035959 | 0.4371941 | 2.8287113 |
| ENSG00000136542 | GALNT5 | K00710 | 4.9055366 | 6.071731461 | 14.195167 | 0.000164793 | 0.0027109 | 0.4521277 | 3.0148936 |
| ENSG00000188257 | PLA2G2A | K01047 | 4.9105917 | 6.077352458 | 13.339697 | 0.000259846 | 0.0034444 | 0.3819444 | 2.8055556 |
| ENSG00000109586 | GALNT7 | K00710 | 4.9317593 | 6.09816303 | 13.388221 | 0.000253209 | 0.0033681 | 0.4703196 | 3.1506849 |
| ENSG00000184924 | PTRHD1 | - | 4.9465423 | 6.113325388 | 11.583047 | 0.000665558 | 0.0071993 | 0.3785714 | 3.0571429 |
| ENSG00000147082 | CCNB3 | K05868 | 4.9473807 | 6.11212924 | 15.3004 | 9.17E-05 | 0.0020258 | 0.5641577 | 3.0358423 |
| ENSG00000181788 | SIAH2 | K08742 | 4.9495242 | 6.115898248 | 11.344497 | 0.000756719 | 0.0074949 | 0.382716 | 2.6666667 |
| ENSG00000072682 | P4HA2 | K00472 | 4.9529158 | 6.119745035 | 11.593854 | 0.000661701 | 0.0071644 | 0.5196262 | 3.0598131 |
| ENSG00000076003 | MCM6 | K02542 | 4.9622 | 6.12834859 | 12.516944 | 0.000403278 | 0.0047009 | 0.546894 | 2.9658953 |
| ENSG00000109686 | SH3D19 | - | 4.9622 | 6.12834859 | 12.517126 | 0.000403239 | 0.0047009 | 0.4966571 | 2.7994269 |
| ENSG00000080298 | RFX3 | K09173 | 4.9622001 | 6.12834859 | 12.517369 | 0.000403186 | 0.0047009 | 0.5460614 | 2.8531375 |
| ENSG00000152253 | SPC25 | K11550 | 4.9674685 | 6.132574259 | 11.970764 | 0.000540418 | 0.0059869 | 0.5848214 | 3.1919643 |
| ENSG00000137392 | CLPS | K14460 | 4.993149 | 6.157935271 | 13.501457 | 0.000238378 | 0.0031968 | 0.4375 | 2.5267857 |
| ENSG00000177752 | YIPF7 | - | 4.9965756 | 6.160905237 | 14.610501 | 0.000132176 | 0.0025514 | 0.4428571 | 2.95 |
| ENSG00000139973 | SYT16 | K19328 | 4.9965758 | 6.160905237 | 14.611708 | 0.000132091 | 0.0025514 | 0.5953488 | 2.8108527 |
| ENSG00000161031 | PGLYRP2 | K01446 | 4.9979836 | 6.162107183 | 14.448619 | 0.000144035 | 0.0026567 | 0.3974763 | 2.7492114 |
| ENSG00000117481 | NSUN4 | - | 5.0029778 | 6.166746397 | 15.872837 | 6.77E-05 | 0.0016606 | 0.4539877 | 2.6349693 |
| ENSG00000186431 | FCAR | K06513 | 5.0188295 | 6.183243531 | 13.663518 | 0.000218661 | 0.0029601 | 0.456446 | 3.0174216 |
| ENSG00000089225 | TBX5 | K10179 | 5.0344245 | 6.198570549 | 13.790665 | 0.000204349 | 0.0029001 | 0.488417 | 2.9034749 |
| ENSG00000129757 | CDKN1C | K09993 | 5.036484 | 6.200036653 | 14.830381 | 0.000117625 | 0.002329 | 0.3670886 | 2.4841772 |
| ENSG00000185298 | CCDC137 | - | 5.0364841 | 6.200036653 | 14.830766 | 0.000117601 | 0.002329 | 0.4315068 | 2.9589041 |
| ENSG00000105131 | EPHX3 | - | 5.0464116 | 6.210417044 | 13.847786 | 0.00019823 | 0.0029001 | 0.375 | 3.2138889 |
| ENSG00000141448 | GATA6 | K17897 | 5.0464117 | 6.210417044 | 13.848262 | 0.00019818 | 0.0029001 | 0.3882353 | 2.3394958 |
| ENSG00000117500 | TMED5 | K14825 | 5.0464117 | 6.210417044 | 13.848322 | 0.000198174 | 0.0029001 | 0.5196507 | 3.1310044 |
| ENSG00000124260 | MAGEA10 | - | 5.0724346 | 6.236935797 | 11.788814 | 0.000595877 | 0.0065569 | 0.6151762 | 2.8482385 |
| ENSG00000168488 | ATXN2L | - | 5.0782464 | 6.240563738 | 16.350366 | 5.26E-05 | 0.0014687 | 0.4421149 | 2.6025524 |
| ENSG00000188807 | TMEM201 | - | 5.0861654 | 6.248857806 | 15.198466 | 9.68E-05 | 0.0020977 | 0.3858859 | 2.7912913 |
| ENSG00000159173 | TNNI1 | K10371 | 5.0927109 | 6.255162293 | 15.07729 | 0.000103197 | 0.0022117 | 0.4812834 | 3.0213904 |
| ENSG00000168894 | RNF181 | - | 5.1103643 | 6.272050757 | 16.491771 | 4.89E-05 | 0.0013705 | 0.4882353 | 2.9705882 |
| ENSG00000180900 | SCRIB | K16175 | 5.1231476 | 6.285781734 | 13.026558 | 0.000307104 | 0.0040185 | 0.4839879 | 2.6749245 |
| ENSG00000146648 | EGFR | K04361 | 5.1637221 | 6.326403264 | 12.17389 | 0.000484631 | 0.0054693 | 0.4752066 | 2.8446281 |
| ENSG00000148773 | MKI67 | K17582 | 5.167177 | 6.328710303 | 14.339601 | 0.00015262 | 0.0026567 | 0.536855 | 2.7490786 |
| ENSG00000172116 | CD8B | K06459 | 5.1680895 | 6.328743888 | 16.907624 | 3.92E-05 | 0.0013313 | 0.3786008 | 3.0082305 |
| ENSG00000099840 | IZUMO4 | - | 5.1680899 | 6.328743888 | 16.909433 | 3.92E-05 | 0.0013313 | 0.4310345 | 3.0689655 |
| ENSG00000185963 | BICD2 | K18739 | 5.1708253 | 6.331355628 | 16.752173 | 4.26E-05 | 0.0013566 | 0.6081871 | 2.8701754 |
| ENSG00000007171 | NOS2 | K13241 | 5.1876083 | 6.347514508 | 18.208911 | 1.98E-05 | 0.0009577 | 0.4588031 | 3.0719861 |
| ENSG00000100739 | BDKRB1 | K03915 | 5.2006591 | 6.361204627 | 15.758112 | 7.20E-05 | 0.0016606 | 0.3229462 | 3.3541076 |
| ENSG00000171916 | LGALS9B | K10093 | 5.2190462 | 6.37876066 | 17.127556 | 3.50E-05 | 0.0012144 | 0.3820225 | 3.008427 |
| ENSG00000170298 | LGALS9B | K10093 | 5.2190463 | 6.37876066 | 17.127916 | 3.49E-05 | 0.0012144 | 0.3876404 | 3.0168539 |
| ENSG00000123405 | NFE2 | K09039 | 5.2190472 | 6.37876066 | 17.133045 | 3.49E-05 | 0.0012144 | 0.541555 | 2.8793566 |
| ENSG00000173894 | CBX2 | K11451 | 5.2409175 | 6.401285537 | 14.623446 | 0.000131271 | 0.0025462 | 0.4360902 | 2.5225564 |
| ENSG00000173838 | 10-Mar | K10665 | 5.2563785 | 6.415422573 | 17.27459 | 3.23E-05 | 0.0011836 | 0.5780142 | 2.7789598 |
| ENSG00000064300 | NGFR | K02583 | 5.260151 | 6.421012957 | 12.570403 | 0.000391904 | 0.0047009 | 0.5222482 | 2.4754098 |
| ENSG00000088002 | SULT2B1 | K01015 | 5.2660797 | 6.425015876 | 17.494744 | 2.88E-05 | 0.0011836 | 0.5041096 | 3.0821918 |
| ENSG00000115355 | CCDC88A | - | 5.2853317 | 6.444290131 | 16.053703 | 6.16E-05 | 0.0015414 | 0.6461785 | 2.9609834 |
| ENSG00000137558 | PI15 | - | 5.2853317 | 6.444290131 | 16.053898 | 6.16E-05 | 0.0015414 | 0.4263566 | 3.0077519 |
| ENSG00000132334 | PTPRE | K18033 | 5.2865387 | 6.44557788 | 16.068617 | 6.11E-05 | 0.001536 | 0.4842857 | 3.1585714 |
| ENSG00000104524 | PYCRL | K00286 | 5.2987899 | 6.458153217 | 14.939309 | 0.000111025 | 0.0022981 | 0.3461538 | 2.534965 |
| ENSG00000175329 | ISX | - | 5.3014925 | 6.460194075 | 16.077251 | 6.08E-05 | 0.0015324 | 0.4 | 2.844898 |
| ENSG00000158578 | ALAS2 | K00643 | 5.3120789 | 6.4707477 | 14.620686 | 0.000131464 | 0.0025462 | 0.4139693 | 2.8892675 |
| ENSG00000177679 | SRRM3 | - | 5.319578 | 6.478046694 | 16.317533 | 5.36E-05 | 0.0014907 | 0.4502297 | 2.517611 |
| ENSG00000088386 | SLC15A1 | K14206 | 5.3290261 | 6.486873212 | 17.786709 | 2.47E-05 | 0.0011199 | 0.4053672 | 3.0918079 |
| ENSG00000182253 | SYNM | K10376 | 5.3306011 | 6.489242384 | 14.873049 | 0.000114994 | 0.0022981 | 0.5923323 | 2.7246006 |
| ENSG00000137975 | CLCA2 | K05028 | 5.3306012 | 6.489242384 | 14.873465 | 0.000114969 | 0.0022981 | 0.47614 | 2.9034995 |
| ENSG00000138308 | PLA2G12B | K01047 | 5.3451382 | 6.503685239 | 15.103508 | 0.000101774 | 0.0021853 | 0.4666667 | 2.8461538 |
| ENSG00000081051 | AFP | K16144 | 5.3452289 | 6.502865574 | 17.806792 | 2.45E-05 | 0.0011167 | 0.522508 | 2.9983923 |
| ENSG00000170122 | FOXD4 | K09397 | 5.3452289 | 6.502865574 | 17.807049 | 2.44E-05 | 0.0011167 | 0.3917995 | 2.7084282 |
| ENSG00000184659 | FOXD4L4 | K09397 | 5.345229 | 6.502865574 | 17.807522 | 2.44E-05 | 0.0011167 | 0.3557692 | 2.8389423 |
| ENSG00000111850 | SMIM8 | - | 5.3470985 | 6.504643122 | 17.808434 | 2.44E-05 | 0.0011167 | 0.4536082 | 3.1546392 |
| ENSG00000156802 | ATAD2 | - | 5.3559022 | 6.514145461 | 15.115036 | 0.000101154 | 0.0021761 | 0.5791367 | 2.9431655 |
| ENSG00000171408 | PDE7B | K18436 | 5.3676521 | 6.524910975 | 18.087845 | 2.11E-05 | 0.0009995 | 0.4581673 | 3.0916335 |
| ENSG00000091527 | CDV3 | - | 5.3676524 | 6.524910975 | 18.089348 | 2.11E-05 | 0.0009995 | 0.5542636 | 2.4767442 |
| ENSG00000101336 | HCK | K08893 | 5.3711362 | 6.527899776 | 19.953597 | 7.93E-06 | 0.0007832 | 0.5095057 | 3.0228137 |
| ENSG00000177675 | CD163L1 | K06545 | 5.3790409 | 6.53573018 | 19.960641 | 7.91E-06 | 0.0007832 | 0.4620643 | 2.683527 |
| ENSG00000172361 | CFAP53 | - | 5.3986795 | 6.554970313 | 19.927926 | 8.04E-06 | 0.0007832 | 0.6089494 | 3.1673152 |
| ENSG00000159147 | DONSON | - | 5.416577 | 6.573393483 | 16.601598 | 4.61E-05 | 0.0013705 | 0.4540636 | 2.9310954 |
| ENSG00000130695 | CEP85 | K16766 | 5.4306896 | 6.586977983 | 18.349973 | 1.84E-05 | 0.0009319 | 0.5774278 | 2.8307087 |
| ENSG00000176248 | ANAPC2 | K03349 | 5.4385633 | 6.595257728 | 16.958106 | 3.82E-05 | 0.0013079 | 0.5218978 | 3.0352798 |
| ENSG00000128815 | WDFY4 | - | 5.4405483 | 6.596628456 | 18.449624 | 1.74E-05 | 0.0009319 | 0.4582286 | 2.9519472 |
| ENSG00000123572 | NRK | K16313 | 5.4441306 | 6.600575126 | 16.897045 | 3.95E-05 | 0.0013348 | 0.5385588 | 2.9285714 |
| ENSG00000166819 | PLIN1 | K08768 | 5.4442321 | 6.600254537 | 18.356653 | 1.83E-05 | 0.0009319 | 0.467433 | 2.6666667 |
| ENSG00000157578 | LCA5L | - | 5.4442326 | 6.600254537 | 18.359366 | 1.83E-05 | 0.0009319 | 0.5567164 | 2.941791 |
| ENSG00000146469 | VIP | K05264 | 5.4442329 | 6.600254537 | 18.361135 | 1.83E-05 | 0.0009319 | 0.5529412 | 2.9647059 |
| ENSG00000121207 | LRAT | K00678 | 5.4442332 | 6.600254537 | 18.362853 | 1.83E-05 | 0.0009319 | 0.4304348 | 3.0478261 |
| ENSG00000128708 | HAT1 | K11303 | 5.467928 | 6.623679582 | 18.733056 | 1.50E-05 | 0.0009319 | 0.5536993 | 3.3412888 |
| ENSG00000177143 | CETN1 | K16465 | 5.4943055 | 6.649730311 | 18.784134 | 1.46E-05 | 0.0009319 | 0.6337209 | 2.9360465 |
| ENSG00000140368 | PSTPIP1 | K12804 | 5.4963178 | 6.651984414 | 17.099349 | 3.55E-05 | 0.0012252 | 0.5251799 | 2.8848921 |
| ENSG00000154719 | MRPL39 | K17420 | 5.5063542 | 6.662050246 | 17.101745 | 3.54E-05 | 0.0012252 | 0.4589235 | 3.1614731 |
| ENSG00000161011 | SQSTM1 | K14381 | 5.5271448 | 6.68156418 | 20.724986 | 5.30E-06 | 0.000676 | 0.5113636 | 2.6772727 |
| ENSG00000133808 | MICALCL | - | 5.5394032 | 6.694418208 | 17.351223 | 3.11E-05 | 0.0011836 | 0.533813 | 2.8273381 |
| ENSG00000166508 | MCM7 | K02210 | 5.5400324 | 6.695186765 | 17.497615 | 2.88E-05 | 0.0011836 | 0.5076495 | 2.945758 |
| ENSG00000131979 | GCH1 | K01495 | 5.5400326 | 6.695186765 | 17.498805 | 2.87E-05 | 0.0011836 | 0.452 | 2.888 |
| ENSG00000167613 | LAIR1 | K06725 | 5.5455197 | 6.699666059 | 21.139317 | 4.27E-06 | 0.000676 | 0.533101 | 2.7735192 |
| ENSG00000079435 | LIPE | K07188 | 5.5754929 | 6.730463649 | 14.412762 | 0.000146804 | 0.0026567 | 0.4684015 | 2.7481413 |
| ENSG00000132855 | ANGPTL3 | - | 5.6128667 | 6.766826362 | 17.647294 | 2.66E-05 | 0.0011592 | 0.5782609 | 3.1956522 |
| ENSG00000172404 | DNAJB7 | K09513 | 5.6186836 | 6.771832973 | 21.815965 | 3.00E-06 | 0.0005722 | 0.6375405 | 3.0614887 |
| ENSG00000047457 | CP | K13624 | 5.6218538 | 6.775355378 | 19.485057 | 1.01E-05 | 0.0007832 | 0.5464789 | 3.1492958 |
| ENSG00000106462 | EZH2 | K11430 | 5.6310608 | 6.784575441 | 19.485078 | 1.01E-05 | 0.0007832 | 0.5472703 | 2.954727 |
| ENSG00000151650 | VENTX | - | 5.6707403 | 6.82358292 | 19.866766 | 8.30E-06 | 0.0007832 | 0.3837209 | 2.6511628 |
| ENSG00000167080 | B4GALNT2 | K09655 | 5.6744964 | 6.828230885 | 16.586018 | 4.65E-05 | 0.0013705 | 0.409894 | 3.0724382 |
| ENSG00000162366 | PDZK1IP1 | - | 5.6978878 | 6.850771763 | 18.14132 | 2.05E-05 | 0.0009831 | 0.4122807 | 2.7631579 |
| ENSG00000156239 | N6AMT1 | K19589 | 5.6992238 | 6.851249258 | 22.130927 | 2.55E-06 | 0.0005309 | 0.4252336 | 2.8037383 |
| ENSG00000108395 | TRIM37 | K10608 | 5.7062435 | 6.858269774 | 22.396934 | 2.22E-06 | 0.0004946 | 0.5881743 | 2.7842324 |
| ENSG00000096717 | SIRT1 | K11411 | 5.7247301 | 6.87644318 | 22.085619 | 2.61E-06 | 0.0005339 | 0.562249 | 2.7456493 |
| ENSG00000149474 | CSRP2BP | - | 5.7490751 | 6.900486207 | 22.41207 | 2.20E-06 | 0.0004946 | 0.5255754 | 3.0319693 |
| ENSG00000139344 | AMDHD1 | K01468 | 5.7490753 | 6.900486207 | 22.413029 | 2.20E-06 | 0.0004946 | 0.4248826 | 2.814554 |
| ENSG00000170312 | CDK1 | K02087 | 5.7797469 | 6.931130939 | 20.029237 | 7.63E-06 | 0.0007832 | 0.4781145 | 3.2053872 |
| ENSG00000164105 | SAP30 | K19202 | 5.8069388 | 6.957628053 | 22.828351 | 1.77E-06 | 0.0004946 | 0.4318182 | 2.5590909 |
| ENSG00000165113 | GKAP1 | - | 5.8069391 | 6.957628053 | 22.830677 | 1.77E-06 | 0.0004946 | 0.6202186 | 2.9098361 |
| ENSG00000088053 | GP6 | K06264 | 5.8202734 | 6.970811729 | 22.943543 | 1.67E-06 | 0.0004946 | 0.4 | 2.8080645 |
| ENSG00000166869 | CHP2 | K17611 | 5.8257624 | 6.976610292 | 20.502824 | 5.95E-06 | 0.0007051 | 0.5357143 | 3 |
| ENSG00000184937 | WT1 | K09234 | 5.8418023 | 7.486364716 | 19.033121 | 1.28E-05 | 0.0009319 | 0.4294004 | 2.7176015 |
| ENSG00000111530 | CAND1 | K17263 | 5.8606777 | 7.010742348 | 22.964186 | 1.65E-06 | 0.0004946 | 0.495935 | 2.9121951 |
| ENSG00000180745 | CLRN3 | - | 5.8871782 | 7.036984606 | 23.662249 | 1.15E-06 | 0.0004702 | 0.4469027 | 3.1814159 |
| ENSG00000183549 | ACSM5 | K01896 | 5.9000941 | 7.051004286 | 17.073875 | 3.60E-05 | 0.001238 | 0.4404145 | 2.9810017 |
| ENSG00000131747 | TOP2A | K03164 | 5.9154906 | 7.064979268 | 23.714668 | 1.12E-06 | 0.0004702 | 0.5395167 | 3.0431091 |
| ENSG00000154099 | DNAAF1 | K19750 | 5.9196875 | 7.069076731 | 23.492899 | 1.25E-06 | 0.0004946 | 0.6041379 | 2.7627586 |
| ENSG00000163687 | DNASE1L3 | K11995 | 5.9858067 | 7.135040919 | 21.519879 | 3.50E-06 | 0.0006084 | 0.5016393 | 3.2131148 |
| ENSG00000080618 | CPB2 | K01300 | 6.0358592 | 7.184020189 | 24.03056 | 9.48E-07 | 0.0004585 | 0.4751773 | 3.177305 |
| ENSG00000123146 | ADGRE5 | K08446 | 6.0358593 | 7.184020189 | 24.031139 | 9.48E-07 | 0.0004585 | 0.445509 | 2.8419162 |
| ENSG00000005102 | MEOX1 | K09322 | 6.0998066 | 7.247339091 | 24.645557 | 6.89E-07 | 0.0004389 | 0.515748 | 2.7559055 |
| ENSG00000115257 | PCSK4 | K08671 | 6.1156674 | 7.263114953 | 24.92007 | 5.98E-07 | 0.0004282 | 0.4172185 | 2.8145695 |
| ENSG00000173464 | RNASE11 | K16633 | 6.147184 | 7.294346069 | 25.171808 | 5.24E-07 | 0.0004064 | 0.5376884 | 2.8592965 |
| ENSG00000160856 | FCRL3 | K06727 | 6.1539063 | 7.300962972 | 25.145278 | 5.32E-07 | 0.0004064 | 0.490566 | 2.8342318 |
| ENSG00000175311 | ANKS4B | K21414 | 6.1904697 | 7.337349481 | 24.738684 | 6.57E-07 | 0.0004389 | 0.5947242 | 2.822542 |
| ENSG00000164542 | KIAA0895 | - | 6.2340797 | 7.380494931 | 25.509478 | 4.40E-07 | 0.0003883 | 0.45 | 3.1865385 |
| ENSG00000183476 | SH2D7 | - | 6.2519101 | 7.398089106 | 25.681798 | 4.03E-07 | 0.0003847 | 0.5121951 | 2.8337029 |
| ENSG00000173585 | CCR9 | K04184 | 6.2593773 | 7.405581745 | 25.724379 | 3.94E-07 | 0.0003847 | 0.398374 | 3.2276423 |
| ENSG00000119655 | NPC2 | K13443 | 6.2923316 | 7.43817842 | 25.983379 | 3.44E-07 | 0.0003847 | 0.4886878 | 2.7149321 |
| ENSG00000177291 | GJD4 | K07630 | 6.3711472 | 7.516372211 | 26.374752 | 2.81E-07 | 0.0003847 | 0.3675676 | 2.7756757 |
| ENSG00000189132 | FAM47B | - | 6.4249995 | 7.569849306 | 25.798208 | 3.79E-07 | 0.0003847 | 0.4914729 | 3.048062 |
| ENSG00000188523 | CFAP77 | - | 6.5387294 | 7.68283277 | 26.489918 | 2.65E-07 | 0.0003847 | 0.4125 | 3.0375 |
| ENSG00000136574 | GATA4 | K09183 | 6.5454084 | 7.689422708 | 26.725227 | 2.35E-07 | 0.0003847 | 0.3860045 | 2.3273138 |
| ENSG00000165490 | DDIAS | - | 6.6340492 | 7.777999222 | 23.345291 | 1.35E-06 | 0.0004946 | 0.5460922 | 2.8957916 |
| ENSG00000030419 | IKZF2 | K09220 | 6.6431991 | 7.786589521 | 26.884316 | 2.16E-07 | 0.0003847 | 0.5338346 | 2.8402256 |
| ENSG00000178772 | CPN2 | K13023 | 6.6990594 | 7.842103504 | 26.92325 | 2.12E-07 | 0.0003847 | 0.4422018 | 3.0055046 |
| ENSG00000137948 | BRDT | K11724 | 6.7488801 | 7.891646235 | 26.959863 | 2.08E-07 | 0.0003847 | 0.575184 | 2.9463722 |
| ENSG00000162415 | ZSWIM5 | - | 7.0355968 | 8.17687418 | 27.302856 | 1.74E-07 | 0.0003847 | 0.4447257 | 2.8489451 |
| ENSG00000162949 | CAPN13 | K08581 | -6.3079689 | 6.36817115 | 26.3666 | 2.82E-07 | 0.0003847 | 0.4753363 | 3.1210762 |
| ENSG00000116580 | GON4L | - | -6.0164675 | 6.088644623 | 23.123443 | 1.52E-06 | 0.0004946 | 0.5845605 | 2.799643 |
| ENSG00000163374 | YY1AP1 | - | -6.0164674 | 6.088644623 | 23.123193 | 1.52E-06 | 0.0004946 | 0.4358108 | 2.8704955 |
| ENSG00000151692 | RNF144A | K11975 | -5.9383323 | 6.013702709 | 24.415971 | 7.76E-07 | 0.0004498 | 0.4246575 | 2.9280822 |
| ENSG00000072518 | MARK2 | K08798 | -5.9299474 | 6.005734125 | 24.39514 | 7.85E-07 | 0.0004498 | 0.4923858 | 2.8591371 |
| ENSG00000079215 | SLC1A3 | K05614 | -5.8839925 | 5.962053295 | 22.586566 | 2.01E-06 | 0.0004946 | 0.396679 | 2.9649446 |
| ENSG00000114353 | GNAI2 | K04630 | -5.8194773 | 5.900769122 | 24.035724 | 9.46E-07 | 0.0004585 | 0.5492958 | 3.0197183 |
| ENSG00000075391 | RASAL2 | K17633 | -5.8103693 | 5.892156113 | 24.007371 | 9.60E-07 | 0.0004585 | 0.546875 | 2.8921875 |
| ENSG00000156642 | NPTN | - | -5.810025 | 5.89235346 | 22.393633 | 2.22E-06 | 0.0004946 | 0.5100503 | 2.8844221 |
| ENSG00000169258 | GPRIN1 | - | -5.8011681 | 5.883980304 | 22.375355 | 2.24E-06 | 0.0004946 | 0.4613095 | 2.328373 |
| ENSG00000074317 | SNCB | - | -5.8011679 | 5.883980304 | 22.374624 | 2.24E-06 | 0.0004946 | 0.5970149 | 2.6492537 |
| ENSG00000129484 | PARP2 | K10798 | -5.7813946 | 5.864651421 | 22.260698 | 2.38E-06 | 0.000515 | 0.4922813 | 2.9828473 |
| ENSG00000175581 | MRPL48 | K17429 | -5.7578086 | 5.841849224 | 23.800759 | 1.07E-06 | 0.0004702 | 0.4858491 | 3.0801887 |
| ENSG00000104375 | STK3 | K04412 | -5.7578084 | 5.841849224 | 23.800241 | 1.07E-06 | 0.0004702 | 0.5722543 | 3.0154143 |
| ENSG00000067445 | TRO | - | -5.7439403 | 5.829292134 | 22.145261 | 2.53E-06 | 0.0005309 | 0.4451433 | 2.3619846 |
| ENSG00000066855 | MTFR1 | - | -5.6583678 | 5.748916972 | 20.483514 | 6.01E-06 | 0.0007051 | 0.4744745 | 2.8888889 |
| ENSG00000100372 | SLC25A17 | K13354 | -5.6583676 | 5.748916972 | 20.482805 | 6.02E-06 | 0.0007051 | 0.3485342 | 3.1758958 |
| ENSG00000138107 | ACTR1A | K16575 | -5.6520522 | 5.741994433 | 21.487257 | 3.56E-06 | 0.0006096 | 0.481383 | 3.1037234 |
| ENSG00000184363 | PKP3 | - | -5.6445408 | 5.734486952 | 22.682824 | 1.91E-06 | 0.0004946 | 0.4604768 | 2.7478043 |
| ENSG00000152818 | UTRN | - | -5.6445407 | 5.734486952 | 22.682398 | 1.91E-06 | 0.0004946 | 0.5901544 | 3.011069 |
| ENSG00000078369 | GNB1 | K04536 | -5.6258554 | 5.717384832 | 21.384017 | 3.76E-06 | 0.0006338 | 0.5029412 | 2.7176471 |
| ENSG00000107672 | NSMCE4A | - | -5.619383 | 5.711372585 | 22.996454 | 1.62E-06 | 0.0004946 | 0.5844156 | 2.9844156 |
| ENSG00000169231 | THBS3 | K04659 | -5.6193822 | 5.711372585 | 22.994079 | 1.63E-06 | 0.0004946 | 0.5491632 | 2.6474895 |
| ENSG00000105088 | OLFM2 | - | -5.6175339 | 5.710598805 | 20.332984 | 6.51E-06 | 0.0007085 | 0.4823789 | 3.0110132 |
| ENSG00000135363 | LMO2 | K15612 | -5.6089206 | 5.701556593 | 22.974508 | 1.64E-06 | 0.0004946 | 0.4229075 | 2.7312775 |
| ENSG00000171132 | PRKCE | K18050 | -5.6061908 | 5.699960515 | 20.303154 | 6.61E-06 | 0.0007085 | 0.4748982 | 3.0230665 |
| ENSG00000168398 | BDKRB2 | K03916 | -5.6061905 | 5.699960515 | 20.302467 | 6.61E-06 | 0.0007085 | 0.3938619 | 3.1534527 |
| ENSG00000077063 | CTTNBP2 | - | -5.6060514 | 5.69886516 | 22.966597 | 1.65E-06 | 0.0004946 | 0.4912808 | 2.7047505 |
| ENSG00000185518 | SV2B | K06258 | -5.604859 | 5.69722032 | 22.547832 | 2.05E-06 | 0.0004946 | 0.4465593 | 3.147877 |
| ENSG00000185742 | C11orf87 | - | -5.6013809 | 5.694939355 | 21.668172 | 3.24E-06 | 0.0005722 | 0.3654822 | 2.5076142 |
| ENSG00000154146 | NRGN | - | -5.6013804 | 5.694939355 | 21.666755 | 3.24E-06 | 0.0005722 | 0.3589744 | 2.025641 |
| ENSG00000120008 | WDR11 | - | -5.5807697 | 5.67491107 | 21.259681 | 4.01E-06 | 0.000657 | 0.4730392 | 2.9599673 |
| ENSG00000166145 | SPINT1 | K15619 | -5.5807697 | 5.67491107 | 21.259579 | 4.01E-06 | 0.000657 | 0.4669187 | 2.826087 |
| ENSG00000003436 | TFPI | K03909 | -5.545306 | 5.641125602 | 22.717291 | 1.88E-06 | 0.0004946 | 0.4901316 | 3.0592105 |
| ENSG00000103653 | CSK | K05728 | -5.5453059 | 5.641125602 | 22.717095 | 1.88E-06 | 0.0004946 | 0.4666667 | 3.0355556 |
| ENSG00000105376 | ICAM5 | K06769 | -5.5285903 | 5.626074128 | 21.107942 | 4.34E-06 | 0.000676 | 0.4155844 | 2.547619 |
| ENSG00000141642 | ELAC1 | K00784 | -5.5133112 | 5.611373735 | 19.666981 | 9.22E-06 | 0.0007832 | 0.4352617 | 2.9283747 |
| ENSG00000172339 | ALG14 | K07441 | -5.5133104 | 5.611373735 | 19.664541 | 9.23E-06 | 0.0007832 | 0.3703704 | 3.0833333 |
| ENSG00000139292 | LGR5 | K04308 | -5.4791723 | 5.578973113 | 20.906031 | 4.82E-06 | 0.000676 | 0.4266814 | 2.9867696 |
| ENSG00000108684 | ASIC2 | K04828 | -5.4617692 | 5.563257577 | 19.517526 | 9.97E-06 | 0.0007832 | 0.4404973 | 2.9698046 |
| ENSG00000158869 | FCER1G | K07983 | -5.461769 | 5.563257577 | 19.516966 | 9.97E-06 | 0.0007832 | 0.3839286 | 2.8839286 |
| ENSG00000072415 | MPP5 | K06091 | -5.4617689 | 5.563257577 | 19.51661 | 9.97E-06 | 0.0007832 | 0.5555556 | 3.0148148 |
| ENSG00000109832 | DDX25 | K18656 | -5.3887986 | 5.495445539 | 19.430782 | 1.04E-05 | 0.0007832 | 0.4927536 | 3.0124224 |
| ENSG00000139567 | ACVRL1 | K13594 | -5.3805017 | 5.487749488 | 20.732092 | 5.28E-06 | 0.000676 | 0.4255319 | 2.8916828 |
| ENSG00000156535 | CD109 | K06530 | -5.3791217 | 5.485706403 | 20.36131 | 6.41E-06 | 0.0007085 | 0.5190311 | 3.0221453 |
| ENSG00000157734 | SNX22 | K17941 | -5.3791216 | 5.485706403 | 20.361153 | 6.41E-06 | 0.0007085 | 0.4041451 | 3.1398964 |
| ENSG00000158806 | NPM2 | K11277 | -5.371944 | 5.479815639 | 20.710964 | 5.34E-06 | 0.000676 | 0.6168224 | 2.8691589 |
| ENSG00000116885 | OSCP1 | - | -5.3719437 | 5.479815639 | 20.71021 | 5.34E-06 | 0.000676 | 0.5115681 | 3.1079692 |
| ENSG00000140280 | LYSMD2 | - | -5.3685639 | 5.476680751 | 20.703477 | 5.36E-06 | 0.000676 | 0.5627907 | 2.7302326 |
| ENSG00000092445 | TYRO3 | K05116 | -5.3685638 | 5.476680751 | 20.703249 | 5.36E-06 | 0.000676 | 0.4269663 | 2.8325843 |
| ENSG00000152413 | HOMER1 | K15010 | -5.3685637 | 5.476680751 | 20.703145 | 5.36E-06 | 0.000676 | 0.6327684 | 2.940678 |
| ENSG00000179841 | AKAP5 | K16522 | -5.3685635 | 5.476680751 | 20.702454 | 5.36E-06 | 0.000676 | 0.6533958 | 2.7002342 |
| ENSG00000106089 | STX1A | K04560 | -5.3671682 | 5.474614132 | 20.32476 | 6.53E-06 | 0.0007085 | 0.6215278 | 2.9201389 |
| ENSG00000132639 | SNAP25 | K18211 | -5.3671681 | 5.474614132 | 20.324373 | 6.54E-06 | 0.0007085 | 0.6359223 | 2.6699029 |
| ENSG00000073150 | PANX2 | K20857 | -5.367168 | 5.474614132 | 20.3242 | 6.54E-06 | 0.0007085 | 0.4062038 | 2.957164 |
| ENSG00000135424 | ITGA7 | K06583 | -5.3161508 | 5.426993555 | 20.498449 | 5.97E-06 | 0.0007051 | 0.442845 | 2.8408129 |
| ENSG00000132781 | MUTYH | K03575 | -5.3161507 | 5.426993555 | 20.498008 | 5.97E-06 | 0.0007051 | 0.431694 | 2.7814208 |
| ENSG00000164197 | RNF180 | K15708 | -5.3092312 | 5.420589043 | 20.480365 | 6.02E-06 | 0.0007051 | 0.4763514 | 3.0861486 |
| ENSG00000132361 | CLUH | K03255 | -5.3092309 | 5.420589043 | 20.479657 | 6.03E-06 | 0.0007051 | 0.4935065 | 2.9518717 |
| ENSG00000177946 | CENPBD1 | - | -5.3059174 | 5.417033842 | 21.74885 | 3.11E-06 | 0.0005722 | 0.5294118 | 2.8877005 |
| ENSG00000166548 | TK2 | K00857 | -5.3059169 | 5.417033842 | 21.747567 | 3.11E-06 | 0.0005722 | 0.4201954 | 3.0846906 |
| ENSG00000065308 | TRAM2 | - | -5.2929045 | 5.40500248 | 21.713833 | 3.17E-06 | 0.0005722 | 0.3675676 | 3.4783784 |
| ENSG00000183760 | ACP7 | - | -5.2929042 | 5.40500248 | 21.71289 | 3.17E-06 | 0.0005722 | 0.4086758 | 3.2009132 |
| ENSG00000136928 | GABBR2 | K04615 | -5.2896609 | 5.403073988 | 19.057273 | 1.27E-05 | 0.0009319 | 0.4431456 | 3.0478215 |
| ENSG00000117408 | IPO13 | - | -5.2896606 | 5.403073988 | 19.056553 | 1.27E-05 | 0.0009319 | 0.463136 | 3.066459 |
| ENSG00000102003 | SYP | - | -5.2896606 | 5.403073988 | 19.056417 | 1.27E-05 | 0.0009319 | 0.4345048 | 2.9169329 |
| ENSG00000090487 | SPG21 | K19367 | -5.2893339 | 5.401700127 | 21.70757 | 3.18E-06 | 0.0005722 | 0.461039 | 3.1428571 |
| ENSG00000186625 | KATNA1 | K07767 | -5.2893333 | 5.401700127 | 21.705782 | 3.18E-06 | 0.0005722 | 0.5336049 | 2.9796334 |
| ENSG00000175920 | DOK7 | - | -5.2835902 | 5.396891687 | 20.413839 | 6.24E-06 | 0.0007085 | 0.4246032 | 2.5972222 |
| ENSG00000099998 | GGT5 | K18592 | -5.2617318 | 5.376095528 | 18.659149 | 1.56E-05 | 0.0009319 | 0.3867121 | 2.7086882 |
| ENSG00000157600 | TMEM164 | - | -5.2617317 | 5.376095528 | 18.658943 | 1.56E-05 | 0.0009319 | 0.3434343 | 3.2323232 |
| ENSG00000024422 | EHD2 | K12469 | -5.2617316 | 5.376095528 | 18.658789 | 1.56E-05 | 0.0009319 | 0.4567219 | 3.053407 |
| ENSG00000166143 | PPP1R14D | K17557 | -5.2617315 | 5.376095528 | 18.658368 | 1.56E-05 | 0.0009319 | 0.445 | 2.885 |
| ENSG00000143772 | ITPKB | K00911 | -5.2275593 | 5.344048671 | 19.910106 | 8.12E-06 | 0.0007832 | 0.4788584 | 2.667019 |
| ENSG00000167371 | PRRT2 | - | -5.2098164 | 5.328222355 | 18.534611 | 1.67E-05 | 0.0009319 | 0.4593909 | 2.4162437 |
| ENSG00000108381 | ASPA | K01437 | -5.2077675 | 5.326341776 | 18.529899 | 1.67E-05 | 0.0009319 | 0.4760383 | 3.1341853 |
| ENSG00000145012 | LPP | K16676 | -5.1708587 | 5.292108864 | 18.446501 | 1.75E-05 | 0.0009319 | 0.4379085 | 2.7336601 |
| ENSG00000082438 | COBLL1 | - | -5.1708587 | 5.292108864 | 18.446423 | 1.75E-05 | 0.0009319 | 0.5563666 | 2.729927 |
| ENSG00000175899 | A2M | K03910 | -5.1708585 | 5.292108864 | 18.445928 | 1.75E-05 | 0.0009319 | 0.4959294 | 2.9518318 |
| ENSG00000103005 | USB1 | - | -5.1265819 | 5.250944471 | 19.688294 | 9.12E-06 | 0.0007832 | 0.4867925 | 3.090566 |
| ENSG00000146278 | PNRC1 | K18774 | -5.1265817 | 5.250944471 | 19.687636 | 9.12E-06 | 0.0007832 | 0.3608563 | 2.7920489 |
| ENSG00000156463 | SH3RF2 | K12171 | -5.1265816 | 5.250944471 | 19.687435 | 9.12E-06 | 0.0007832 | 0.4279835 | 2.7626886 |
| ENSG00000159792 | PSKH1 | K08808 | -5.1068932 | 5.234615388 | 18.618392 | 1.60E-05 | 0.0009319 | 0.4528302 | 2.9834906 |
| ENSG00000160062 | ZBTB8A | K10495 | -5.1039432 | 5.229690311 | 20.919225 | 4.79E-06 | 0.000676 | 0.5782313 | 2.9115646 |
| ENSG00000174606 | ANGEL2 | K18729 | -5.1039431 | 5.229690311 | 20.919001 | 4.79E-06 | 0.000676 | 0.4742647 | 3.0882353 |
| ENSG00000138356 | AOX1 | K00157 | -5.1039427 | 5.229690311 | 20.917951 | 4.79E-06 | 0.000676 | 0.4349776 | 2.9058296 |
| ENSG00000085998 | POMGNT1 | K09666 | -5.1039425 | 5.229690311 | 20.917588 | 4.79E-06 | 0.000676 | 0.4612299 | 3.0534759 |
| ENSG00000091592 | NLRP1 | K12798 | -5.1022065 | 5.229796305 | 19.717145 | 8.98E-06 | 0.0007832 | 0.4582485 | 3.0142566 |
| ENSG00000164683 | HEY1 | K09091 | -5.0988968 | 5.227310075 | 18.603476 | 1.61E-05 | 0.0009319 | 0.4253247 | 2.7175325 |
| ENSG00000131174 | COX7B | K02271 | -5.0988967 | 5.227310075 | 18.603391 | 1.61E-05 | 0.0009319 | 0.4125 | 3.075 |
| ENSG00000127585 | FBXL16 | K10282 | -5.0988967 | 5.227310075 | 18.603288 | 1.61E-05 | 0.0009319 | 0.4070981 | 2.7891441 |
| ENSG00000165501 | LRR1 | K10348 | -5.0988966 | 5.227310075 | 18.603158 | 1.61E-05 | 0.0009319 | 0.4251208 | 3.0434783 |
| ENSG00000177380 | PPFIA3 | - | -5.0988966 | 5.227310075 | 18.603074 | 1.61E-05 | 0.0009319 | 0.5544389 | 2.7897822 |
| ENSG00000184979 | USP18 | K11846 | -5.0972146 | 5.224621215 | 18.270696 | 1.92E-05 | 0.0009319 | 0.4784946 | 3.094086 |
| ENSG00000166171 | DPCD | K20800 | -5.0972145 | 5.224621215 | 18.270613 | 1.92E-05 | 0.0009319 | 0.4926108 | 3.0788177 |
| ENSG00000134830 | C5AR2 | K04171 | -5.0972145 | 5.224621215 | 18.27051 | 1.92E-05 | 0.0009319 | 0.2908012 | 2.8338279 |
| ENSG00000011198 | ABHD5 | K13699 | -5.0972145 | 5.224621215 | 18.270415 | 1.92E-05 | 0.0009319 | 0.4441261 | 3.025788 |
| ENSG00000100321 | SYNGR1 | - | -5.0972141 | 5.224621215 | 18.269503 | 1.92E-05 | 0.0009319 | 0.4549356 | 2.9871245 |
| ENSG00000022355 | GABRA1 | K05175 | -5.097214 | 5.224621215 | 18.269254 | 1.92E-05 | 0.0009319 | 0.4539474 | 3.1425439 |
| ENSG00000014641 | MDH1 | K00025 | -5.0972139 | 5.224621215 | 18.269015 | 1.92E-05 | 0.0009319 | 0.4602273 | 2.8948864 |
| ENSG00000152495 | CAMK4 | K05869 | -5.087727 | 5.216575832 | 19.696118 | 9.08E-06 | 0.0007832 | 0.4778013 | 2.8879493 |
| ENSG00000188636 | LDOC1L | - | -5.0877269 | 5.216575832 | 19.695926 | 9.08E-06 | 0.0007832 | 0.4142259 | 2.7238494 |
| ENSG00000047346 | FAM214A | - | -5.0877266 | 5.216575832 | 19.695151 | 9.08E-06 | 0.0007832 | 0.5586334 | 2.9261311 |
| ENSG00000164010 | ERMAP | K06712 | -5.0270515 | 5.159699936 | 19.483803 | 1.01E-05 | 0.0007832 | 0.4126316 | 2.96 |
| ENSG00000132196 | HSD17B7 | K13373 | -5.0270514 | 5.159699936 | 19.483489 | 1.01E-05 | 0.0007832 | 0.4486804 | 3.0322581 |
| ENSG00000135917 | SLC19A3 | K14610 | -5.0270513 | 5.159699936 | 19.483297 | 1.01E-05 | 0.0007832 | 0.4274194 | 3.2237903 |
| ENSG00000136895 | GARNL3 | - | -5.0229028 | 5.155920961 | 19.477328 | 1.02E-05 | 0.0007832 | 0.4876604 | 2.9318855 |
| ENSG00000148384 | INPP5E | K20278 | -5.0229025 | 5.155920961 | 19.476637 | 1.02E-05 | 0.0007832 | 0.4565217 | 2.7872671 |
| ENSG00000152520 | PAN3 | K12572 | -5.0229023 | 5.155920961 | 19.476174 | 1.02E-05 | 0.0007832 | 0.4261556 | 2.7688839 |
| ENSG00000173085 | COQ2 | K06125 | -5.0229021 | 5.155920961 | 19.475751 | 1.02E-05 | 0.0007832 | 0.3230404 | 2.8812352 |
| ENSG00000148908 | RGS10 | K16449 | -5.0112623 | 5.145332745 | 19.458036 | 1.03E-05 | 0.0007832 | 0.6022099 | 3.1436464 |
| ENSG00000152954 | NRSN1 | - | -5.0027165 | 5.137558543 | 19.445355 | 1.04E-05 | 0.0007832 | 0.425641 | 2.9692308 |
| ENSG00000167186 | COQ7 | K06134 | -5.0027164 | 5.137558543 | 19.445181 | 1.04E-05 | 0.0007832 | 0.4147465 | 2.9170507 |
| ENSG00000010072 | SPRTN | - | -4.998572 | 5.133226625 | 20.752812 | 5.23E-06 | 0.000676 | 0.5337423 | 2.9120654 |
| ENSG00000106236 | NPTX2 | - | -4.9985718 | 5.133226625 | 20.752241 | 5.23E-06 | 0.000676 | 0.4547564 | 2.8027842 |
| ENSG00000107562 | CXCL12 | K10031 | -4.9985703 | 5.133226625 | 20.748818 | 5.24E-06 | 0.000676 | 0.2928571 | 2.95 |
| ENSG00000184368 | MAP7D2 | - | -4.9985692 | 5.133226625 | 20.746151 | 5.24E-06 | 0.000676 | 0.5562743 | 2.7658473 |
| ENSG00000183454 | GRIN2A | K05209 | -4.998569 | 5.133226625 | 20.745645 | 5.25E-06 | 0.000676 | 0.5075137 | 2.9959016 |
| ENSG00000178363 | CALML3 | K02183 | -4.9985689 | 5.133226625 | 20.745396 | 5.25E-06 | 0.000676 | 0.6711409 | 2.8187919 |
| ENSG00000100554 | ATP6V1D | K02149 | -4.9872009 | 5.123461227 | 19.430458 | 1.04E-05 | 0.0007832 | 0.5020243 | 3.0769231 |
| ENSG00000112137 | PHACTR1 | - | -4.9872006 | 5.123461227 | 19.429783 | 1.04E-05 | 0.0007832 | 0.562069 | 2.9327586 |
| ENSG00000132718 | SYT11 | K19911 | -4.9872003 | 5.123461227 | 19.429108 | 1.04E-05 | 0.0007832 | 0.4547564 | 3.0069606 |
| ENSG00000182831 | C16orf72 | - | -4.9872 | 5.123461227 | 19.428355 | 1.04E-05 | 0.0007832 | 0.5236364 | 2.8327273 |
| ENSG00000135709 | KIAA0513 | - | -4.9871999 | 5.123461227 | 19.428209 | 1.04E-05 | 0.0007832 | 0.6131387 | 2.9294404 |
| ENSG00000143847 | PPFIA4 | - | -4.9871998 | 5.123461227 | 19.427935 | 1.04E-05 | 0.0007832 | 0.5556492 | 2.8499157 |
| ENSG00000182670 | TTC3 | K15712 | -4.9871998 | 5.123461227 | 19.427794 | 1.04E-05 | 0.0007832 | 0.5279012 | 3.0182716 |
| ENSG00000172292 | CERS6 | K04710 | -4.9871995 | 5.123461227 | 19.427234 | 1.05E-05 | 0.0007832 | 0.4285714 | 3.3877551 |
| ENSG00000174059 | CD34 | K06474 | -4.9735389 | 5.11039007 | 16.761041 | 4.24E-05 | 0.0013566 | 0.5350649 | 2.5324675 |
| ENSG00000177469 | PTRF | K19387 | -4.9735389 | 5.11039007 | 16.760984 | 4.24E-05 | 0.0013566 | 0.5846154 | 2.8179487 |
| ENSG00000170955 | PRKCDBP | - | -4.9735389 | 5.11039007 | 16.760973 | 4.24E-05 | 0.0013566 | 0.4505119 | 2.5665529 |
| ENSG00000180537 | RNF182 | K11983 | -4.9735386 | 5.11039007 | 16.760264 | 4.24E-05 | 0.0013566 | 0.4493927 | 2.9392713 |
| ENSG00000168497 | SDPR | - | -4.9735385 | 5.11039007 | 16.759964 | 4.24E-05 | 0.0013566 | 0.6494118 | 2.7152941 |
| ENSG00000164181 | ELOVL7 | K10250 | -4.9735383 | 5.11039007 | 16.759544 | 4.24E-05 | 0.0013566 | 0.366548 | 3.6156584 |
| ENSG00000186994 | KANK3 | - | -4.9735383 | 5.11039007 | 16.759527 | 4.24E-05 | 0.0013566 | 0.4845238 | 2.4702381 |
| ENSG00000148671 | ADIRF | K21408 | -4.9735383 | 5.11039007 | 16.759378 | 4.24E-05 | 0.0013566 | 0.5394737 | 2.3815789 |
| ENSG00000142910 | TINAGL1 | - | -4.9735378 | 5.11039007 | 16.758374 | 4.25E-05 | 0.0013566 | 0.4154176 | 2.875803 |
| ENSG00000138131 | LOXL4 | K00280 | -4.9735378 | 5.11039007 | 16.758153 | 4.25E-05 | 0.0013566 | 0.4378307 | 2.8465608 |
| ENSG00000140157 | NIPA2 | - | -4.9735376 | 5.11039007 | 16.757737 | 4.25E-05 | 0.0013566 | 0.3583333 | 2.9944444 |
| ENSG00000118777 | ABCG2 | K05681blood | -4.9735375 | 5.11039007 | 16.757603 | 4.25E-05 | 0.0013566 | 0.4152672 | 3.0122137 |
| ENSG00000136877 | FPGS | K01930 | -4.9735375 | 5.11039007 | 16.757595 | 4.25E-05 | 0.0013566 | 0.4020443 | 2.890971 |
| ENSG00000136098 | NEK3 | K20873 | -4.9735375 | 5.11039007 | 16.757447 | 4.25E-05 | 0.0013566 | 0.5256917 | 3.0158103 |
| ENSG00000117394 | SLC2A1 | K07299member | -4.9735372 | 5.11039007 | 16.756747 | 4.25E-05 | 0.0013566 | 0.347561 | 3.0752033 |
| ENSG00000111181 | SLC6A12 | K05039 | -4.9735368 | 5.11039007 | 16.755892 | 4.25E-05 | 0.0013566 | 0.3745928 | 3.267101 |
| ENSG00000110799 | VWF | K03900 | -4.9735365 | 5.11039007 | 16.755135 | 4.25E-05 | 0.0013566 | 0.4784927 | 2.7237824 |
| ENSG00000105971 | CAV2 | K12958 | -4.9735361 | 5.11039007 | 16.754023 | 4.26E-05 | 0.0013566 | 0.462963 | 3.1419753 |
| ENSG00000104938 | CLEC4M | K06563 | -4.9735356 | 5.11039007 | 16.752966 | 4.26E-05 | 0.0013566 | 0.5714286 | 3.0025063 |
| ENSG00000072163 | LIMS2 | - | -4.9735354 | 5.11039007 | 16.752527 | 4.26E-05 | 0.0013566 | 0.3835616 | 2.9671233 |
| ENSG00000113196 | HAND1 | K09071 | -4.9449318 | 6.129146163 | 18.147236 | 2.04E-05 | 0.0009831 | 0.372093 | 2.8651163 |
| ENSG00000074527 | NTN4 | K06845 | -4.9225389 | 5.06363748 | 17.801707 | 2.45E-05 | 0.0011167 | 0.4235669 | 2.861465 |
| ENSG00000091490 | SEL1L3 | - | -4.9225388 | 5.06363748 | 17.80151 | 2.45E-05 | 0.0011167 | 0.4637809 | 3.135159 |
| ENSG00000112414 | ADGRG6 | K08463 | -4.9225386 | 5.06363748 | 17.801035 | 2.45E-05 | 0.0011167 | 0.4712 | 3.0144 |
| ENSG00000186409 | CCDC30 | - | -4.9225384 | 5.06363748 | 17.800409 | 2.45E-05 | 0.0011167 | 0.6679438 | 3.0255428 |
| ENSG00000180376 | CCDC66 | - | -4.9225382 | 5.06363748 | 17.800054 | 2.45E-05 | 0.0011167 | 0.6118143 | 2.9662447 |
| ENSG00000179241 | LDLRAD3 | - | -4.9225381 | 5.06363748 | 17.799692 | 2.45E-05 | 0.0011167 | 0.5333333 | 2.6608696 |
| ENSG00000184730 | APOBR | - | -4.9098374 | 5.051583464 | 18.863917 | 1.40E-05 | 0.0009319 | 0.6216955 | 2.3874202 |
| ENSG00000012779 | ALOX5 | K00461 | -4.9073833 | 5.050463673 | 16.59109 | 4.64E-05 | 0.0013705 | 0.495549 | 3.2255193 |
| ENSG00000135338 | LCA5 | - | -4.8889449 | 5.033270304 | 17.732993 | 2.54E-05 | 0.0011344 | 0.6083214 | 3.0157819 |
| ENSG00000112333 | NR2E1 | K08545 | -4.8889448 | 5.033270304 | 17.732859 | 2.54E-05 | 0.0011344 | 0.4194313 | 2.8625592 |
| ENSG00000013297 | CLDN11 | K06087 | -4.8889448 | 5.033270304 | 17.732832 | 2.54E-05 | 0.0011344 | 0.2801932 | 2.821256 |
| ENSG00000144230 | GPR17 | K08404 | -4.8889446 | 5.033270304 | 17.732408 | 2.54E-05 | 0.0011344 | 0.3242507 | 3.1089918 |
| ENSG00000151743 | AMN1 | - | -4.8887909 | 5.03366521 | 16.544967 | 4.75E-05 | 0.0013705 | 0.4186047 | 2.8217054 |
| ENSG00000162729 | IGSF8 | K06730 | -4.8887909 | 5.03366521 | 16.544891 | 4.75E-05 | 0.0013705 | 0.3800979 | 2.6721044 |
| ENSG00000183690 | EFHC2 | - | -4.8887909 | 5.03366521 | 16.544852 | 4.75E-05 | 0.0013705 | 0.5260347 | 3.2803738 |
| ENSG00000112773 | FAM46A | - | -4.8887908 | 5.03366521 | 16.544785 | 4.75E-05 | 0.0013705 | 0.4875717 | 2.9923518 |
| ENSG00000175874 | CREG2 | - | -4.8887907 | 5.03366521 | 16.544477 | 4.75E-05 | 0.0013705 | 0.3965517 | 2.8862069 |
| ENSG00000169676 | DRD5 | K05840 | -4.8887906 | 5.03366521 | 16.544282 | 4.75E-05 | 0.0013705 | 0.4109015 | 3.0104822 |
| ENSG00000105409 | ATP1A3 | K01539 | -4.8887906 | 5.03366521 | 16.54418 | 4.75E-05 | 0.0013705 | 0.4274062 | 2.8491028 |
| ENSG00000099365 | STX1B | K08486 | -4.8887906 | 5.03366521 | 16.544126 | 4.75E-05 | 0.0013705 | 0.6319444 | 2.9444444 |
| ENSG00000162745 | OLFML2B | - | -4.8887905 | 5.03366521 | 16.544039 | 4.75E-05 | 0.0013705 | 0.5592543 | 2.9107856 |
| ENSG00000103335 | PIEZO1 | - | -4.8887905 | 5.03366521 | 16.543986 | 4.75E-05 | 0.0013705 | 0.4065847 | 3.1725506 |
| ENSG00000144040 | SFXN5 | - | -4.8879884 | 5.031832874 | 18.819219 | 1.44E-05 | 0.0009319 | 0.3852941 | 2.9352941 |
| ENSG00000167528 | ZNF641 | K09228 | -4.887988 | 5.031832874 | 18.818385 | 1.44E-05 | 0.0009319 | 0.5296804 | 2.8401826 |
| ENSG00000185305 | ARL15 | K17201 | -4.8879879 | 5.031832874 | 18.818144 | 1.44E-05 | 0.0009319 | 0.495098 | 2.9705882 |
| ENSG00000006128 | TAC1 | K05239 | -4.8192275 | 4.972331478 | 16.573248 | 4.68E-05 | 0.0013705 | 0.4806202 | 3.2015504 |
| ENSG00000111664 | GNB3 | K07825 | -4.8071137 | 4.95873077 | 18.666521 | 1.56E-05 | 0.0009319 | 0.4823529 | 2.7235294 |
| ENSG00000103248 | MTHFSD | - | -4.7879579 | 4.941385999 | 18.603853 | 1.61E-05 | 0.0009319 | 0.4281984 | 2.845953 |
| ENSG00000161048 | NAPEPLD | K13985 | -4.7879579 | 4.941385999 | 18.603687 | 1.61E-05 | 0.0009319 | 0.5114504 | 3.1933842 |
| ENSG00000105186 | ANKRD27 | K20175 | -4.7879578 | 4.941385999 | 18.603668 | 1.61E-05 | 0.0009319 | 0.4980952 | 2.8761905 |
| ENSG00000165675 | ENOX2 | - | -4.7879578 | 4.941385999 | 18.603624 | 1.61E-05 | 0.0009319 | 0.5327869 | 3.0245902 |
| ENSG00000125648 | SLC25A23 | K14684 | -4.7879578 | 4.941385999 | 18.603532 | 1.61E-05 | 0.0009319 | 0.4647303 | 2.939834 |
| ENSG00000163380 | LMOD3 | - | -4.7879577 | 4.941385999 | 18.603436 | 1.61E-05 | 0.0009319 | 0.6125 | 2.9928571 |
| ENSG00000177045 | SIX5 | K19474 | -4.7879577 | 4.941385999 | 18.603357 | 1.61E-05 | 0.0009319 | 0.3870095 | 2.4749662 |
| ENSG00000164061 | BSN | - | -4.7641771 | 4.920084035 | 18.56266 | 1.64E-05 | 0.0009319 | 0.4887927 | 2.5697911 |
| ENSG00000162989 | KCNJ3 | K04997 | -4.7641768 | 4.920084035 | 18.56214 | 1.64E-05 | 0.0009319 | 0.4750499 | 3.0499002 |
| ENSG00000156298 | TSPAN7 | K06571 | -4.7641764 | 4.920084035 | 18.561223 | 1.65E-05 | 0.0009319 | 0.3947368 | 2.9511278 |
| ENSG00000106479 | ZNF862 | - | -4.7641761 | 4.920084035 | 18.560435 | 1.65E-05 | 0.0009319 | 0.4585115 | 2.9974337 |
| ENSG00000155093 | PTPRN2 | K07817 | -4.7641761 | 4.920084035 | 18.560404 | 1.65E-05 | 0.0009319 | 0.4965517 | 2.7891626 |
| ENSG00000101342 | TLDC2 | - | -4.764176 | 4.920084035 | 18.56036 | 1.65E-05 | 0.0009319 | 0.5162791 | 2.9348837 |
| ENSG00000040487 | PQLC2 | - | -4.7641757 | 4.920084035 | 18.559613 | 1.65E-05 | 0.0009319 | 0.3848797 | 3.0446735 |
| ENSG00000127995 | CASD1 | - | -4.7641757 | 4.920084035 | 18.559586 | 1.65E-05 | 0.0009319 | 0.391468 | 3.314931 |
| ENSG00000024862 | CCDC28A | - | -4.7641754 | 4.920084035 | 18.558818 | 1.65E-05 | 0.0009319 | 0.4963504 | 2.7846715 |
| ENSG00000141560 | FN3KRP | K15523 | -4.7592254 | 4.915037953 | 19.868322 | 8.30E-06 | 0.0007832 | 0.4304207 | 2.9449838 |
| ENSG00000129250 | KIF1C | K10392 | -4.7592254 | 4.915037953 | 19.868164 | 8.30E-06 | 0.0007832 | 0.5095195 | 2.819583 |
| ENSG00000166289 | PLEKHF1 | - | -4.7592253 | 4.915037953 | 19.868093 | 8.30E-06 | 0.0007832 | 0.4516129 | 2.8566308 |
| ENSG00000115758 | ODC1 | K01581 | -4.7592251 | 4.915037953 | 19.867608 | 8.30E-06 | 0.0007832 | 0.494577 | 2.9327549 |
| ENSG00000104522 | TSTA3 | K02377 | -4.7592248 | 4.915037953 | 19.866924 | 8.30E-06 | 0.0007832 | 0.4735202 | 3.0124611 |
| ENSG00000101198 | NKAIN4 | - | -4.7592244 | 4.915037953 | 19.866107 | 8.31E-06 | 0.0007832 | 0.3461538 | 3.1826923 |
| ENSG00000020577 | SAMD4A | - | -4.7592244 | 4.915037953 | 19.866088 | 8.31E-06 | 0.0007832 | 0.494429 | 2.775766 |
| ENSG00000069712 | KIAA1107 | - | -4.7530389 | 4.912574657 | 17.5013 | 2.87E-05 | 0.0011836 | 0.6314623 | 2.6624815 |
| ENSG00000109911 | ELP4 | K11375 | -4.7530388 | 4.912574657 | 17.501236 | 2.87E-05 | 0.0011836 | 0.4392523 | 2.8803738 |
| ENSG00000128596 | CCDC136 | - | -4.7530387 | 4.912574657 | 17.501024 | 2.87E-05 | 0.0011836 | 0.7253033 | 2.9280763 |
| ENSG00000163536 | SERPINI1 | - | -4.7530383 | 4.912574657 | 17.500061 | 2.87E-05 | 0.0011836 | 0.5243902 | 3.0829268 |
| ENSG00000121931 | LRIF1 | - | -4.7510534 | 4.908348709 | 18.546289 | 1.66E-05 | 0.0009319 | 0.5188557 | 2.8010403 |
| ENSG00000152910 | CNTNAP4 | - | -4.7458574 | 4.90370184 | 18.539349 | 1.66E-05 | 0.0009319 | 0.4942792 | 2.914569 |
| ENSG00000135912 | TTLL4 | K16601 | -4.7458569 | 4.90370184 | 18.538087 | 1.67E-05 | 0.0009319 | 0.4937448 | 2.9099249 |
| ENSG00000082397 | EPB41L3 | K06107 | -4.7458566 | 4.90370184 | 18.537563 | 1.67E-05 | 0.0009319 | 0.5915363 | 2.849126 |
| ENSG00000183166 | CALN1 | - | -4.6996895 | 5.905317192 | 18.135037 | 2.06E-05 | 0.0009831 | 0.5440613 | 2.8965517 |
| ENSG00000136854 | STXBP1 | K15292 | -4.6815865 | 5.888578464 | 16.737266 | 4.29E-05 | 0.0013635 | 0.5257048 | 3.053068 |
| ENSG00000073910 | FRY | - | -4.6760487 | 4.841647726 | 17.273036 | 3.24E-05 | 0.0011836 | 0.5061401 | 2.9923664 |
| ENSG00000155545 | MIER3 | - | -4.676048 | 4.841647726 | 17.271572 | 3.24E-05 | 0.0011836 | 0.6468468 | 2.827027 |
| ENSG00000105443 | CYTH2 | K18441 | -4.6760479 | 4.841647726 | 17.271366 | 3.24E-05 | 0.0011836 | 0.5375 | 3.1475 |
| ENSG00000087095 | NLK | K04468 | -4.6361367 | 4.806268434 | 17.199452 | 3.37E-05 | 0.0011836 | 0.3814042 | 2.9240987 |
| ENSG00000112739 | PRPF4B | K08827 | -4.6361367 | 4.806268434 | 17.19944 | 3.37E-05 | 0.0011836 | 0.5342602 | 2.9682224 |
| ENSG00000114767 | RRP9 | K14793 | -4.6361359 | 4.806268434 | 17.197697 | 3.37E-05 | 0.0011836 | 0.4568421 | 2.7347368 |
| ENSG00000168778 | TCTN2 | K19361 | -4.6361358 | 4.806268434 | 17.197424 | 3.37E-05 | 0.0011836 | 0.4677188 | 2.9139168 |
| ENSG00000163735 | CXCL5 | K05506 | -4.6361357 | 4.806268434 | 17.197369 | 3.37E-05 | 0.0011836 | 0.3245614 | 2.6491228 |
| ENSG00000138311 | ZNF365 | K16737 | -4.6361063 | 4.805367489 | 14.89845 | 0.000113456 | 0.0022981 | 0.504329 | 3.0411255 |
| ENSG00000112419 | PHACTR2 | - | -4.6358294 | 6.036367429 | 16.584806 | 4.65E-05 | 0.0013705 | 0.5813953 | 2.7085271 |
| ENSG00000148225 | WDR31 | - | -4.6349918 | 4.804554646 | 18.351536 | 1.84E-05 | 0.0009319 | 0.4332425 | 2.8637602 |
| ENSG00000166575 | TMEM135 | - | -4.6349917 | 4.804554646 | 18.351426 | 1.84E-05 | 0.0009319 | 0.360262 | 3.2489083 |
| ENSG00000077092 | RARB | K08528 | -4.6349917 | 4.804554646 | 18.35128 | 1.84E-05 | 0.0009319 | 0.5111607 | 2.8995536 |
| ENSG00000116095 | PLEKHA3 | K20313 | -4.6310872 | 4.801287541 | 18.380588 | 1.81E-05 | 0.0009319 | 0.5633333 | 2.8566667 |
| ENSG00000137941 | TTLL7 | K16583 | -4.6295551 | 4.799733637 | 18.355762 | 1.83E-05 | 0.0009319 | 0.5197294 | 3.1296505 |
| ENSG00000079950 | STX7 | K08488 | -4.6295551 | 4.799733637 | 18.355702 | 1.83E-05 | 0.0009319 | 0.5977011 | 2.9348659 |
| ENSG00000087494 | PTHLH | - | -4.629555 | 4.799733637 | 18.355492 | 1.83E-05 | 0.0009319 | 0.4641148 | 3.0095694 |
| ENSG00000075914 | EXOSC7 | K12589 | -4.629555 | 4.799733637 | 18.35544 | 1.83E-05 | 0.0009319 | 0.5051546 | 2.7766323 |
| ENSG00000138193 | PLCE1 | K05860 | -4.6295545 | 4.799733637 | 18.354347 | 1.83E-05 | 0.0009319 | 0.5443093 | 2.9070374 |
| ENSG00000166428 | PLD4 | K16860 | -4.6295539 | 4.799733637 | 18.353149 | 1.84E-05 | 0.0009319 | 0.4015595 | 2.9161793 |
| ENSG00000158716 | DUSP23 | K14165 | -4.6295539 | 4.799733637 | 18.353103 | 1.84E-05 | 0.0009319 | 0.36 | 2.9466667 |
| ENSG00000152056 | AP1S3 | K12395 | -4.6295538 | 4.799733637 | 18.352933 | 1.84E-05 | 0.0009319 | 0.5 | 3.347561 |
| ENSG00000171657 | GPR82 | K08420 | -4.6295534 | 4.799733637 | 18.352067 | 1.84E-05 | 0.0009319 | 0.4047619 | 3.2559524 |
| ENSG00000175215 | CTDSP2 | K15731 | -4.6295526 | 4.799733637 | 18.350441 | 1.84E-05 | 0.0009319 | 0.4893617 | 3 |
| ENSG00000182158 | CREB3L2 | K09048 | -4.6295518 | 4.799733637 | 18.348816 | 1.84E-05 | 0.0009319 | 0.5538462 | 2.8653846 |
| ENSG00000111912 | NCOA7 | - | -4.6250613 | 4.796456695 | 17.201891 | 3.36E-05 | 0.0011836 | 0.5870488 | 2.9532909 |
| ENSG00000103154 | NECAB2 | - | -4.6250612 | 4.796456695 | 17.201811 | 3.36E-05 | 0.0011836 | 0.5129534 | 2.888601 |
| ENSG00000101421 | CHMP4B | K12194 | -4.625061 | 4.796456695 | 17.201386 | 3.36E-05 | 0.0011836 | 0.6071429 | 2.8258929 |
| ENSG00000070182 | SPTB | K06115 | -4.625061 | 4.796456695 | 17.201255 | 3.36E-05 | 0.0011836 | 0.5730241 | 3.0339347 |
| ENSG00000124140 | SLC12A5 | K14427 | -4.625061 | 4.796456695 | 17.201234 | 3.36E-05 | 0.0011836 | 0.4363477 | 2.9315189 |
| ENSG00000072134 | EPN2 | K12471 | -4.6250609 | 4.796456695 | 17.201057 | 3.36E-05 | 0.0011836 | 0.4960998 | 2.6224649 |
| ENSG00000088854 | C20orf194 | - | -4.6250608 | 4.796456695 | 17.200961 | 3.36E-05 | 0.0011836 | 0.4919286 | 3.0203908 |
| ENSG00000081138 | CDH7 | K06799 | -4.6250608 | 4.796456695 | 17.200859 | 3.36E-05 | 0.0011836 | 0.5745223 | 2.8866242 |
| ENSG00000067606 | PRKCZ | K18952 | -4.6250608 | 4.796456695 | 17.20082 | 3.36E-05 | 0.0011836 | 0.5118243 | 3.0523649 |
| ENSG00000136750 | GAD2 | K01580 | -4.6250607 | 4.796456695 | 17.200781 | 3.36E-05 | 0.0011836 | 0.4410256 | 2.9794872 |
| ENSG00000130775 | THEMIS2 | - | -4.6250607 | 4.796456695 | 17.200712 | 3.36E-05 | 0.0011836 | 0.4790047 | 2.9844479 |
| ENSG00000020129 | NCDN | - | -4.6250606 | 4.796456695 | 17.200484 | 3.36E-05 | 0.0011836 | 0.4142661 | 2.8052126 |
| ENSG00000158560 | DYNC1I1 | K10415 | -4.6250601 | 4.796456695 | 17.199453 | 3.37E-05 | 0.0011836 | 0.5751938 | 2.9457364 |
| ENSG00000164647 | STEAP1 | K14737 | -4.6250592 | 4.796456695 | 17.197618 | 3.37E-05 | 0.0011836 | 0.3864307 | 3.4867257 |
| ENSG00000165629 | ATP5C1 | K02136 | -4.6250583 | 4.796456695 | 17.195784 | 3.37E-05 | 0.0011836 | 0.4832215 | 2.9060403 |
| ENSG00000166908 | PIP4K2C | K00920 | -4.6250583 | 4.796456695 | 17.195703 | 3.37E-05 | 0.0011836 | 0.4845606 | 3.0498812 |
| ENSG00000168490 | PHYHIP | - | -4.6250582 | 4.796456695 | 17.195623 | 3.37E-05 | 0.0011836 | 0.4939394 | 3.0575758 |
| ENSG00000168702 | LRP1B | K20049 | -4.625058 | 4.796456695 | 17.195061 | 3.37E-05 | 0.0011836 | 0.5383779 | 2.7971298 |
| ENSG00000183837 | PNMA3 | - | -4.625058 | 4.796456695 | 17.195053 | 3.37E-05 | 0.0011836 | 0.4341253 | 2.924406 |
| ENSG00000182901 | RGS7 | K16449 | -4.6250579 | 4.796456695 | 17.195014 | 3.37E-05 | 0.0011836 | 0.550308 | 3.1868583 |
| ENSG00000182621 | PLCB1 | K05858 | -4.6250579 | 4.796456695 | 17.194976 | 3.37E-05 | 0.0011836 | 0.5625 | 3.0328947 |
| ENSG00000181291 | TMEM132E | K17599 | -4.6250579 | 4.796456695 | 17.194938 | 3.37E-05 | 0.0011836 | 0.4627561 | 2.7709497 |
| ENSG00000184220 | CMSS1 | - | -4.6250573 | 4.796456695 | 17.193751 | 3.38E-05 | 0.0011836 | 0.4695341 | 3.0752688 |
| ENSG00000157303 | SUSD3 | - | -4.6094377 | 4.781947482 | 18.325228 | 1.86E-05 | 0.0009319 | 0.345098 | 2.6235294 |
| ENSG00000147684 | NDUFB9 | K03965 | -4.6094374 | 4.781947482 | 18.324446 | 1.86E-05 | 0.0009319 | 0.520362 | 3.3122172 |
| ENSG00000005700 | IBTK | - | -4.6094372 | 4.781947482 | 18.324117 | 1.86E-05 | 0.0009319 | 0.4863267 | 2.924612 |
| ENSG00000095066 | HOOK2 | K16611 | -4.6094371 | 4.781947482 | 18.323948 | 1.86E-05 | 0.0009319 | 0.5952712 | 2.9415855 |
| ENSG00000137842 | TMEM62 | - | -4.6094371 | 4.781947482 | 18.323943 | 1.86E-05 | 0.0009319 | 0.3716952 | 3.2597201 |
| ENSG00000139163 | ETNK1 | K00894 | -4.6094371 | 4.781947482 | 18.32383 | 1.86E-05 | 0.0009319 | 0.4513274 | 3.090708 |
| ENSG00000145863 | GABRA6 | K05175 | -4.609437 | 4.781947482 | 18.323718 | 1.86E-05 | 0.0009319 | 0.4569536 | 3.1368653 |
| ENSG00000074276 | CDHR2 | K16502 | -4.6094369 | 4.781947482 | 18.323562 | 1.86E-05 | 0.0009319 | 0.5351145 | 2.780916 |
| ENSG00000103266 | STUB1 | K09561 | -4.6094367 | 4.781947482 | 18.323127 | 1.86E-05 | 0.0009319 | 0.5610561 | 2.980198 |
| ENSG00000172037 | LAMB2 | K06243 | -4.5949907 | 5.809932856 | 16.117018 | 5.95E-05 | 0.0015181 | 0.4560623 | 2.6206897 |
| ENSG00000167619 | TMEM145 | - | -4.5672287 | 4.745290209 | 17.048369 | 3.64E-05 | 0.001251 | 0.3752536 | 3.2454361 |
| ENSG00000144290 | SLC4A10 | K13861 | -4.549735 | 5.769816525 | 15.123073 | 0.000100725 | 0.002175 | 0.4534884 | 3.0536673 |
| ENSG00000108639 | SYNGR2 | - | -4.5306312 | 4.71034044 | 16.59709 | 4.62E-05 | 0.0013705 | 0.3418182 | 3.0327273 |
| ENSG00000006015 | C19orf60 | - | -4.5247809 | 4.705190038 | 16.592874 | 4.63E-05 | 0.0013705 | 0.4577114 | 2.9004975 |
| ENSG00000137962 | ARHGAP29 | K20644 | -4.5247807 | 4.705190038 | 16.592471 | 4.63E-05 | 0.0013705 | 0.5440127 | 2.925456 |
| ENSG00000148965 | SAA4 | K17310 | -4.5247807 | 4.705190038 | 16.592352 | 4.63E-05 | 0.0013705 | 0.4692308 | 3.0076923 |
| ENSG00000123066 | MED13L | K15164 | -4.5247806 | 4.705190038 | 16.592182 | 4.63E-05 | 0.0013705 | 0.4886878 | 2.8226244 |
| ENSG00000087448 | KLHL42 | - | -4.5247806 | 4.705190038 | 16.592109 | 4.63E-05 | 0.0013705 | 0.4871287 | 2.990099 |
| ENSG00000132254 | ARFIP2 | K20314 | -4.5247806 | 4.705190038 | 16.592083 | 4.63E-05 | 0.0013705 | 0.5160428 | 2.8850267 |
| ENSG00000162909 | CAPN2 | K03853 | -4.5247805 | 4.705190038 | 16.591969 | 4.63E-05 | 0.0013705 | 0.5442857 | 3.0857143 |
| ENSG00000168071 | CCDC88B | - | -4.5247805 | 4.705190038 | 16.591963 | 4.63E-05 | 0.0013705 | 0.554878 | 2.7669377 |
| ENSG00000168899 | VAMP5 | K08514 | -4.5247804 | 4.705190038 | 16.591732 | 4.64E-05 | 0.0013705 | 0.4655172 | 2.8017241 |
| ENSG00000121964 | GTDC1 | - | -4.5247804 | 4.705190038 | 16.591709 | 4.64E-05 | 0.0013705 | 0.4672489 | 3.1943231 |
| ENSG00000111077 | TNS2 | K18080 | -4.5247803 | 4.705190038 | 16.591526 | 4.64E-05 | 0.0013705 | 0.4291755 | 2.7787174 |
| ENSG00000100416 | TRMU | K21027 | -4.5247802 | 4.705190038 | 16.591343 | 4.64E-05 | 0.0013705 | 0.4346793 | 3.0760095 |
| ENSG00000172572 | PDE3A | K19021 | -4.5247799 | 4.705190038 | 16.590643 | 4.64E-05 | 0.0013705 | 0.4890447 | 2.7931639 |
| ENSG00000185222 | TCEAL9 | - | -4.5247793 | 4.705190038 | 16.589553 | 4.64E-05 | 0.0013705 | 0.6730769 | 3.2884615 |
| ENSG00000166073 | GPR176 | K08442 | -4.5235653 | 5.746002735 | 15.854116 | 6.84E-05 | 0.0016606 | 0.407767 | 3.0330097 |
| ENSG00000172548 | NIPAL4 | - | -4.4961999 | 4.680101062 | 16.533838 | 4.78E-05 | 0.0013705 | 0.3583691 | 2.9206009 |
| ENSG00000188542 | DUSP28 | K14165 | -4.4961999 | 4.680101062 | 16.533835 | 4.78E-05 | 0.0013705 | 0.3295455 | 2.5340909 |
| ENSG00000168060 | NAALADL1 | K01301 | -4.4961999 | 4.680101062 | 16.53382 | 4.78E-05 | 0.0013705 | 0.4538559 | 2.8609355 |
| ENSG00000100276 | RASL10A | K07850 | -4.4961997 | 4.680101062 | 16.533556 | 4.78E-05 | 0.0013705 | 0.3399015 | 2.9310345 |
| ENSG00000099864 | PALM | K16519 | -4.4961997 | 4.680101062 | 16.533435 | 4.78E-05 | 0.0013705 | 0.5891473 | 2.6072351 |
| ENSG00000023228 | NDUFS1 | K03934 | -4.4961996 | 4.680101062 | 16.533321 | 4.78E-05 | 0.0013705 | 0.4574899 | 2.7773279 |
| ENSG00000099282 | TSPAN15 | K17297 | -4.4961996 | 4.680101062 | 16.533238 | 4.78E-05 | 0.0013705 | 0.4115646 | 3.1360544 |
| ENSG00000167851 | CD300A | K06719 | -4.4900814 | 4.674126327 | 17.670704 | 2.63E-05 | 0.0011496 | 0.4849498 | 2.9565217 |
| ENSG00000155465 | SLC7A7 | K13867 | -4.4900813 | 4.674126327 | 17.670523 | 2.63E-05 | 0.0011496 | 0.37182 | 3.0939335 |
| ENSG00000136830 | FAM129B | - | -4.4900813 | 4.674126327 | 17.670457 | 2.63E-05 | 0.0011496 | 0.5040214 | 3.0053619 |
| ENSG00000103356 | EARS2 | K01885 | -4.4900813 | 4.674126327 | 17.670455 | 2.63E-05 | 0.0011496 | 0.4438202 | 2.9775281 |
| ENSG00000115616 | SLC9A2 | K14722 | -4.4900812 | 4.674126327 | 17.670305 | 2.63E-05 | 0.0011496 | 0.4137931 | 3.1502463 |
| ENSG00000059377 | TBXAS1 | K01832 | -4.4803858 | 4.666253535 | 16.506398 | 4.85E-05 | 0.0013705 | 0.4034483 | 3.1275862 |
| ENSG00000148180 | GSN | K05768 | -4.4803857 | 4.666253535 | 16.506075 | 4.85E-05 | 0.0013705 | 0.4757033 | 2.8452685 |
| ENSG00000185453 | C19orf68 | - | -4.4803856 | 4.666253535 | 16.50597 | 4.85E-05 | 0.0013705 | 0.4043478 | 2.8271739 |
| ENSG00000125821 | DTD1 | K07560 | -4.4759188 | 5.704056155 | 16.934361 | 3.87E-05 | 0.0013205 | 0.5201794 | 2.9910314 |
| ENSG00000115183 | TANC1 | - | -4.4741147 | 4.660763813 | 16.491684 | 4.89E-05 | 0.0013705 | 0.4744761 | 2.7307899 |
| ENSG00000103485 | QPRT | K00767 | -4.4741146 | 4.660763813 | 16.491589 | 4.89E-05 | 0.0013705 | 0.3434343 | 2.6498317 |
| ENSG00000124194 | GDAP1L1 | - | -4.4741146 | 4.660763813 | 16.491585 | 4.89E-05 | 0.0013705 | 0.4507772 | 3.1088083 |
| ENSG00000101187 | SLCO4A1 | K14354 | -4.4741146 | 4.660763813 | 16.491494 | 4.89E-05 | 0.0013705 | 0.3587258 | 2.8268698 |
| ENSG00000171867 | PRNP | K05634 | -4.4741144 | 4.660763813 | 16.49106 | 4.89E-05 | 0.0013705 | 0.3873518 | 2.8142292 |
| ENSG00000132965 | ALOX5AP | K20735 | -4.4741143 | 4.660763813 | 16.490851 | 4.89E-05 | 0.0013705 | 0.3944954 | 3.1009174 |
| ENSG00000105695 | MAG | K06771 | -4.4712815 | 5.699542664 | 15.64437 | 7.64E-05 | 0.0016986 | 0.4824281 | 2.9153355 |
| ENSG00000135414 | GDF11 | K05497 | -4.4116264 | 5.64555175 | 14.303333 | 0.000155589 | 0.0026567 | 0.4103194 | 2.9017199 |
| ENSG00000171885 | AQP4 | K09866 | -4.4052802 | 5.639948866 | 14.282393 | 0.00015733 | 0.0026567 | 0.3529412 | 2.879257 |
| ENSG00000149289 | ZC3H12C | K18668 | -4.4037121 | 5.828215641 | 15.377408 | 8.80E-05 | 0.0019487 | 0.5542986 | 2.9151584 |
| ENSG00000013016 | EHD3 | K12476 | -4.3850866 | 4.58178424 | 16.112817 | 5.97E-05 | 0.0015181 | 0.470696 | 3.0860806 |
| ENSG00000110047 | EHD1 | K12483 | -4.385086 | 4.58178424 | 16.111659 | 5.97E-05 | 0.0015181 | 0.4671533 | 3.0784672 |
| ENSG00000164626 | KCNK5 | K04916 | -4.3700833 | 4.569497011 | 16.256501 | 5.53E-05 | 0.0015016 | 0.4549098 | 3.006012 |
| ENSG00000173638 | SLC19A1 | K14609 | -4.3700832 | 4.569497011 | 16.2563 | 5.53E-05 | 0.0015016 | 0.3688663 | 3.0135364 |
| ENSG00000167315 | ACAA2 | K07508 | -4.370083 | 4.569497011 | 16.255952 | 5.53E-05 | 0.0015016 | 0.4105793 | 2.6372796 |
| ENSG00000105289 | TJP3 | K06097 | -4.3700829 | 4.569497011 | 16.255856 | 5.53E-05 | 0.0015016 | 0.5181237 | 2.7579957 |
| ENSG00000162520 | SYNC | K10377 | -4.3700829 | 4.569497011 | 16.255753 | 5.53E-05 | 0.0015016 | 0.6680498 | 2.9190871 |
| ENSG00000144029 | MRPS5 | K02988 | -4.3700825 | 4.569497011 | 16.255123 | 5.54E-05 | 0.0015016 | 0.4023256 | 2.8860465 |
| ENSG00000119402 | FBXW2 | K10261 | -4.3700823 | 4.569497011 | 16.254736 | 5.54E-05 | 0.0015016 | 0.4801762 | 3.1035242 |
| ENSG00000138722 | MMRN1 | - | -4.3700822 | 4.569497011 | 16.254493 | 5.54E-05 | 0.0015016 | 0.5407166 | 2.9242671 |
| ENSG00000135269 | TES | - | -4.3700822 | 4.569497011 | 16.254485 | 5.54E-05 | 0.0015016 | 0.4655582 | 2.9619952 |
| ENSG00000123444 | KBTBD4 | K10472 | -4.3700822 | 4.569497011 | 16.254374 | 5.54E-05 | 0.0015016 | 0.5046041 | 2.9668508 |
| ENSG00000132470 | ITGB4 | K06525 | -4.3700819 | 4.569497011 | 16.253917 | 5.54E-05 | 0.0015016 | 0.5043908 | 2.8254665 |
| ENSG00000116106 | EPHA4 | K05105 | -4.3557415 | 5.786069911 | 16.160046 | 5.82E-05 | 0.0015181 | 0.4969574 | 2.8904665 |
| ENSG00000094914 | AAAS | K14320 | -4.3557413 | 5.786069911 | 16.160802 | 5.82E-05 | 0.0015181 | 0.3864469 | 2.8901099 |
| ENSG00000174132 | FAM174A | - | -4.3416516 | 5.584888294 | 15.187806 | 9.73E-05 | 0.0021056 | 0.4052632 | 2.5263158 |
| ENSG00000161996 | WDR90 | - | -4.3331991 | 5.577035992 | 16.096113 | 6.02E-05 | 0.0015239 | 0.3965714 | 2.7325714 |
| ENSG00000116005 | PCYOX1 | K05906 | -4.3314195 | 4.535388116 | 17.354615 | 3.10E-05 | 0.0011836 | 0.4712871 | 3.0594059 |
| ENSG00000133574 | GIMAP4 | - | -4.3314188 | 4.535388116 | 17.353243 | 3.10E-05 | 0.0011836 | 0.5335277 | 2.9504373 |
| ENSG00000138031 | ADCY3 | K08043 | -4.3314185 | 4.535388116 | 17.352773 | 3.10E-05 | 0.0011836 | 0.4628821 | 3.0393013 |
| ENSG00000135451 | TROAP | - | -4.3314185 | 4.535388116 | 17.35274 | 3.10E-05 | 0.0011836 | 0.4389401 | 2.6739631 |
| ENSG00000184584 | TMEM173 | K12654 | -4.3314185 | 4.535388116 | 17.35273 | 3.10E-05 | 0.0011836 | 0.4379947 | 2.9709763 |
| ENSG00000174697 | LEP | K05424 | -4.3314182 | 4.535388116 | 17.352148 | 3.11E-05 | 0.0011836 | 0.4610778 | 3.011976 |
| ENSG00000068831 | RASGRP2 | K12361 | -4.3247042 | 4.529587337 | 17.346681 | 3.11E-05 | 0.0011836 | 0.4639344 | 3.0344262 |
| ENSG00000119514 | GALNT12 | K00710 | -4.324704 | 4.529587337 | 17.346285 | 3.12E-05 | 0.0011836 | 0.4716007 | 3.1101549 |
| ENSG00000085831 | TTC39A | - | -4.324704 | 4.529587337 | 17.346211 | 3.12E-05 | 0.0011836 | 0.4730832 | 3.0831974 |
| ENSG00000117385 | P3H1 | K08134 | -4.324704 | 4.529587337 | 17.346176 | 3.12E-05 | 0.0011836 | 0.4987562 | 3.0124378 |
| ENSG00000091409 | ITGA6 | K06485 | -4.3247039 | 4.529587337 | 17.345972 | 3.12E-05 | 0.0011836 | 0.5115044 | 2.9637168 |
| ENSG00000139117 | CPNE8 | - | -4.3247038 | 4.529587337 | 17.345853 | 3.12E-05 | 0.0011836 | 0.5035461 | 3.0035461 |
| ENSG00000137767 | SQRDL | K17218 | -4.3247038 | 4.529587337 | 17.345778 | 3.12E-05 | 0.0011836 | 0.4266667 | 2.9955556 |
| ENSG00000149260 | CAPN5 | K08574 | -4.3247037 | 4.529587337 | 17.345683 | 3.12E-05 | 0.0011836 | 0.475 | 3.0294118 |
| ENSG00000156413 | FUT6 | K07634 | -4.3247036 | 4.529587337 | 17.345512 | 3.12E-05 | 0.0011836 | 0.4340659 | 3.2472527 |
| ENSG00000163995 | ABLIM2 | K07520 | -4.3247031 | 4.529587337 | 17.344586 | 3.12E-05 | 0.0011836 | 0.496904 | 2.7724458 |
| ENSG00000171124 | FUT3 | K00716 | -4.3247027 | 4.529587337 | 17.343771 | 3.12E-05 | 0.0011836 | 0.4293629 | 3.2825485 |
| ENSG00000187800 | PEAR1 | - | -4.3247026 | 4.529587337 | 17.343628 | 3.12E-05 | 0.0011836 | 0.4040501 | 2.4966249 |
| ENSG00000176390 | CRLF3 | - | -4.3247026 | 4.529587337 | 17.343595 | 3.12E-05 | 0.0011836 | 0.5452489 | 2.9457014 |
| ENSG00000182782 | HCAR2 | K08402 | -4.3247025 | 4.529587337 | 17.343471 | 3.12E-05 | 0.0011836 | 0.3333333 | 3.3085399 |
| ENSG00000008323 | PLEKHG6 | - | -4.3236193 | 4.533172514 | 15.225548 | 9.54E-05 | 0.0020724 | 0.4860759 | 2.9417722 |
| ENSG00000054983 | GALC | K01202 | -4.3236193 | 4.533172514 | 15.225531 | 9.54E-05 | 0.0020724 | 0.459854 | 3.1357664 |
| ENSG00000022277 | RTFDC1 | - | -4.3236192 | 4.533172514 | 15.225472 | 9.54E-05 | 0.0020724 | 0.5059524 | 2.8244048 |
| ENSG00000065183 | WDR3 | K14556 | -4.3236192 | 4.533172514 | 15.225398 | 9.54E-05 | 0.0020724 | 0.4984093 | 3.0053022 |
| ENSG00000196072 | BLOC1S2 | K16750 | -4.3236192 | 4.533172514 | 15.225395 | 9.54E-05 | 0.0020724 | 0.6126761 | 2.915493 |
| ENSG00000101489 | CELF4 | K13207 | -4.3236191 | 4.533172514 | 15.225252 | 9.54E-05 | 0.0020724 | 0.409465 | 2.6975309 |
| ENSG00000119042 | SATB2 | - | -4.323619 | 4.533172514 | 15.225112 | 9.54E-05 | 0.0020724 | 0.5225102 | 2.9099591 |
| ENSG00000161082 | CELF5 | K13207 | -4.323619 | 4.533172514 | 15.225029 | 9.54E-05 | 0.0020724 | 0.4103093 | 2.814433 |
| ENSG00000167281 | RBFOX3 | K14946 | -4.3236189 | 4.533172514 | 15.224946 | 9.54E-05 | 0.0020724 | 0.4385475 | 2.8826816 |
| ENSG00000101349 | PAK7 | K05736 | -4.3228269 | 5.568396601 | 15.118937 | 0.000100946 | 0.0021756 | 0.5201669 | 2.9513213 |
| ENSG00000103995 | CEP152 | K16728 | -4.3205928 | 4.52675396 | 16.139936 | 5.88E-05 | 0.0015181 | 0.6157895 | 2.9339181 |
| ENSG00000188315 | C3orf62 | - | -4.3128063 | 5.559932297 | 16.081597 | 6.07E-05 | 0.0015323 | 0.5430712 | 2.8876404 |
| ENSG00000063015 | SEZ6 | - | -4.3087889 | 6.165283577 | 14.424571 | 0.000145886 | 0.0026567 | 0.4617706 | 2.806841 |
| ENSG00000104490 | NCALD | K19695 | -4.3067891 | 4.514860869 | 16.11167 | 5.97E-05 | 0.0015181 | 0.5773196 | 3.1185567 |
| ENSG00000102468 | HTR2A | K04157 | -4.3067889 | 4.514860869 | 16.111334 | 5.97E-05 | 0.0015181 | 0.4585987 | 3.0106157 |
| ENSG00000104722 | NEFM | K04573 | -4.3067888 | 4.514860869 | 16.111236 | 5.97E-05 | 0.0015181 | 0.6965066 | 2.7980349 |
| ENSG00000091664 | SLC17A6 | K12302 | -4.3067887 | 4.514860869 | 16.110998 | 5.97E-05 | 0.0015181 | 0.4106529 | 3.0292096 |
| ENSG00000115756 | HPCAL1 | - | -4.3067885 | 4.514860869 | 16.110559 | 5.97E-05 | 0.0015181 | 0.5803109 | 3.1295337 |
| ENSG00000111783 | RFX4 | K09174 | -4.3067883 | 4.514860869 | 16.110258 | 5.98E-05 | 0.0015181 | 0.5094086 | 3.0026882 |
| ENSG00000106615 | RHEB | K07208 | -4.3067881 | 4.514860869 | 16.109957 | 5.98E-05 | 0.0015181 | 0.5217391 | 2.9728261 |
| ENSG00000176946 | THAP4 | - | -4.306788 | 4.514860869 | 16.109687 | 5.98E-05 | 0.0015181 | 0.4818024 | 2.7019064 |
| ENSG00000120539 | MASTL | K16309 | -4.3067879 | 4.514860869 | 16.1096 | 5.98E-05 | 0.0015181 | 0.5426621 | 2.8122867 |
| ENSG00000186642 | PDE2A | K18283 | -4.3067878 | 4.514860869 | 16.109274 | 5.98E-05 | 0.0015181 | 0.501594 | 2.9617428 |
| ENSG00000169155 | ZBTB43 | K10514 | -4.3067877 | 4.514860869 | 16.109115 | 5.98E-05 | 0.0015181 | 0.5738758 | 2.8736617 |
| ENSG00000158856 | DMTN | - | -4.3067876 | 4.514860869 | 16.108958 | 5.98E-05 | 0.0015181 | 0.5358025 | 2.8962963 |
| ENSG00000162409 | PRKAA2 | K07198 | -4.3067874 | 4.514860869 | 16.108705 | 5.98E-05 | 0.0015181 | 0.4655797 | 3.0144928 |
| ENSG00000122585 | NPY | K05232 | -4.3067874 | 4.514860869 | 16.108642 | 5.98E-05 | 0.0015181 | 0.4845361 | 2.8865979 |
| ENSG00000168140 | VASN | - | -4.3067874 | 4.514860869 | 16.108544 | 5.98E-05 | 0.0015181 | 0.3863299 | 2.7057949 |
| ENSG00000166579 | NDEL1 | K16739 | -4.3067873 | 4.514860869 | 16.108451 | 5.98E-05 | 0.0015181 | 0.5826087 | 2.826087 |
| ENSG00000158467 | AHCYL2 | K01251 | -4.3067873 | 4.514860869 | 16.108386 | 5.98E-05 | 0.0015181 | 0.4648118 | 2.7659574 |
| ENSG00000157617 | C2CD2 | - | -4.3067871 | 4.514860869 | 16.107987 | 5.98E-05 | 0.0015181 | 0.4712644 | 2.7643678 |
| ENSG00000167971 | CASKIN1 | - | -4.306787 | 4.514860869 | 16.107973 | 5.98E-05 | 0.0015181 | 0.408805 | 2.580014 |
| ENSG00000141934 | PLPP2 | K01080 | -4.306787 | 4.514860869 | 16.107867 | 5.98E-05 | 0.0015181 | 0.3851133 | 3.0453074 |
| ENSG00000125734 | GPR108 | - | -4.3067869 | 4.514860869 | 16.107684 | 5.98E-05 | 0.0015181 | 0.4014733 | 3.1104972 |
| ENSG00000153071 | DAB2 | K12475 | -4.3067868 | 4.514860869 | 16.107588 | 5.98E-05 | 0.0015181 | 0.512987 | 2.7116883 |
| ENSG00000104983 | CCDC61 | K16755 | -4.2998262 | 4.508157733 | 17.304498 | 3.18E-05 | 0.0011836 | 0.4960938 | 2.7617188 |
| ENSG00000128340 | RAC2 | K07860 | -4.2998254 | 4.508157733 | 17.30306 | 3.19E-05 | 0.0011836 | 0.4635417 | 3 |
| ENSG00000145868 | FBXO38 | K10313 | -4.299825 | 4.508157733 | 17.302292 | 3.19E-05 | 0.0011836 | 0.5185185 | 2.9074074 |
| ENSG00000132004 | FBXW9 | K10265 | -4.299825 | 4.508157733 | 17.302256 | 3.19E-05 | 0.0011836 | 0.4728033 | 2.834728 |
| ENSG00000130779 | CLIP1 | K10421 | -4.2958621 | 5.733383511 | 16.210711 | 5.67E-05 | 0.0015181 | 0.6363004 | 2.8331015 |
| ENSG00000111775 | COX6A1 | K02266 | -4.2907561 | 5.728636392 | 14.917787 | 0.000112299 | 0.0022981 | 0.3669725 | 2.9908257 |
| ENSG00000117595 | IRF6 | K10154 | -4.2583409 | 5.511065365 | 14.850739 | 0.000116362 | 0.0023123 | 0.4946467 | 3.0942184 |
| ENSG00000156076 | WIF1 | K01691 | -4.2514027 | 5.505022632 | 14.827386 | 0.000117812 | 0.002329 | 0.3799472 | 2.76781 |
| ENSG00000160285 | LSS | K01852 | -4.2471453 | 5.501671605 | 15.819077 | 6.97E-05 | 0.0016606 | 0.4494536 | 3.0751366 |
| ENSG00000136816 | TOR1B | - | -4.2471452 | 5.501671605 | 15.819109 | 6.97E-05 | 0.0016606 | 0.4107143 | 3.1309524 |
| ENSG00000087076 | HSD17B14 | - | -4.2471452 | 5.501671605 | 15.819228 | 6.97E-05 | 0.0016606 | 0.4 | 2.6037037 |
| ENSG00000106976 | DNM1 | K01528 | -4.2327856 | 5.488498304 | 13.698799 | 0.000214592 | 0.0029188 | 0.5046296 | 2.9560185 |
| ENSG00000173267 | SNCG | - | -4.2327853 | 5.488498304 | 13.699435 | 0.000214519 | 0.0029188 | 0.6062992 | 2.4724409 |
| ENSG00000127955 | GNAI1 | K04630 | -4.2256931 | 5.482676077 | 14.739681 | 0.000123421 | 0.0024067 | 0.5310734 | 3.0451977 |
| ENSG00000154359 | LONRF1 | - | -4.1799879 | 5.630985305 | 14.213634 | 0.000163184 | 0.0026883 | 0.4514877 | 2.9327296 |
| ENSG00000168884 | TNIP2 | - | -4.1554893 | 5.82315552 | 15.598609 | 7.83E-05 | 0.0017368 | 0.5361305 | 2.7878788 |
| ENSG00000186132 | C2orf76 | - | -4.1544214 | 4.383924178 | 15.780782 | 7.11E-05 | 0.0016606 | 0.515873 | 3.2698413 |
| ENSG00000183722 | LHFP | - | -4.154421 | 4.383924178 | 15.780161 | 7.11E-05 | 0.0016606 | 0.33 | 2.82 |
| ENSG00000170791 | CHCHD7 | - | -4.1544208 | 4.383924178 | 15.7798 | 7.12E-05 | 0.0016606 | 0.5090909 | 3.0727273 |
| ENSG00000182580 | EPHB3 | K05112 | -4.1544207 | 4.383924178 | 15.779685 | 7.12E-05 | 0.0016606 | 0.4689379 | 2.8787575 |
| ENSG00000168955 | TM4SF20 | - | -4.1544207 | 4.383924178 | 15.779628 | 7.12E-05 | 0.0016606 | 0.3755459 | 2.9126638 |
| ENSG00000172336 | POP7 | K14527 | -4.1544204 | 4.383924178 | 15.779209 | 7.12E-05 | 0.0016606 | 0.4714286 | 2.85 |
| ENSG00000163590 | PPM1L | K17506 | -4.1544203 | 4.383924178 | 15.779045 | 7.12E-05 | 0.0016606 | 0.5166667 | 3.0861111 |
| ENSG00000127951 | FGL2 | - | -4.15442 | 4.383924178 | 15.778463 | 7.12E-05 | 0.0016606 | 0.5261959 | 3.0728929 |
| ENSG00000126460 | PRRG2 | - | -4.1544196 | 4.383924178 | 15.777799 | 7.12E-05 | 0.0016606 | 0.4455446 | 2.970297 |
| ENSG00000123485 | HJURP | - | -4.1544194 | 4.383924178 | 15.777432 | 7.12E-05 | 0.0016606 | 0.5173797 | 2.8061497 |
| ENSG00000111666 | CHPT1 | K00994 | -4.1544191 | 4.383924178 | 15.777037 | 7.13E-05 | 0.0016606 | 0.3571429 | 3.1330049 |
| ENSG00000107815 | C10orf2 | K17680 | -4.1544189 | 4.383924178 | 15.776643 | 7.13E-05 | 0.0016606 | 0.4576023 | 3.0248538 |
| ENSG00000146216 | TTBK1 | K08815 | -4.1264076 | 4.360266524 | 15.710959 | 7.38E-05 | 0.0016606 | 0.5185466 | 2.6343679 |
| ENSG00000167123 | CERCAM | - | -4.1264075 | 4.360266524 | 15.710832 | 7.38E-05 | 0.0016606 | 0.4588235 | 3.0840336 |
| ENSG00000184164 | CRELD2 | - | -4.1264075 | 4.360266524 | 15.710794 | 7.38E-05 | 0.0016606 | 0.5174129 | 2.5621891 |
| ENSG00000133138 | TBC1D8B | K19951 | -4.1264074 | 4.360266524 | 15.710675 | 7.38E-05 | 0.0016606 | 0.53125 | 3.1410714 |
| ENSG00000122756 | CNTFR | K05059 | -4.1264072 | 4.360266524 | 15.710347 | 7.38E-05 | 0.0016606 | 0.422043 | 2.8951613 |
| ENSG00000107862 | GBF1 | K18443 | -4.1264072 | 4.360266524 | 15.710298 | 7.38E-05 | 0.0016606 | 0.4970414 | 2.8972566 |
| ENSG00000107443 | CCNJ | - | -4.1264071 | 4.360266524 | 15.710248 | 7.38E-05 | 0.0016606 | 0.4751958 | 3.1227154 |
| ENSG00000105258 | POLR2I | K03017 | -4.1264071 | 4.360266524 | 15.710199 | 7.38E-05 | 0.0016606 | 0.584 | 3 |
| ENSG00000101353 | MROH8 | - | -4.1264067 | 4.360266524 | 15.709596 | 7.38E-05 | 0.0016606 | 0.4602432 | 3.0467727 |
| ENSG00000066468 | FGFR2 | K05093 | -4.1264065 | 4.360266524 | 15.70913 | 7.39E-05 | 0.0016606 | 0.5060827 | 2.9476886 |
| ENSG00000071073 | MGAT4A | K00738 | -4.1264064 | 4.360266524 | 15.709078 | 7.39E-05 | 0.0016606 | 0.4953271 | 3.2336449 |
| ENSG00000013563 | DNASE1L1 | K11995 | -4.1264064 | 4.360266524 | 15.709075 | 7.39E-05 | 0.0016606 | 0.486755 | 3.0463576 |
| ENSG00000100441 | KHNYN | - | -4.1264064 | 4.360266524 | 15.708993 | 7.39E-05 | 0.0016606 | 0.4469027 | 2.79941 |
| ENSG00000123360 | PDE1B | K13755 | -4.1196774 | 5.579649836 | 14.287753 | 0.000156882 | 0.0026567 | 0.5410448 | 3.0690299 |
| ENSG00000137996 | RTCA | K01974 | -4.1186684 | 4.353737494 | 15.694458 | 7.44E-05 | 0.0016606 | 0.4010554 | 2.7467018 |
| ENSG00000128564 | VGF | - | -4.1186683 | 4.353737494 | 15.694347 | 7.44E-05 | 0.0016606 | 0.5642276 | 2.7121951 |
| ENSG00000130803 | ZNF317 | K09228 | -4.1186683 | 4.353737494 | 15.694335 | 7.44E-05 | 0.0016606 | 0.4588235 | 2.9260504 |
| ENSG00000118420 | UBE3D | K20803 | -4.1186682 | 4.353737494 | 15.694131 | 7.45E-05 | 0.0016606 | 0.4524422 | 2.9717224 |
| ENSG00000116691 | MIIP | - | -4.1186681 | 4.353737494 | 15.694057 | 7.45E-05 | 0.0016606 | 0.4819588 | 2.7835052 |
| ENSG00000116212 | LRRC42 | - | -4.1186681 | 4.353737494 | 15.693983 | 7.45E-05 | 0.0016606 | 0.5140187 | 3 |
| ENSG00000142185 | TRPM2 | K04977 | -4.118668 | 4.353737494 | 15.6939 | 7.45E-05 | 0.0016606 | 0.4455892 | 3.1030264 |
| ENSG00000110911 | SLC11A2 | K21398 | -4.1186678 | 4.353737494 | 15.69348 | 7.45E-05 | 0.0016606 | 0.3830508 | 3.0288136 |
| ENSG00000127418 | FGFRL1 | - | -4.1186678 | 4.353737494 | 15.693462 | 7.45E-05 | 0.0016606 | 0.3690476 | 2.797619 |
| ENSG00000110786 | PTPN5 | K18018 | -4.1186676 | 4.353737494 | 15.693173 | 7.45E-05 | 0.0016606 | 0.520354 | 2.9681416 |
| ENSG00000108091 | CCDC6 | K09288 | -4.1186676 | 4.353737494 | 15.693154 | 7.45E-05 | 0.0016606 | 0.5696203 | 2.8248945 |
| ENSG00000145819 | ARHGAP26 | K20071 | -4.1186675 | 4.353737494 | 15.692949 | 7.45E-05 | 0.0016606 | 0.534398 | 2.9987715 |
| ENSG00000108852 | MPP2 | - | -4.1186674 | 4.353737494 | 15.692866 | 7.45E-05 | 0.0016606 | 0.4757119 | 2.9296482 |
| ENSG00000106125 | FAM188B | - | -4.1186671 | 4.353737494 | 15.692319 | 7.45E-05 | 0.0016606 | 0.5138705 | 2.8956407 |
| ENSG00000092094 | OSGEP | K01409 | -4.1186669 | 4.353737494 | 15.692121 | 7.45E-05 | 0.0016606 | 0.4268657 | 2.7492537 |
| ENSG00000150672 | DLG2 | K12075 | -4.1186669 | 4.353737494 | 15.691997 | 7.45E-05 | 0.0016606 | 0.5405128 | 2.9353846 |
| ENSG00000171365 | CLCN5 | K05012 | -4.1186667 | 4.353737494 | 15.691676 | 7.46E-05 | 0.0016606 | 0.4203431 | 3.0379902 |
| ENSG00000105767 | CADM4 | K06783 | -4.1186666 | 4.353737494 | 15.691485 | 7.46E-05 | 0.0016606 | 0.4716495 | 2.8376289 |
| ENSG00000099889 | ARVCF | - | -4.1186665 | 4.353737494 | 15.691445 | 7.46E-05 | 0.0016606 | 0.4719335 | 2.7027027 |
| ENSG00000175931 | UBE2O | K10581 | -4.1186665 | 4.353737494 | 15.691425 | 7.46E-05 | 0.0016606 | 0.5356037 | 2.7794118 |
| ENSG00000101442 | ACTR5 | K11672 | -4.1186665 | 4.353737494 | 15.691353 | 7.46E-05 | 0.0016606 | 0.5074135 | 2.9719934 |
| ENSG00000176597 | B3GNT5 | K03766 | -4.1186664 | 4.353737494 | 15.691253 | 7.46E-05 | 0.0016606 | 0.473545 | 3.2989418 |
| ENSG00000185477 | GPRIN3 | - | -4.1186664 | 4.353737494 | 15.691247 | 7.46E-05 | 0.0016606 | 0.5399485 | 2.4845361 |
| ENSG00000183783 | KCTD8 | - | -4.1186663 | 4.353737494 | 15.691081 | 7.46E-05 | 0.0016606 | 0.5221987 | 2.8435518 |
| ENSG00000100138 | SNU13 | K12845 | -4.1186661 | 4.353737494 | 15.690768 | 7.46E-05 | 0.0016606 | 0.4393939 | 2.9015152 |
| ENSG00000135341 | MAP3K7 | K04427 | -4.0636886 | 5.341890074 | 14.872958 | 0.000115 | 0.0022981 | 0.5264026 | 2.7953795 |
| ENSG00000170248 | PDCD6IP | K12200 | -4.0602355 | 5.527559376 | 14.028027 | 0.000180106 | 0.0027807 | 0.5040092 | 2.9415808 |
| ENSG00000118257 | NRP2 | K06819 | -4.0565912 | 5.736127427 | 12.842836 | 0.000338774 | 0.0043842 | 0.5359828 | 2.9795918 |
| ENSG00000140854 | KATNB1 | K18643 | -4.0561062 | 5.52426054 | 15.000935 | 0.000107458 | 0.0022944 | 0.4687023 | 2.8412214 |
| ENSG00000163092 | XIRP2 | - | -4.0430607 | 5.513089228 | 14.955183 | 0.000110095 | 0.0022981 | 0.6055227 | 2.9225134 |
| ENSG00000124507 | PACSIN1 | K20123 | -4.0415263 | 5.323413901 | 13.055361 | 0.000302417 | 0.0039674 | 0.6193694 | 2.9752252 |
| ENSG00000105649 | RAB3A | K07882 | -4.0400828 | 5.320987186 | 12.801261 | 0.000346386 | 0.0044776 | 0.5863636 | 2.9863636 |
| ENSG00000157554 | ERG | K09435 | -4.0136945 | 5.486368327 | 12.577037 | 0.000390515 | 0.0047009 | 0.5041152 | 2.9650206 |
| ENSG00000162695 | SLC30A7 | K14692 | -4.0065735 | 4.255327816 | 14.006534 | 0.000182176 | 0.0027807 | 0.3643617 | 3.0664894 |
| ENSG00000162341 | TPCN2 | K14077 | -4.0065731 | 4.255327816 | 14.005864 | 0.000182241 | 0.0027807 | 0.3829787 | 3.2300532 |
| ENSG00000160593 | JAML | - | -4.006573 | 4.255327816 | 14.005688 | 0.000182258 | 0.0027807 | 0.4543147 | 3.0329949 |
| ENSG00000163431 | LMOD1 | - | -4.006573 | 4.255327816 | 14.005556 | 0.000182271 | 0.0027807 | 0.5533333 | 2.7833333 |
| ENSG00000159110 | IFNAR2 | K05131 | -4.0065729 | 4.255327816 | 14.005512 | 0.000182276 | 0.0027807 | 0.5961165 | 2.9825243 |
| ENSG00000156990 | RPUSD3 | - | -4.0065728 | 4.255327816 | 14.005348 | 0.000182291 | 0.0027807 | 0.3618234 | 2.8461538 |
| ENSG00000158769 | F11R | K06089 | -4.0065728 | 4.255327816 | 14.005336 | 0.000182293 | 0.0027807 | 0.4882943 | 2.8361204 |
| ENSG00000154864 | PIEZO2 | - | -4.0065727 | 4.255327816 | 14.005104 | 0.000182315 | 0.0027807 | 0.4836154 | 3.2592726 |
| ENSG00000153989 | NUS1 | K19177 | -4.0065725 | 4.255327816 | 14.004861 | 0.000182339 | 0.0027807 | 0.4061433 | 3.0204778 |
| ENSG00000150687 | PRSS23 | K09627 | -4.0065724 | 4.255327816 | 14.004559 | 0.000182368 | 0.0027807 | 0.4229765 | 3.0417755 |
| ENSG00000152207 | CYSLTR2 | K04323 | -4.0065723 | 4.255327816 | 14.004506 | 0.000182373 | 0.0027807 | 0.3468208 | 3.3150289 |
| ENSG00000153786 | ZDHHC7 | K20029 | -4.0065723 | 4.255327816 | 14.004454 | 0.000182378 | 0.0027807 | 0.4 | 3.026087 |
| ENSG00000166401 | SERPINB8 | K13965 | -4.0065722 | 4.255327816 | 14.004322 | 0.000182391 | 0.0027807 | 0.5240642 | 3.0962567 |
| ENSG00000148484 | RSU1 | - | -4.006572 | 4.255327816 | 14.003897 | 0.000182432 | 0.0027807 | 0.5090253 | 3.0974729 |
| ENSG00000146376 | ARHGAP18 | K20639 | -4.0065717 | 4.255327816 | 14.003462 | 0.000182474 | 0.0027807 | 0.5475113 | 2.9803922 |
| ENSG00000145332 | KLHL8 | K10446 | -4.0065716 | 4.255327816 | 14.003307 | 0.000182489 | 0.0027807 | 0.466129 | 2.8548387 |
| ENSG00000168273 | SMIM4 | - | -4.0065715 | 4.255327816 | 14.003088 | 0.000182511 | 0.0027807 | 0.4 | 3.6571429 |
| ENSG00000144891 | AGTR1 | K04166 | -4.0065714 | 4.255327816 | 14.002847 | 0.000182534 | 0.0027807 | 0.3502538 | 3.322335 |
| ENSG00000144677 | CTDSPL | K15731 | -4.006571 | 4.255327816 | 14.002279 | 0.000182589 | 0.0027807 | 0.4818841 | 3.057971 |
| ENSG00000169504 | CLIC4 | K05024 | -4.0065708 | 4.255327816 | 14.001854 | 0.00018263 | 0.0027807 | 0.5256917 | 3.1027668 |
| ENSG00000144649 | FAM198A | - | -4.0065707 | 4.255327816 | 14.001711 | 0.000182644 | 0.0027807 | 0.4226087 | 2.8365217 |
| ENSG00000138074 | SLC5A6 | K14386 | -4.0065704 | 4.255327816 | 14.001142 | 0.0001827 | 0.0027807 | 0.3322835 | 2.9637795 |
| ENSG00000170458 | CD14 | K04391 | -4.0065701 | 4.255327816 | 14.00062 | 0.00018275 | 0.0027807 | 0.4 | 2.7066667 |
| ENSG00000137868 | STRA6 | - | -4.00657 | 4.255327816 | 14.000477 | 0.000182764 | 0.0027807 | 0.3427762 | 3.0056657 |
| ENSG00000135862 | LAMC1 | K05635 | -4.0065699 | 4.255327816 | 14.000317 | 0.00018278 | 0.0027807 | 0.5574891 | 2.6793039 |
| ENSG00000134917 | ADAMTS8 | K08623 | -4.0065699 | 4.255327816 | 14.000219 | 0.000182789 | 0.0027807 | 0.4420697 | 2.7322835 |
| ENSG00000135312 | HTR1B | K04153 | -4.0065698 | 4.255327816 | 14.00013 | 0.000182798 | 0.0027807 | 0.4 | 3.0974359 |
| ENSG00000134321 | RSAD2 | K15045 | -4.0065696 | 4.255327816 | 13.999716 | 0.000182838 | 0.0027807 | 0.4736842 | 3.2465374 |
| ENSG00000132849 | PATJ | K06092 | -4.0065695 | 4.255327816 | 13.99968 | 0.000182842 | 0.0027807 | 0.5491394 | 2.7556913 |
| ENSG00000172725 | CORO1B | K13886 | -4.0065694 | 4.255327816 | 13.999387 | 0.00018287 | 0.0027807 | 0.4805726 | 2.8650307 |
| ENSG00000132432 | SEC61G | K07342 | -4.0065693 | 4.255327816 | 13.999248 | 0.000182884 | 0.0027807 | 0.2941176 | 3.2205882 |
| ENSG00000131669 | NINJ1 | - | -4.006569 | 4.255327816 | 13.998816 | 0.000182926 | 0.0027807 | 0.3618421 | 2.9342105 |
| ENSG00000130684 | ZNF337 | K09228 | -4.0065689 | 4.255327816 | 13.998623 | 0.000182945 | 0.0027807 | 0.436751 | 3.0279627 |
| ENSG00000173442 | EHBP1L1 | - | -4.0065687 | 4.255327816 | 13.998154 | 0.00018299 | 0.0027807 | 0.5357846 | 2.5265923 |
| ENSG00000128641 | MYO1B | K10356 | -4.0065685 | 4.255327816 | 13.997935 | 0.000183012 | 0.0027807 | 0.4859155 | 3.2059859 |
| ENSG00000126016 | AMOT | K16819 | -4.0065681 | 4.255327816 | 13.997244 | 0.000183079 | 0.0027807 | 0.4990775 | 2.6937269 |
| ENSG00000125810 | CD93 | K06702 | -4.0065681 | 4.255327816 | 13.997175 | 0.000183085 | 0.0027807 | 0.4570552 | 2.5030675 |
| ENSG00000125257 | ABCC4 | K05673 | -4.006568 | 4.255327816 | 13.997107 | 0.000183092 | 0.0027807 | 0.44 | 3.1207547 |
| ENSG00000122786 | CALD1 | K12327 | -4.006568 | 4.255327816 | 13.997038 | 0.000183099 | 0.0027807 | 0.7137453 | 2.9419924 |
| ENSG00000176472 | ZNF575 | - | -4.0065679 | 4.255327816 | 13.996922 | 0.00018311 | 0.0027807 | 0.3284884 | 2.747093 |
| ENSG00000117335 | CD46 | K04007 | -4.0065676 | 4.255327816 | 13.996264 | 0.000183174 | 0.0027807 | 0.4536341 | 3.0150376 |
| ENSG00000178031 | ADAMTSL1 | - | -4.0065672 | 4.255327816 | 13.99569 | 0.00018323 | 0.0027807 | 0.4517594 | 2.7485812 |
| ENSG00000106991 | ENG | K06526 | -4.0065671 | 4.255327816 | 13.995548 | 0.000183244 | 0.0027807 | 0.4346505 | 2.7492401 |
| ENSG00000105835 | NAMPT | K03462 | -4.006567 | 4.255327816 | 13.995312 | 0.000183267 | 0.0027807 | 0.5152749 | 3.114053 |
| ENSG00000105655 | ISYNA1 | K01858 | -4.0065665 | 4.255327816 | 13.994534 | 0.000183343 | 0.0027807 | 0.4498208 | 2.8512545 |
| ENSG00000178605 | GTPBP6 | - | -4.0065665 | 4.255327816 | 13.994458 | 0.00018335 | 0.0027807 | 0.4108527 | 2.8488372 |
| ENSG00000105509 | HAS1 | K00752 | -4.0065661 | 4.255327816 | 13.993756 | 0.000183419 | 0.0027807 | 0.3452991 | 3.0683761 |
| ENSG00000179144 | GIMAP7 | - | -4.0065658 | 4.255327816 | 13.993226 | 0.00018347 | 0.0027807 | 0.5666667 | 3.05 |
| ENSG00000104368 | PLAT | K01343 | -4.0065656 | 4.255327816 | 13.992978 | 0.000183495 | 0.0027807 | 0.4626335 | 2.8451957 |
| ENSG00000104142 | VPS18 | K20181 | -4.0065652 | 4.255327816 | 13.9922 | 0.000183571 | 0.0027807 | 0.4727646 | 3.053443 |
| ENSG00000179855 | GIPC3 | K20056 | -4.0065651 | 4.255327816 | 13.991995 | 0.000183591 | 0.0027807 | 0.4775641 | 2.7660256 |
| ENSG00000103966 | EHD4 | K12477 | -4.0065647 | 4.255327816 | 13.991422 | 0.000183647 | 0.0027807 | 0.4879852 | 3.0702403 |
| ENSG00000102755 | FLT1 | K05096 | -4.0065645 | 4.255327816 | 13.991001 | 0.000183688 | 0.0027807 | 0.5037369 | 2.9880419 |
| ENSG00000179862 | CITED4 | - | -4.0065644 | 4.255327816 | 13.990764 | 0.000183711 | 0.0027807 | 0.298913 | 2.548913 |
| ENSG00000101052 | IFT52 | K19681 | -4.0065642 | 4.255327816 | 13.990582 | 0.000183729 | 0.0027807 | 0.5423341 | 3.1121281 |
| ENSG00000090339 | ICAM1 | K06490 | -4.006564 | 4.255327816 | 13.990163 | 0.00018377 | 0.0027807 | 0.4605263 | 2.7838346 |
| ENSG00000068001 | HYAL2 | K01197 | -4.006564 | 4.255327816 | 13.990141 | 0.000183772 | 0.0027807 | 0.4249471 | 3.0993658 |
| ENSG00000047365 | ARAP2 | K18440 | -4.0065637 | 4.255327816 | 13.989717 | 0.000183813 | 0.0027807 | 0.5387324 | 3.0264085 |
| ENSG00000185043 | CIB1 | K17259 | -4.0065636 | 4.255327816 | 13.989533 | 0.000183831 | 0.0027807 | 0.6017316 | 2.9393939 |
| ENSG00000186891 | TNFRSF18 | K05154 | -4.0065629 | 4.255327816 | 13.988303 | 0.000183951 | 0.0027807 | 0.345098 | 2.3764706 |
| ENSG00000187244 | BCAM | K06578 | -4.0065622 | 4.255327816 | 13.987073 | 0.000184072 | 0.0027807 | 0.4745223 | 2.6528662 |
| ENSG00000104856 | RELB | K09253 | -3.9955379 | 6.383802153 | 14.372326 | 0.000149991 | 0.0026567 | 0.4369603 | 2.7202073 |
| ENSG00000154914 | USP43 | K11856 | -3.9686248 | 5.260355971 | 13.605351 | 0.000225542 | 0.0030389 | 0.4363313 | 2.7960819 |
| ENSG00000144642 | RBMS3 | - | -3.9647105 | 5.847071382 | 14.780756 | 0.000120762 | 0.0023832 | 0.5034325 | 2.791762 |
| ENSG00000090674 | MCOLN1 | K04992 | -3.9644615 | 5.256830654 | 13.591363 | 0.000227229 | 0.003058 | 0.4189655 | 3.0793103 |
| ENSG00000151090 | THRB | K08362 | -3.9564442 | 4.212848893 | 14.901669 | 0.000113263 | 0.0022981 | 0.512605 | 3.0609244 |
| ENSG00000139926 | FRMD6 | K16822 | -3.9564439 | 4.212848893 | 14.901169 | 0.000113293 | 0.0022981 | 0.5369775 | 3.1061093 |
| ENSG00000095585 | BLNK | K07371 | -3.9564435 | 4.212848893 | 14.900528 | 0.000113331 | 0.0022981 | 0.5328947 | 2.8508772 |
| ENSG00000160695 | VPS11 | K20179 | -3.9513616 | 5.835683377 | 14.730115 | 0.000124049 | 0.0024148 | 0.5047821 | 3.0924548 |
| ENSG00000110436 | SLC1A2 | K05613 | -3.948202 | 5.430622929 | 12.345557 | 0.000442037 | 0.0050583 | 0.3850174 | 2.8623693 |
| ENSG00000188828 | GLRA4 | K05271 | -3.9477302 | 4.205608597 | 14.880853 | 0.00011452 | 0.0022981 | 0.4532374 | 3.1606715 |
| ENSG00000186439 | TRDN | - | -3.9477298 | 4.205608597 | 14.880153 | 0.000114562 | 0.0022981 | 0.5747599 | 2.9067215 |
| ENSG00000175806 | MSRA | K07304 | -3.9477294 | 4.205608597 | 14.879487 | 0.000114602 | 0.0022981 | 0.4638298 | 2.9191489 |
| ENSG00000182628 | SKA2 | - | -3.9477294 | 4.205608597 | 14.879454 | 0.000114604 | 0.0022981 | 0.5950413 | 3.2066116 |
| ENSG00000163701 | IL17RE | K05168 | -3.9477291 | 4.205608597 | 14.878983 | 0.000114633 | 0.0022981 | 0.3542857 | 3.0114286 |
| ENSG00000144504 | ANKMY1 | - | -3.9477287 | 4.205608597 | 14.878347 | 0.000114672 | 0.0022981 | 0.4825243 | 2.9398058 |
| ENSG00000169884 | WNT10B | K01357 | -3.9477286 | 4.205608597 | 14.878271 | 0.000114676 | 0.0022981 | 0.3676093 | 2.748072 |
| ENSG00000143258 | USP21 | K21634 | -3.9477281 | 4.205608597 | 14.877397 | 0.00011473 | 0.0022981 | 0.4 | 2.8725664 |
| ENSG00000141577 | CEP131 | K16540 | -3.9477276 | 4.205608597 | 14.876564 | 0.00011478 | 0.0022981 | 0.5614035 | 2.7885503 |
| ENSG00000140365 | COMMD4 | - | -3.9477274 | 4.205608597 | 14.876209 | 0.000114802 | 0.0022981 | 0.4218009 | 2.7061611 |
| ENSG00000138759 | FRAS1 | - | -3.947727 | 4.205608597 | 14.875587 | 0.00011484 | 0.0022981 | 0.5097208 | 2.8270189 |
| ENSG00000135114 | OASL | K14608 | -3.9477267 | 4.205608597 | 14.87506 | 0.000114872 | 0.0022981 | 0.4824903 | 3.1828794 |
| ENSG00000130545 | CRB3 | K06090 | -3.9477264 | 4.205608597 | 14.874534 | 0.000114904 | 0.0022981 | 0.4227642 | 2.7073171 |
| ENSG00000130529 | TRPM4 | K04979 | -3.9477259 | 4.205608597 | 14.873712 | 0.000114954 | 0.0022981 | 0.392916 | 2.9522241 |
| ENSG00000130037 | KCNA5 | K04878 | -3.9477254 | 4.205608597 | 14.872886 | 0.000115004 | 0.0022981 | 0.4274062 | 2.9070147 |
| ENSG00000129038 | LOXL1 | K14678 | -3.947725 | 4.205608597 | 14.872239 | 0.000115044 | 0.0022981 | 0.4703833 | 2.8432056 |
| ENSG00000128891 | C15orf57 | - | -3.9477246 | 4.205608597 | 14.871592 | 0.000115083 | 0.0022981 | 0.5714286 | 2.8472906 |
| ENSG00000124839 | RAB17 | K07909 | -3.9477242 | 4.205608597 | 14.870945 | 0.000115123 | 0.0022981 | 0.4622642 | 2.9103774 |
| ENSG00000105204 | DYRK1B | K08825 | -3.9477236 | 4.205608597 | 14.869876 | 0.000115188 | 0.0022981 | 0.427663 | 2.8696343 |
| ENSG00000072195 | LOC100996693 | - | -3.9477234 | 4.205608597 | 14.86951 | 0.00011521 | 0.0022981 | 0.4527089 | 2.7239057 |
| ENSG00000067113 | PLPP1 | K01080 | -3.9477231 | 4.205608597 | 14.869144 | 0.000115233 | 0.0022981 | 0.4350877 | 3.1298246 |
| ENSG00000065413 | ANKRD44 | K15503 | -3.9477229 | 4.205608597 | 14.868778 | 0.000115255 | 0.0022981 | 0.4592145 | 2.7119839 |
| ENSG00000051523 | CYBA | K08009 | -3.9477224 | 4.205608597 | 14.867822 | 0.000115313 | 0.0022981 | 0.2619048 | 2.9904762 |
| ENSG00000040199 | PHLPP2 | K16340 | -3.9477217 | 4.205608597 | 14.866769 | 0.000115378 | 0.0022981 | 0.5162003 | 2.8343152 |
| ENSG00000022567 | SLC45A4 | K15378 | -3.9477211 | 4.205608597 | 14.865715 | 0.000115442 | 0.0022981 | 0.4504951 | 2.9158416 |
| ENSG00000006016 | CRLF1 | - | -3.947721 | 4.205608597 | 14.865621 | 0.000115448 | 0.0022981 | 0.4028436 | 2.8388626 |
| ENSG00000174938 | SEZ6L2 | - | -3.9471144 | 5.832107628 | 13.713854 | 0.000212878 | 0.0029058 | 0.468039 | 2.751896 |
| ENSG00000112759 | SLC29A1 | K15014 | -3.9442055 | 5.239708752 | 13.521427 | 0.000235855 | 0.0031666 | 0.3399123 | 3.125 |
| ENSG00000064102 | ASUN | - | -3.9426 | 4.202130671 | 13.81243 | 0.000201995 | 0.0029001 | 0.5396601 | 2.9291785 |
| ENSG00000103490 | PYCARD | K12799 | -3.9425997 | 4.202130671 | 13.811889 | 0.000202054 | 0.0029001 | 0.4615385 | 2.9282051 |
| ENSG00000134627 | PIWIL4 | K02156 | -3.9425993 | 4.202130671 | 13.811349 | 0.000202112 | 0.0029001 | 0.4730047 | 3.0328639 |
| ENSG00000169313 | P2RY12 | K04298 | -3.942599 | 4.202130671 | 13.810809 | 0.00020217 | 0.0029001 | 0.3888889 | 3.3567251 |
| ENSG00000172508 | CARNS1 | K14755 | -3.9425987 | 4.202130671 | 13.810269 | 0.000202228 | 0.0029001 | 0.3757895 | 2.7136842 |
| ENSG00000141579 | ZNF750 | - | -3.9389914 | 5.23567648 | 14.487835 | 0.000141068 | 0.0026567 | 0.4716459 | 2.6832642 |
| ENSG00000113361 | CDH6 | K06798 | -3.9389908 | 5.23567648 | 14.489049 | 0.000140977 | 0.0026567 | 0.5835443 | 2.9341772 |
| ENSG00000106268 | NUDT1 | K17816 | -3.9389904 | 5.23567648 | 14.489812 | 0.00014092 | 0.0026567 | 0.4972067 | 3.0670391 |
| ENSG00000006118 | TMEM132A | K17599 | -3.9389904 | 5.23567648 | 14.489824 | 0.000140919 | 0.0026567 | 0.4267578 | 2.7480469 |
| ENSG00000101638 | ST8SIA5 | K03369 | -3.9309685 | 5.836257724 | 13.102804 | 0.000294854 | 0.0038815 | 0.4490291 | 3.2475728 |
| ENSG00000165434 | PGM2L1 | K11809 | -3.9286259 | 5.226573389 | 13.478652 | 0.000241293 | 0.0032283 | 0.4935691 | 3.0691318 |
| ENSG00000120053 | GOT1 | K14454 | -3.9286257 | 5.226573389 | 13.479155 | 0.000241228 | 0.0032283 | 0.4624697 | 3.031477 |
| ENSG00000164211 | STARD4 | - | -3.9275892 | 5.625826013 | 14.294519 | 0.000156319 | 0.0026567 | 0.5268293 | 3.1170732 |
| ENSG00000185666 | SYN3 | K19941 | -3.9246417 | 4.187259549 | 13.763864 | 0.000207285 | 0.0029001 | 0.4655172 | 2.7758621 |
| ENSG00000184702 | SEPT5-GP1BB | - | -3.9246413 | 4.187259549 | 13.763155 | 0.000207364 | 0.0029001 | 0.5291005 | 3.0978836 |
| ENSG00000184388 | PABPC1L2B | K13126 | -3.9246408 | 4.187259549 | 13.762446 | 0.000207442 | 0.0029001 | 0.475 | 3.06 |
| ENSG00000184185 | KCNJ12 | K05005 | -3.9246404 | 4.187259549 | 13.761737 | 0.00020752 | 0.0029001 | 0.4711316 | 3.0161663 |
| ENSG00000183036 | PCP4 | - | -3.92464 | 4.187259549 | 13.761029 | 0.000207598 | 0.0029001 | 0.6290323 | 2.5645161 |
| ENSG00000182346 | DAOA | - | -3.9246395 | 4.187259549 | 13.76032 | 0.000207677 | 0.0029001 | 0.5620915 | 3.1764706 |
| ENSG00000181027 | FKRP | K19873 | -3.9246391 | 4.187259549 | 13.759611 | 0.000207755 | 0.0029001 | 0.379798 | 2.9515152 |
| ENSG00000176723 | ZNF843 | - | -3.9246388 | 4.187259549 | 13.759106 | 0.000207811 | 0.0029001 | 0.3362069 | 2.6293103 |
| ENSG00000174807 | CD248 | K06706 | -3.9246386 | 4.187259549 | 13.758867 | 0.000207837 | 0.0029001 | 0.4240423 | 2.673712 |
| ENSG00000174775 | HRAS | K02833 | -3.9246383 | 4.187259549 | 13.758435 | 0.000207885 | 0.0029001 | 0.5555556 | 2.8994709 |
| ENSG00000171992 | SYNPO | K21112 | -3.9246381 | 4.187259549 | 13.758003 | 0.000207933 | 0.0029001 | 0.482239 | 2.6404736 |
| ENSG00000168314 | MOBP | - | -3.9246376 | 4.187259549 | 13.757277 | 0.000208013 | 0.0029001 | 0.3786408 | 2.8980583 |
| ENSG00000167614 | TTYH1 | - | -3.9246372 | 4.187259549 | 13.756551 | 0.000208094 | 0.0029001 | 0.3782609 | 2.876087 |
| ENSG00000166797 | FAM96A | - | -3.9246369 | 4.187259549 | 13.756187 | 0.000208134 | 0.0029001 | 0.55625 | 3.03125 |
| ENSG00000164100 | NDST3 | K02578 | -3.9246364 | 4.187259549 | 13.755271 | 0.000208236 | 0.0029001 | 0.4604811 | 3.2863688 |
| ENSG00000163630 | SYNPR | - | -3.9246358 | 4.187259549 | 13.754355 | 0.000208337 | 0.0029001 | 0.4526316 | 2.9964912 |
| ENSG00000163618 | CADPS | K19933 | -3.9246352 | 4.187259549 | 13.75344 | 0.000208439 | 0.0029001 | 0.5269771 | 2.9704361 |
| ENSG00000162706 | CADM3 | K06780 | -3.9246349 | 4.187259549 | 13.75285 | 0.000208504 | 0.0029001 | 0.5162037 | 2.7916667 |
| ENSG00000162441 | LZIC | - | -3.9246343 | 4.187259549 | 13.751895 | 0.00020861 | 0.0029001 | 0.6157895 | 2.9052632 |
| ENSG00000160752 | FDPS | K00787 | -3.9246337 | 4.187259549 | 13.75094 | 0.000208716 | 0.0029001 | 0.477327 | 3.202864 |
| ENSG00000160460 | SPTBN4 | K06115 | -3.9246331 | 4.187259549 | 13.749985 | 0.000208822 | 0.0029001 | 0.5035101 | 2.8958658 |
| ENSG00000157103 | SLC6A1 | K05034 | -3.9246324 | 4.187259549 | 13.748859 | 0.000208948 | 0.0029001 | 0.3656093 | 3.2036728 |
| ENSG00000156113 | KCNMA1 | K04936 | -3.924632 | 4.187259549 | 13.748187 | 0.000209022 | 0.0029001 | 0.4720497 | 2.9332298 |
| ENSG00000154813 | DPH3 | K15455 | -3.9246315 | 4.187259549 | 13.747336 | 0.000209117 | 0.0029001 | 0.6829268 | 2.9390244 |
| ENSG00000154096 | THY1 | K06514 | -3.9246311 | 4.187259549 | 13.746814 | 0.000209175 | 0.0029001 | 0.4606061 | 2.8606061 |
| ENSG00000146242 | TPBG | - | -3.9246308 | 4.187259549 | 13.746329 | 0.000209229 | 0.0029001 | 0.4214286 | 2.852381 |
| ENSG00000145495 | 6-Mar | K10661 | -3.9246301 | 4.187259549 | 13.745165 | 0.000209359 | 0.0029001 | 0.3736264 | 3.1714286 |
| ENSG00000144834 | TAGLN3 | K20526 | -3.9246294 | 4.187259549 | 13.744002 | 0.000209489 | 0.0029001 | 0.4751131 | 2.9411765 |
| ENSG00000144115 | THNSL2 | - | -3.9246288 | 4.187259549 | 13.74312 | 0.000209587 | 0.0029001 | 0.464876 | 2.9545455 |
| ENSG00000142634 | EFHD2 | - | -3.9246283 | 4.187259549 | 13.742237 | 0.000209686 | 0.0029001 | 0.5416667 | 2.8458333 |
| ENSG00000142544 | CTU1 | K14168 | -3.9246277 | 4.187259549 | 13.741305 | 0.00020979 | 0.0029001 | 0.3017241 | 2.5431034 |
| ENSG00000139874 | SSTR1 | K04217 | -3.9246273 | 4.187259549 | 13.740662 | 0.000209861 | 0.0029001 | 0.3657289 | 2.9130435 |
| ENSG00000139287 | TPH2 | K00502 | -3.924627 | 4.187259549 | 13.74024 | 0.000209909 | 0.0029001 | 0.5265306 | 3.0979592 |
| ENSG00000137252 | HCRTR2 | K04239 | -3.9246267 | 4.187259549 | 13.73975 | 0.000209963 | 0.0029001 | 0.3963964 | 3.1846847 |
| ENSG00000136319 | TTC5 | - | -3.9246261 | 4.187259549 | 13.73877 | 0.000210073 | 0.0029001 | 0.5136364 | 2.8909091 |
| ENSG00000135845 | PIGC | K03859 | -3.9246256 | 4.187259549 | 13.737896 | 0.000210171 | 0.0029001 | 0.3737374 | 3.2491582 |
| ENSG00000133703 | KRAS | K07827 | -3.9246254 | 4.187259549 | 13.737623 | 0.000210201 | 0.0029001 | 0.5449735 | 3.042328 |
| ENSG00000132702 | HAPLN2 | K06851 | -3.9246252 | 4.187259549 | 13.737349 | 0.000210232 | 0.0029001 | 0.3735294 | 2.9558824 |
| ENSG00000130940 | CASZ1 | - | -3.9246248 | 4.187259549 | 13.736702 | 0.000210304 | 0.0029001 | 0.4855031 | 2.6850483 |
| ENSG00000130558 | OLFM1 | - | -3.9246244 | 4.187259549 | 13.736055 | 0.000210377 | 0.0029001 | 0.5360825 | 3.0309278 |
| ENSG00000128610 | FEZF1 | - | -3.9246239 | 4.187259549 | 13.73514 | 0.000210479 | 0.0029001 | 0.3431579 | 2.8694737 |
| ENSG00000127561 | SYNGR3 | - | -3.9246233 | 4.187259549 | 13.734212 | 0.000210583 | 0.0029001 | 0.2008368 | 2.6276151 |
| ENSG00000122012 | SV2C | K06258 | -3.9246227 | 4.187259549 | 13.733241 | 0.000210692 | 0.0029001 | 0.4814305 | 3.1210454 |
| ENSG00000119682 | AREL1 | - | -3.9246221 | 4.187259549 | 13.73227 | 0.000210801 | 0.0029001 | 0.4811665 | 3.1567436 |
| ENSG00000118160 | SLC8A2 | K05849 | -3.9246216 | 4.187259549 | 13.731543 | 0.000210883 | 0.0029001 | 0.4375679 | 2.9022801 |
| ENSG00000116690 | PRG4 | - | -3.9246214 | 4.187259549 | 13.731144 | 0.000210928 | 0.0029001 | 0.5320513 | 2.7172365 |
| ENSG00000115507 | OTX1 | K09326 | -3.9246208 | 4.187259549 | 13.730253 | 0.000211028 | 0.0029001 | 0.4152542 | 2.5649718 |
| ENSG00000115464 | USP34 | K11853 | -3.9246203 | 4.187259549 | 13.729362 | 0.000211128 | 0.0029001 | 0.5279188 | 3.0276368 |
| ENSG00000113302 | IL12B | K05425 | -3.9246201 | 4.187259549 | 13.729092 | 0.000211158 | 0.0029001 | 0.554878 | 3.0182927 |
| ENSG00000112218 | GPR63 | K04321 | -3.9246194 | 4.187259549 | 13.727951 | 0.000211287 | 0.0029001 | 0.3198091 | 3.3389021 |
| ENSG00000106868 | SUSD1 | - | -3.9246188 | 4.187259549 | 13.727032 | 0.00021139 | 0.0029001 | 0.4834875 | 2.8731836 |
| ENSG00000105808 | RASA4 | K17630 | -3.9246182 | 4.187259549 | 13.726057 | 0.0002115 | 0.0029001 | 0.4719801 | 2.9800747 |
| ENSG00000102271 | KLHL4 | K10442 | -3.9246176 | 4.187259549 | 13.725082 | 0.000211609 | 0.0029001 | 0.4847222 | 2.9222222 |
| ENSG00000101438 | SLC32A1 | K15015 | -3.924617 | 4.187259549 | 13.724108 | 0.000211719 | 0.0029001 | 0.3580952 | 3 |
| ENSG00000100997 | ABHD12 | K13704 | -3.9246166 | 4.187259549 | 13.723393 | 0.0002118 | 0.0029001 | 0.4009901 | 3.1089109 |
| ENSG00000100523 | DDHD1 | K13619 | -3.9246161 | 4.187259549 | 13.722679 | 0.00021188 | 0.0029001 | 0.5311111 | 2.8988889 |
| ENSG00000100285 | NEFH | K04574 | -3.9246157 | 4.187259549 | 13.721965 | 0.000211961 | 0.0029001 | 0.6029412 | 2.7176471 |
| ENSG00000077522 | ACTN2 | K21073 | -3.9246155 | 4.187259549 | 13.721577 | 0.000212005 | 0.0029001 | 0.5592841 | 3.098434 |
| ENSG00000076826 | CAMSAP3 | K17493 | -3.9246152 | 4.187259549 | 13.721189 | 0.000212049 | 0.0029001 | 0.4435737 | 2.7405956 |
| ENSG00000067715 | SYT1 | K15290 | -3.924615 | 4.187259549 | 13.720801 | 0.000212092 | 0.0029001 | 0.4834123 | 3.0734597 |
| ENSG00000058063 | ATP11B | K01530 | -3.9246149 | 4.187259549 | 13.720627 | 0.000212112 | 0.0029001 | 0.4740867 | 3.1537808 |
| ENSG00000038427 | VCAN | K06793 | -3.9246148 | 4.187259549 | 13.720452 | 0.000212132 | 0.0029001 | 0.6439929 | 2.7894582 |
| ENSG00000017483 | SLC38A5 | K14992 | -3.9246146 | 4.187259549 | 13.720278 | 0.000212151 | 0.0029001 | 0.309322 | 3.0360169 |
| ENSG00000008056 | SYN1 | K19941 | -3.9246143 | 4.187259549 | 13.719764 | 0.000212209 | 0.0029001 | 0.3971631 | 2.6042553 |
| ENSG00000107779 | BMPR1A | K04673 | -3.9170326 | 5.2145291 | 11.496112 | 0.000697419 | 0.0074832 | 0.4699248 | 2.9981203 |
| ENSG00000100490 | CDKL1 | K08824 | -3.9170326 | 5.2145291 | 11.496223 | 0.000697377 | 0.0074832 | 0.452514 | 3.2821229 |
| ENSG00000142173 | COL6A2 | K06238 | -3.9170324 | 5.2145291 | 11.496579 | 0.000697244 | 0.0074832 | 0.4553484 | 2.6280667 |
| ENSG00000143226 | FCGR2A | K06472 | -3.9153186 | 4.17880842 | 14.746589 | 0.00012297 | 0.0024024 | 0.4921136 | 2.8611987 |
| ENSG00000158220 | ESYT3 | - | -3.9153185 | 4.17880842 | 14.746542 | 0.000122973 | 0.0024024 | 0.4525959 | 3.0507901 |
| ENSG00000168824 | NSG1 | - | -3.9153185 | 4.17880842 | 14.746494 | 0.000122976 | 0.0024024 | 0.5243243 | 3.1135135 |
| ENSG00000136040 | PLXNC1 | K06572 | -3.9153185 | 4.17880842 | 14.746437 | 0.00012298 | 0.0024024 | 0.4917092 | 2.9642857 |
| ENSG00000175826 | CTDNEP1 | K17617 | -3.9153184 | 4.17880842 | 14.746356 | 0.000122985 | 0.0024024 | 0.4057377 | 3.2991803 |
| ENSG00000082556 | OPRK1 | K04214 | -3.9153184 | 4.17880842 | 14.746284 | 0.00012299 | 0.0024024 | 0.3921053 | 3.1289474 |
| ENSG00000168081 | PNOC | - | -3.8956086 | 4.169558698 | 12.943513 | 0.000321032 | 0.0041829 | 0.4659091 | 3.0170455 |
| ENSG00000131023 | LATS1 | K08791 | -3.8935421 | 5.78585394 | 14.419474 | 0.000146282 | 0.0026567 | 0.5115044 | 2.9168142 |
| ENSG00000175455 | CCDC14 | K16541 | -3.8913743 | 5.594841248 | 12.208917 | 0.000475616 | 0.0053781 | 0.5635965 | 2.7839912 |
| ENSG00000149451 | ADAM33 | K08616 | -3.8773555 | 5.370782806 | 13.093275 | 0.000296358 | 0.0038968 | 0.3763838 | 2.6654367 |
| ENSG00000122733 | PHF24 | - | -3.8585467 | 5.979638854 | 12.323484 | 0.000447295 | 0.0051032 | 0.5375 | 2.87 |
| ENSG00000154305 | MIA3 | - | -3.8583226 | 5.355640206 | 12.272559 | 0.000459667 | 0.0052391 | 0.6261143 | 2.8542213 |
| ENSG00000138162 | TACC2 | K14282 | -3.8578669 | 5.35464797 | 13.979142 | 0.00018485 | 0.0027888 | 0.5440977 | 2.5054274 |
| ENSG00000161609 | CCDC155 | - | -3.856821 | 5.354519049 | 13.080109 | 0.000298448 | 0.0039198 | 0.5551601 | 2.8558719 |
| ENSG00000163170 | BOLA3 | - | -3.8537953 | 5.351229026 | 13.965388 | 0.000186207 | 0.0028056 | 0.4018692 | 3 |
| ENSG00000058404 | CAMK2B | K04515 | -3.8503053 | 5.348919621 | 12.247315 | 0.000465928 | 0.0052842 | 0.4504505 | 2.8183183 |
| ENSG00000119771 | KLHL29 | K10465 | -3.8503053 | 5.348919621 | 12.247381 | 0.000465912 | 0.0052842 | 0.4297143 | 2.776 |
| ENSG00000065559 | MAP2K4 | K04430 | -3.8503052 | 5.348919621 | 12.247637 | 0.000465848 | 0.0052842 | 0.4682927 | 2.9341463 |
| ENSG00000143630 | HCN3 | K04956 | -3.8503052 | 5.348919621 | 12.247651 | 0.000465844 | 0.0052842 | 0.369509 | 2.9870801 |
| ENSG00000113971 | NPHP3 | K19360 | -3.8461227 | 5.85236036 | 13.703837 | 0.000214017 | 0.0029179 | 0.5105263 | 3.043609 |
| ENSG00000178235 | SLITRK1 | - | -3.8394341 | 5.8463949 | 12.772476 | 0.000351757 | 0.0045419 | 0.4798851 | 2.9698276 |
| ENSG00000117154 | IGSF21 | - | -3.8383596 | 5.339059184 | 13.019519 | 0.000308261 | 0.0040257 | 0.4646681 | 2.9100642 |
| ENSG00000152932 | RAB3C | K07883 | -3.8320739 | 5.143255619 | 11.219557 | 0.000809398 | 0.0075822 | 0.5770925 | 3.0264317 |
| ENSG00000177301 | KCNA2 | K04875 | -3.8320739 | 5.143255619 | 11.21957 | 0.000809392 | 0.0075822 | 0.511022 | 3.1442886 |
| ENSG00000069020 | MAST4 | K08789 | -3.831242 | 5.142895278 | 12.075659 | 0.000510844 | 0.0057369 | 0.5005719 | 2.6778498 |
| ENSG00000115977 | AAK1 | K08853 | -3.8084426 | 6.223581145 | 12.336105 | 0.000444281 | 0.0050789 | 0.4973985 | 2.7377732 |
| ENSG00000101000 | PROCR | K06557 | -3.8023035 | 5.518844953 | 11.651967 | 0.000641347 | 0.0069902 | 0.4453782 | 3.0252101 |
| ENSG00000151366 | NDUFC2 | K03968 | -3.7995896 | 6.006355276 | 13.867922 | 0.000196117 | 0.0029001 | 0.4201681 | 3.5294118 |
| ENSG00000188647 | PTAR1 | K14137 | -3.7823947 | 5.798107729 | 13.370073 | 0.000255671 | 0.0033969 | 0.4975124 | 3.141791 |
| ENSG00000168913 | ENHO | - | -3.7733803 | 5.283546802 | 12.749492 | 0.000356106 | 0.0045929 | 0.3552632 | 2.6315789 |
| ENSG00000107937 | GTPBP4 | K06943 | -3.7531322 | 5.266737054 | 12.681376 | 0.000369316 | 0.0047009 | 0.5015773 | 3.1246057 |
| ENSG00000187239 | FNBP1 | K20121 | -3.7531322 | 5.266737054 | 12.681609 | 0.00036927 | 0.0047009 | 0.6126418 | 3.0372771 |
| ENSG00000151067 | CACNA1C | K04850 | -3.7523515 | 5.77152699 | 11.224902 | 0.00080707 | 0.0075727 | 0.4459031 | 3.0149389 |
| ENSG00000115459 | ELMOD3 | - | -3.7521847 | 5.68416528 | 11.380377 | 0.00074224 | 0.0074949 | 0.4322251 | 3.0971867 |
| ENSG00000087253 | LPCAT2 | K13510 | -3.7480608 | 5.262780021 | 13.625502 | 0.000223134 | 0.0030099 | 0.4264706 | 3.0147059 |
| ENSG00000123159 | GIPC1 | K20056 | -3.7480607 | 5.262780021 | 13.625577 | 0.000223125 | 0.0030099 | 0.4264264 | 2.7987988 |
| ENSG00000164951 | PDP1 | K01102 | -3.7480607 | 5.262780021 | 13.625652 | 0.000223116 | 0.0030099 | 0.4857651 | 3.0355872 |
| ENSG00000091009 | RBM27 | K13193 | -3.7419758 | 5.257497135 | 12.64663 | 0.000376243 | 0.0047009 | 0.5179245 | 2.8801887 |
| ENSG00000167986 | DDB1 | K10610 | -3.7382806 | 5.878580223 | 12.625039 | 0.000380614 | 0.0047009 | 0.5114035 | 2.9421053 |
| ENSG00000143314 | MRPL24 | K02895 | -3.7375644 | 5.253843498 | 12.629472 | 0.000379712 | 0.0047009 | 0.4537037 | 3.1481481 |
| ENSG00000176697 | BDNF | K04355 | -3.7375641 | 5.253843498 | 12.630398 | 0.000379524 | 0.0047009 | 0.4528875 | 2.9452888 |
| ENSG00000066629 | EML1 | K18595 | -3.7256842 | 5.242054295 | 10.701739 | 0.001070349 | 0.0095882 | 0.5023981 | 2.8345324 |
| ENSG00000174456 | C12orf76 | - | -3.7256841 | 5.242054295 | 10.701854 | 0.001070282 | 0.0095882 | 0.3925926 | 2.837037 |
| ENSG00000108518 | PFN1 | K05759 | -3.7152096 | 5.046621124 | 12.514996 | 0.000403699 | 0.0047009 | 0.369697 | 2.5333333 |
| ENSG00000043143 | JADE2 | - | -3.7106861 | 4.010570214 | 14.338345 | 0.000152722 | 0.0026567 | 0.5191847 | 2.7709832 |
| ENSG00000044446 | PHKA2 | K07190 | -3.710686 | 4.010570214 | 14.338094 | 0.000152743 | 0.0026567 | 0.4898785 | 2.9716599 |
| ENSG00000060558 | GNA15 | K04637 | -3.7106858 | 4.010570214 | 14.337844 | 0.000152763 | 0.0026567 | 0.4973262 | 3.1604278 |
| ENSG00000069974 | RAB27A | K07885 | -3.7106856 | 4.010570214 | 14.337593 | 0.000152783 | 0.0026567 | 0.5339367 | 2.9411765 |
| ENSG00000077150 | NFKB2 | K04469 | -3.7106854 | 4.010570214 | 14.337343 | 0.000152804 | 0.0026567 | 0.4655556 | 2.7044444 |
| ENSG00000108061 | SHOC2 | K19613 | -3.7106854 | 4.010570214 | 14.337311 | 0.000152806 | 0.0026567 | 0.5034364 | 2.9209622 |
| ENSG00000083828 | ZNF586 | - | -3.7106852 | 4.010570214 | 14.337093 | 0.000152824 | 0.0026567 | 0.4875622 | 2.9552239 |
| ENSG00000090376 | IRAK3 | K04732 | -3.7106851 | 4.010570214 | 14.336843 | 0.000152844 | 0.0026567 | 0.4916107 | 3.0536913 |
| ENSG00000116299 | KIAA1324 | - | -3.710685 | 4.010570214 | 14.336737 | 0.000152853 | 0.0026567 | 0.5034551 | 2.810464 |
| ENSG00000120156 | TEK | K05121 | -3.7106846 | 4.010570214 | 14.336163 | 0.000152899 | 0.0026567 | 0.4661922 | 2.9510676 |
| ENSG00000120860 | CCDC53 | K18463 | -3.7106842 | 4.010570214 | 14.335589 | 0.000152946 | 0.0026567 | 0.6134021 | 2.7216495 |
| ENSG00000124257 | NEURL2 | K16782 | -3.7106838 | 4.010570214 | 14.335015 | 0.000152993 | 0.0026567 | 0.3824561 | 2.9719298 |
| ENSG00000134574 | DDB2 | K10140 | -3.7106834 | 4.010570214 | 14.334442 | 0.000153039 | 0.0026567 | 0.412178 | 2.9601874 |
| ENSG00000134802 | SLC43A3 | K08230 | -3.7106829 | 4.010570214 | 14.333869 | 0.000153086 | 0.0026567 | 0.3611111 | 3.1388889 |
| ENSG00000135537 | LACE1 | K18798 | -3.7106825 | 4.010570214 | 14.333296 | 0.000153132 | 0.0026567 | 0.4823285 | 3.0873181 |
| ENSG00000137992 | DBT | K09699 | -3.7106821 | 4.010570214 | 14.332723 | 0.000153179 | 0.0026567 | 0.4439834 | 2.9979253 |
| ENSG00000163945 | UVSSA | - | -3.710682 | 4.010570214 | 14.332507 | 0.000153197 | 0.0026567 | 0.5430183 | 2.909732 |
| ENSG00000148248 | SURF4 | K20369 | -3.7106819 | 4.010570214 | 14.332343 | 0.00015321 | 0.0026567 | 0.3568773 | 3.2453532 |
| ENSG00000164548 | TRA2A | K12897 | -3.7106818 | 4.010570214 | 14.332225 | 0.000153219 | 0.0026567 | 0.5283688 | 2.8475177 |
| ENSG00000145321 | GC | K12258 | -3.7106817 | 4.010570214 | 14.33215 | 0.000153226 | 0.0026567 | 0.5375254 | 2.8924949 |
| ENSG00000170962 | PDGFD | K05450 | -3.7106817 | 4.010570214 | 14.332056 | 0.000153233 | 0.0026567 | 0.5405405 | 3.0567568 |
| ENSG00000181481 | RNF135 | K16272 | -3.7106813 | 4.010570214 | 14.331563 | 0.000153273 | 0.0026567 | 0.4675926 | 2.8240741 |
| ENSG00000186063 | AIDA | - | -3.7106809 | 4.010570214 | 14.331071 | 0.000153313 | 0.0026567 | 0.5196078 | 3.1503268 |
| ENSG00000186815 | TPCN1 | K16896 | -3.7106806 | 4.010570214 | 14.330579 | 0.000153353 | 0.0026567 | 0.4425676 | 3.2488739 |
| ENSG00000189007 | ADAT2 | K15441 | -3.7106802 | 4.010570214 | 14.330087 | 0.000153394 | 0.0026567 | 0.4659686 | 2.7486911 |
| ENSG00000159720 | ATP6V0D1 | K02146 | -3.705096 | 5.038300555 | 12.480013 | 0.000411329 | 0.0047448 | 0.497449 | 3.1658163 |
| ENSG00000147687 | TATDN1 | K03424 | -3.7050954 | 5.038300555 | 12.481078 | 0.000411095 | 0.0047448 | 0.4689441 | 3 |
| ENSG00000146386 | ABRACL | - | -3.7050947 | 5.038300555 | 12.482143 | 0.000410861 | 0.0047448 | 0.4567901 | 2.9876543 |
| ENSG00000111641 | NOP2 | K14835 | -3.6998571 | 5.03531464 | 11.720674 | 0.000618096 | 0.0067819 | 0.4877193 | 2.7953216 |
| ENSG00000166979 | EVA1C | - | -3.6989746 | 5.033657173 | 13.415936 | 0.000249496 | 0.0033303 | 0.5034014 | 2.9229025 |
| ENSG00000177728 | TMEM94 | - | -3.6919387 | 6.140527294 | 10.804698 | 0.001012428 | 0.0091839 | 0.4480234 | 2.9568082 |
| ENSG00000114757 | PEX5L | K13342 | -3.6867014 | 5.023205485 | 12.417978 | 0.00042522 | 0.0048805 | 0.5638978 | 2.841853 |
| ENSG00000154478 | GPR26 | K08411 | -3.6781802 | 3.983146306 | 12.176472 | 0.000483961 | 0.0054671 | 0.3204748 | 3.0296736 |
| ENSG00000120896 | SORBS3 | - | -3.6737385 | 5.199423812 | 11.364711 | 0.000748527 | 0.0074949 | 0.4843517 | 2.923994 |
| ENSG00000172716 | SLFN11 | - | -3.6725343 | 3.979984196 | 14.270227 | 0.00015835 | 0.0026567 | 0.4739179 | 3.0943396 |
| ENSG00000170322 | NFRKB | K11671 | -3.6725341 | 3.979984196 | 14.26997 | 0.000158372 | 0.0026567 | 0.4765861 | 2.6993958 |
| ENSG00000147256 | ARHGAP36 | K20648 | -3.672534 | 3.979984196 | 14.269766 | 0.000158389 | 0.0026567 | 0.4387569 | 2.9945155 |
| ENSG00000141337 | ARSG | K12381 | -3.672534 | 3.979984196 | 14.26975 | 0.00015839 | 0.0026567 | 0.4209524 | 2.8533333 |
| ENSG00000134202 | GSTM3 | K00799 | -3.672534 | 3.979984196 | 14.269734 | 0.000158391 | 0.0026567 | 0.5244444 | 3.3066667 |
| ENSG00000133020 | MYH8 | K10352 | -3.672534 | 3.979984196 | 14.269718 | 0.000158393 | 0.0026567 | 0.6040268 | 3.0144553 |
| ENSG00000132541 | RIDA | - | -3.6725339 | 3.979984196 | 14.269702 | 0.000158394 | 0.0026567 | 0.4594595 | 2.7567568 |
| ENSG00000122729 | ACO1 | K01681 | -3.6725339 | 3.979984196 | 14.269686 | 0.000158395 | 0.0026567 | 0.4454443 | 2.9820023 |
| ENSG00000176476 | SGF29 | K11364 | -3.6725339 | 3.979984196 | 14.269652 | 0.000158398 | 0.0026567 | 0.5085324 | 3.0068259 |
| ENSG00000113356 | POLR3G | K03024 | -3.6725337 | 3.979984196 | 14.269344 | 0.000158424 | 0.0026567 | 0.6738197 | 3.1158798 |
| ENSG00000188483 | IER5L | - | -3.6725335 | 3.979984196 | 14.269076 | 0.000158447 | 0.0026567 | 0.3762376 | 2.5420792 |
| ENSG00000103187 | COTL1 | - | -3.6725334 | 3.979984196 | 14.269002 | 0.000158453 | 0.0026567 | 0.5422535 | 2.971831 |
| ENSG00000100014 | SPECC1L | - | -3.6725331 | 3.979984196 | 14.26858 | 0.000158488 | 0.0026567 | 0.6168308 | 2.7376902 |
| ENSG00000077348 | EXOSC5 | K12590 | -3.6725329 | 3.979984196 | 14.268326 | 0.00015851 | 0.0026567 | 0.4510638 | 2.6510638 |
| ENSG00000020426 | MNAT1 | K10842 | -3.6725326 | 3.979984196 | 14.267857 | 0.000158549 | 0.0026567 | 0.5566343 | 3.1100324 |
| ENSG00000100417 | PMM1 | K17497 | -3.6627275 | 5.402718633 | 12.095742 | 0.000505371 | 0.005681 | 0.5076336 | 3.0458015 |
| ENSG00000187730 | GABRD | K05184 | -3.661941 | 3.971516077 | 14.246136 | 0.00016039 | 0.0026567 | 0.4292035 | 3.039823 |
| ENSG00000183808 | RBM12B | - | -3.6619406 | 3.971516077 | 14.245629 | 0.000160433 | 0.0026567 | 0.5284715 | 3.2237762 |
| ENSG00000182810 | DDX28 | K20096 | -3.6619402 | 3.971516077 | 14.245121 | 0.000160476 | 0.0026567 | 0.387037 | 2.9018519 |
| ENSG00000181631 | P2RY13 | K08388 | -3.6619398 | 3.971516077 | 14.244613 | 0.00016052 | 0.0026567 | 0.3502825 | 3.3502825 |
| ENSG00000174004 | NRROS | - | -3.6619397 | 3.971516077 | 14.244517 | 0.000160528 | 0.0026567 | 0.4364162 | 2.8930636 |
| ENSG00000173369 | C1QB | K03987 | -3.6619395 | 3.971516077 | 14.24424 | 0.000160551 | 0.0026567 | 0.3715415 | 2.6996047 |
| ENSG00000172260 | NEGR1 | K06775 | -3.6619393 | 3.971516077 | 14.243962 | 0.000160575 | 0.0026567 | 0.4717514 | 2.8361582 |
| ENSG00000172086 | KRCC1 | - | -3.6619393 | 3.971516077 | 14.243929 | 0.000160578 | 0.0026567 | 0.6023166 | 3.1621622 |
| ENSG00000171469 | ZNF561 | K09228 | -3.6619393 | 3.971516077 | 14.2439 | 0.00016058 | 0.0026567 | 0.473251 | 2.9773663 |
| ENSG00000171466 | ZNF562 | K09228 | -3.6619393 | 3.971516077 | 14.243872 | 0.000160583 | 0.0026567 | 0.4953052 | 2.9788732 |
| ENSG00000170075 | GPR37L1 | K04244 | -3.6619392 | 3.971516077 | 14.243843 | 0.000160585 | 0.0026567 | 0.3929314 | 2.9126819 |
| ENSG00000165072 | MAMDC2 | - | -3.6619392 | 3.971516077 | 14.243817 | 0.000160588 | 0.0026567 | 0.516035 | 3.058309 |
| ENSG00000169607 | CKAP2L | K16769 | -3.6619392 | 3.971516077 | 14.24374 | 0.000160594 | 0.0026567 | 0.5127517 | 2.9033557 |
| ENSG00000166033 | HTRA1 | K08784 | -3.6619391 | 3.971516077 | 14.243636 | 0.000160603 | 0.0026567 | 0.4229167 | 2.65 |
| ENSG00000162594 | IL23R | K05065 | -3.6619387 | 3.971516077 | 14.243135 | 0.000160646 | 0.0026567 | 0.5484897 | 3.0937997 |
| ENSG00000162402 | USP24 | K11840 | -3.6619384 | 3.971516077 | 14.242747 | 0.000160679 | 0.0026567 | 0.5091603 | 2.978626 |
| ENSG00000160051 | IQCC | - | -3.6619382 | 3.971516077 | 14.242463 | 0.000160703 | 0.0026567 | 0.5384615 | 2.8498169 |
| ENSG00000154127 | UBASH3B | K18993 | -3.661938 | 3.971516077 | 14.242179 | 0.000160727 | 0.0026567 | 0.4684129 | 2.990755 |
| ENSG00000047597 | XK | K19522 | -3.6619379 | 3.971516077 | 14.242055 | 0.000160738 | 0.0026567 | 0.3941441 | 3.3108108 |
| ENSG00000066557 | LRRC40 | - | -3.6619379 | 3.971516077 | 14.242 | 0.000160743 | 0.0026567 | 0.5166113 | 3.0049834 |
| ENSG00000145681 | HAPLN1 | K06848 | -3.6619378 | 3.971516077 | 14.241963 | 0.000160746 | 0.0026567 | 0.4576271 | 3.1158192 |
| ENSG00000068615 | REEP1 | K17338 | -3.6619378 | 3.971516077 | 14.241946 | 0.000160747 | 0.0026567 | 0.4326923 | 2.9807692 |
| ENSG00000147164 | SNX12 | K17918 | -3.6619378 | 3.971516077 | 14.241929 | 0.000160749 | 0.0026567 | 0.494186 | 3.1046512 |
| ENSG00000153208 | MERTK | K05117 | -3.6619378 | 3.971516077 | 14.241895 | 0.000160752 | 0.0026567 | 0.4694695 | 2.9229229 |
| ENSG00000073060 | SCARB1 | K13885 | -3.6619378 | 3.971516077 | 14.241892 | 0.000160752 | 0.0026567 | 0.375 | 2.9855072 |
| ENSG00000043514 | TRIT1 | K00791 | -3.6619378 | 3.971516077 | 14.241868 | 0.000160754 | 0.0026567 | 0.4882227 | 2.9764454 |
| ENSG00000006282 | SPATA20 | - | -3.6619376 | 3.971516077 | 14.241682 | 0.00016077 | 0.0026567 | 0.457606 | 2.9563591 |
| ENSG00000100427 | MLC1 | K20070 | -3.6619375 | 3.971516077 | 14.241466 | 0.000160788 | 0.0026567 | 0.3899204 | 2.9442971 |
| ENSG00000140988 | RPS2 | K02981 | -3.6619373 | 3.971516077 | 14.241274 | 0.000160805 | 0.0026567 | 0.3276451 | 2.7303754 |
| ENSG00000100605 | ITPK1 | K00913 | -3.6619371 | 3.971516077 | 14.24104 | 0.000160825 | 0.0026567 | 0.4758454 | 2.8550725 |
| ENSG00000139832 | RAB20 | K07911 | -3.6619371 | 3.971516077 | 14.240999 | 0.000160828 | 0.0026567 | 0.5 | 2.9188034 |
| ENSG00000101298 | SNPH | - | -3.661937 | 3.971516077 | 14.240869 | 0.000160839 | 0.0026567 | 0.5297398 | 2.5947955 |
| ENSG00000140688 | C16orf58 | - | -3.6619368 | 3.971516077 | 14.240586 | 0.000160863 | 0.0026567 | 0.3974359 | 2.8760684 |
| ENSG00000137878 | GCOM1 | - | -3.6619368 | 3.971516077 | 14.240557 | 0.000160866 | 0.0026567 | 0.6248366 | 2.9071895 |
| ENSG00000105559 | PLEKHA4 | - | -3.6619367 | 3.971516077 | 14.240485 | 0.000160872 | 0.0026567 | 0.4454429 | 2.745828 |
| ENSG00000136842 | TMOD1 | K10370 | -3.6619365 | 3.971516077 | 14.240161 | 0.0001609 | 0.0026567 | 0.5766017 | 2.9554318 |
| ENSG00000110717 | NDUFS8 | K03941 | -3.6619364 | 3.971516077 | 14.240101 | 0.000160905 | 0.0026567 | 0.4809524 | 2.9333333 |
| ENSG00000112182 | BACH2 | K09042 | -3.6619362 | 3.971516077 | 14.239717 | 0.000160938 | 0.0026567 | 0.5600476 | 2.705113 |
| ENSG00000134697 | GNL2 | K14537 | -3.6619361 | 3.971516077 | 14.239647 | 0.000160944 | 0.0026567 | 0.5458276 | 3.0164159 |
| ENSG00000112276 | BVES | K21108 | -3.6619359 | 3.971516077 | 14.239334 | 0.000160971 | 0.0026567 | 0.4694444 | 3.1666667 |
| ENSG00000123739 | PLA2G12A | K01047 | -3.6619357 | 3.971516077 | 14.23913 | 0.000160988 | 0.0026567 | 0.4708995 | 2.8359788 |
| ENSG00000133328 | HRASLS2 | - | -3.6619357 | 3.971516077 | 14.239127 | 0.000160988 | 0.0026567 | 0.4012346 | 2.7530864 |
| ENSG00000129450 | SIGLEC9 | K06740 | -3.6619356 | 3.971516077 | 14.239017 | 0.000160998 | 0.0026567 | 0.4091858 | 2.8914405 |
| ENSG00000115194 | SLC30A3 | K14690 | -3.6619356 | 3.971516077 | 14.23895 | 0.000161003 | 0.0026567 | 0.3814433 | 2.8427835 |
| ENSG00000125895 | TMEM74B | - | -3.6619354 | 3.971516077 | 14.238661 | 0.000161028 | 0.0026567 | 0.4375 | 2.7070313 |
| ENSG00000132535 | DLG4 | K11828 | -3.6619353 | 3.971516077 | 14.238606 | 0.000161033 | 0.0026567 | 0.5189048 | 2.9230769 |
| ENSG00000164850 | GPER1 | K04246 | -3.6566625 | 6.111255791 | 12.252967 | 0.000464519 | 0.0052842 | 0.3235294 | 3.0539216 |
| ENSG00000167470 | MIDN | - | -3.6430375 | 5.174373217 | 12.025286 | 0.000524837 | 0.0058312 | 0.4188034 | 2.4957265 |
| ENSG00000130226 | DPP6 | - | -3.6407539 | 5.172145041 | 10.426508 | 0.001242194 | 0.0096891 | 0.5202312 | 3.0300578 |
| ENSG00000171714 | ANO5 | K19480 | -3.6407538 | 5.172145041 | 10.426845 | 0.001241967 | 0.0096891 | 0.4884995 | 3.3921139 |
| ENSG00000162878 | PKDCC | K17548 | -3.6400561 | 5.171793194 | 11.248332 | 0.000796946 | 0.0075208 | 0.4036511 | 2.8559838 |
| ENSG00000118473 | SGIP1 | K20065 | -3.6343168 | 5.568107357 | 13.052669 | 0.000302853 | 0.0039686 | 0.4796275 | 2.7834692 |
| ENSG00000004660 | CAMKK1 | K00908 | -3.633907 | 5.674105097 | 11.669235 | 0.000635421 | 0.0069322 | 0.4718045 | 2.8984962 |
| ENSG00000112186 | CAP2 | K17261 | -3.6290365 | 5.563813936 | 12.10002 | 0.000504213 | 0.0056735 | 0.5031447 | 2.8972746 |
| ENSG00000123815 | ADCK4 | K08869 | -3.6286552 | 5.66943697 | 12.432218 | 0.00042199 | 0.0048531 | 0.4246324 | 2.8694853 |
| ENSG00000134470 | IL15RA | K05074 | -3.6286551 | 5.66943697 | 12.432337 | 0.000421964 | 0.0048531 | 0.4362606 | 2.5467422 |
| ENSG00000165914 | TTC7B | - | -3.6171607 | 5.659567656 | 11.356436 | 0.00075187 | 0.0074949 | 0.4721234 | 2.9418743 |
| ENSG00000095970 | TREM2 | K14378 | -3.6098217 | 5.566327125 | 11.009765 | 0.000906331 | 0.0083532 | 0.4347826 | 2.9217391 |
| ENSG00000106144 | CASP2 | K02186 | -3.5940837 | 5.863705443 | 11.879297 | 0.000567614 | 0.0062821 | 0.460177 | 2.9314159 |
| ENSG00000147874 | HAUS6 | K16589 | -3.5879053 | 6.039085535 | 12.040348 | 0.000520613 | 0.0057909 | 0.5465969 | 3.008377 |
| ENSG00000172031 | EPHX4 | - | -3.586896 | 5.828687384 | 12.651035 | 0.000375358 | 0.0047009 | 0.4005525 | 3.378453 |
| ENSG00000135426 | TESPA1 | - | -3.5775643 | 4.933734398 | 11.262166 | 0.000791028 | 0.0074949 | 0.5681382 | 2.9443378 |
| ENSG00000064545 | TMEM161A | - | -3.5732606 | 6.026966227 | 11.754849 | 0.000606849 | 0.0066649 | 0.3528184 | 3.1565762 |
| ENSG00000176209 | SMIM19 | - | -3.5698992 | 4.92776856 | 12.045576 | 0.000519155 | 0.0057905 | 0.5607477 | 3.1775701 |
| ENSG00000153214 | TMEM87B | - | -3.569899 | 4.92776856 | 12.045934 | 0.000519055 | 0.0057905 | 0.4288288 | 3.1945946 |
| ENSG00000146909 | NOM1 | K17583 | -3.5698987 | 4.92776856 | 12.046292 | 0.000518955 | 0.0057905 | 0.5127907 | 2.8604651 |
| ENSG00000010539 | ZNF200 | K09228 | -3.5664333 | 4.924748703 | 11.230018 | 0.000804848 | 0.0075581 | 0.5392405 | 2.9898734 |
| ENSG00000120910 | PPP3CC | K04348 | -3.5664332 | 4.924748703 | 11.230192 | 0.000804773 | 0.0075581 | 0.4894434 | 3.0690979 |
| ENSG00000061938 | TNK2 | K08886 | -3.5664331 | 4.924748703 | 11.230317 | 0.000804719 | 0.0075581 | 0.4235727 | 2.8637201 |
| ENSG00000166292 | TMEM100 | - | -3.566433 | 4.924748703 | 11.230475 | 0.000804651 | 0.0075581 | 0.3507463 | 2.8283582 |
| ENSG00000119125 | GDA | K01487 | -3.5664329 | 4.924748703 | 11.230615 | 0.00080459 | 0.0075581 | 0.5053079 | 3.0254777 |
| ENSG00000169660 | HEXDC | K14459 | -3.5664328 | 4.924748703 | 11.230894 | 0.000804469 | 0.0075581 | 0.3623932 | 2.8017094 |
| ENSG00000167778 | SPRYD3 | - | -3.5626053 | 6.096110151 | 12.878062 | 0.000332456 | 0.0043268 | 0.4926931 | 2.9311065 |
| ENSG00000133687 | TMTC1 | - | -3.553898 | 5.519560154 | 10.59124 | 0.001136247 | 0.0095882 | 0.3993644 | 3.092161 |
| ENSG00000174521 | TTC9B | - | -3.5496915 | 4.911488907 | 11.982186 | 0.000537115 | 0.0059561 | 0.4351464 | 2.7154812 |
| ENSG00000167881 | SRP68 | K03107 | -3.5496914 | 4.911488907 | 11.982344 | 0.00053707 | 0.0059561 | 0.5023923 | 2.9744817 |
| ENSG00000080644 | CHRNA3 | K04805 | -3.5423155 | 5.491931635 | 10.595559 | 0.001133596 | 0.0095882 | 0.4475248 | 3.2039604 |
| ENSG00000088320 | REM1 | K07847 | -3.5379657 | 5.08857025 | 11.677152 | 0.000632723 | 0.0069093 | 0.4899329 | 2.7684564 |
| ENSG00000168575 | SLC20A2 | K14640 | -3.5379656 | 5.08857025 | 11.677345 | 0.000632658 | 0.0069093 | 0.3895706 | 2.9003067 |
| ENSG00000135482 | ZC3H10 | - | -3.5379654 | 5.08857025 | 11.677833 | 0.000632492 | 0.0069093 | 0.4285714 | 2.5852535 |
| ENSG00000178177 | LCORL | - | -3.5337483 | 5.297143242 | 12.559124 | 0.000394276 | 0.0047009 | 0.5548173 | 2.7790698 |
| ENSG00000124222 | STX16 | K08489 | -3.533748 | 5.297143242 | 12.561368 | 0.000393803 | 0.0047009 | 0.5415385 | 2.9569231 |
| ENSG00000111859 | NEDD9 | K16832 | -3.5318912 | 6.22415012 | 12.065853 | 0.000513538 | 0.0057615 | 0.5119904 | 2.882494 |
| ENSG00000134817 | APLNR | K04174 | -3.5274443 | 5.291981518 | 11.62676 | 0.000650097 | 0.007052 | 0.4131579 | 3.0868421 |
| ENSG00000153066 | TXNDC11 | - | -3.5274443 | 5.291981518 | 11.627134 | 0.000649966 | 0.007052 | 0.4781726 | 3.0233503 |
| ENSG00000123983 | ACSL3 | K01897 | -3.525984 | 5.585336414 | 12.040028 | 0.000520702 | 0.0057909 | 0.4541667 | 3.0055556 |
| ENSG00000148468 | FAM171A1 | - | -3.5259836 | 5.585336414 | 12.04069 | 0.000520517 | 0.0057909 | 0.505618 | 2.8067416 |
| ENSG00000149485 | FADS1 | K10224 | -3.5242158 | 5.077479032 | 11.627881 | 0.000649705 | 0.007052 | 0.3453094 | 3.3333333 |
| ENSG00000176788 | BASP1 | K17272 | -3.5239358 | 6.824225492 | 13.574044 | 0.000229335 | 0.0030827 | 0.6079295 | 2.2114537 |
| ENSG00000151229 | SLC2A13 | K08150 | -3.5087566 | 5.066367348 | 10.86846 | 0.000978159 | 0.0089438 | 0.4243827 | 2.8796296 |
| ENSG00000140577 | CRTC3 | K16334 | -3.5053087 | 6.310309629 | 12.663346 | 0.000372894 | 0.0047009 | 0.5024233 | 2.7189015 |
| ENSG00000135372 | NAT10 | K14521 | -3.5051844 | 6.097071921 | 11.253252 | 0.000794836 | 0.0075071 | 0.4712195 | 3.0390244 |
| ENSG00000162236 | STX5 | K08490 | -3.4998881 | 5.681200726 | 10.619128 | 0.001119238 | 0.0095882 | 0.4985915 | 2.9183099 |
| ENSG00000100884 | CPNE6 | - | -3.4987196 | 5.679526637 | 10.401522 | 0.001259115 | 0.0097784 | 0.496732 | 2.8594771 |
| ENSG00000179933 | C14orf119 | - | -3.4957136 | 5.054556123 | 11.549555 | 0.000677655 | 0.0073233 | 0.5 | 3.15 |
| ENSG00000084207 | GSTP1 | K00799 | -3.4719838 | 4.846405461 | 10.663472 | 0.001092721 | 0.0095882 | 0.4666667 | 3.0238095 |
| ENSG00000149179 | C11orf49 | - | -3.4680498 | 5.242603778 | 10.404975 | 0.001256762 | 0.0097761 | 0.4896142 | 3.0445104 |
| ENSG00000151778 | SERP2 | - | -3.4661071 | 4.841717031 | 10.644899 | 0.001103749 | 0.0095882 | 0.3452381 | 2.8690476 |
| ENSG00000148824 | MTG1 | K19828 | -3.4661068 | 4.841717031 | 10.645275 | 0.001103525 | 0.0095882 | 0.3832335 | 2.9401198 |
| ENSG00000144118 | RALB | K07835 | -3.4661066 | 4.841717031 | 10.645687 | 0.001103279 | 0.0095882 | 0.5679612 | 2.9902913 |
| ENSG00000139324 | TMTC3 | - | -3.4661062 | 4.841717031 | 10.646251 | 0.001102943 | 0.0095882 | 0.4321663 | 3.1597374 |
| ENSG00000120742 | SERP1 | - | -3.4661058 | 4.841717031 | 10.646764 | 0.001102637 | 0.0095882 | 0.3974359 | 2.9102564 |
| ENSG00000110851 | PRDM4 | K12463 | -3.4661057 | 4.841717031 | 10.646901 | 0.001102555 | 0.0095882 | 0.5018727 | 2.7503121 |
| ENSG00000157911 | PEX10 | K13346 | -3.4563716 | 6.008409858 | 10.972995 | 0.000924492 | 0.008487 | 0.3439306 | 3.0462428 |
| ENSG00000007202 | KIAA0100 | - | -3.438334 | 5.707379762 | 10.920365 | 0.000951129 | 0.0087106 | 0.4796421 | 3.0474273 |
| ENSG00000170775 | GPR37 | K04243 | -3.4374112 | 4.818897759 | 10.559369 | 0.001156007 | 0.0095882 | 0.3898858 | 2.8548124 |
| ENSG00000162757 | C1orf74 | - | -3.4301692 | 4.813480765 | 11.312429 | 0.0007699 | 0.0074949 | 0.4237918 | 2.9442379 |
| ENSG00000103365 | GGA2 | K12404 | -3.4265447 | 4.998767723 | 10.534638 | 0.001171578 | 0.0095882 | 0.4486134 | 2.8743883 |
| ENSG00000186501 | TMEM222 | K20726 | -3.4152338 | 4.801333249 | 10.493744 | 0.001197794 | 0.0095882 | 0.3798077 | 3.0769231 |
| ENSG00000137960 | GIPC2 | K20056 | -3.4152332 | 4.801333249 | 10.494601 | 0.001197238 | 0.0095882 | 0.4666667 | 2.8539683 |
| ENSG00000165775 | FUNDC2 | - | -3.4141561 | 5.687964264 | 10.821627 | 0.001003213 | 0.0091148 | 0.4126984 | 2.8994709 |
| ENSG00000132434 | LANCL2 | - | -3.4141559 | 5.687964264 | 10.822179 | 0.001002914 | 0.0091148 | 0.4644444 | 3.0133333 |
| ENSG00000168288 | MMADHC | - | -3.3949236 | 6.006829337 | 10.485085 | 0.001203421 | 0.0095882 | 0.5 | 2.8272727 |
| ENSG00000070814 | TCOF1 | K14562 | -3.386506 | 4.967045376 | 10.400802 | 0.001259606 | 0.0097784 | 0.5389785 | 2.313172 |
| ENSG00000162711 | NLRP3 | K12800 | -3.3844505 | 4.965534192 | 11.164482 | 0.000833782 | 0.0077662 | 0.4874517 | 3.0453668 |
| ENSG00000164099 | PRSS12 | K09624 | -3.3832882 | 3.753172091 | 12.51994 | 0.000402632 | 0.0047009 | 0.4171429 | 2.8457143 |
| ENSG00000163558 | PRKCI | K06069 | -3.3832878 | 3.753172091 | 12.519601 | 0.000402705 | 0.0047009 | 0.5201342 | 3.0419463 |
| ENSG00000162728 | KCNJ9 | K05002 | -3.3832875 | 3.753172091 | 12.519263 | 0.000402778 | 0.0047009 | 0.4885496 | 2.9898219 |
| ENSG00000158445 | KCNB1 | K04885 | -3.3832874 | 3.753172091 | 12.519181 | 0.000402795 | 0.0047009 | 0.4918415 | 2.9324009 |
| ENSG00000165061 | ZMAT4 | - | -3.3832874 | 3.753172091 | 12.519172 | 0.000402797 | 0.0047009 | 0.4541485 | 2.860262 |
| ENSG00000157020 | SEC13 | K14004 | -3.3832872 | 3.753172091 | 12.518949 | 0.000402846 | 0.0047009 | 0.4918478 | 2.8451087 |
| ENSG00000165678 | GHITM | - | -3.3832871 | 3.753172091 | 12.518891 | 0.000402858 | 0.0047009 | 0.3043478 | 2.8927536 |
| ENSG00000165794 | SLC39A2 | K14709 | -3.3832869 | 3.753172091 | 12.51861 | 0.000402919 | 0.0047009 | 0.3042071 | 2.8414239 |
| ENSG00000155511 | GRIA1 | K05197 | -3.3832868 | 3.753172091 | 12.518589 | 0.000402923 | 0.0047009 | 0.4716157 | 3.0272926 |
| ENSG00000166189 | HPS6 | K20192 | -3.3832866 | 3.753172091 | 12.518329 | 0.000402979 | 0.0047009 | 0.3664516 | 2.7935484 |
| ENSG00000150995 | ITPR1 | K04958 | -3.3832865 | 3.753172091 | 12.518229 | 0.000403001 | 0.0047009 | 0.5166788 | 3.0326323 |
| ENSG00000188917 | TRMT2B | K15331 | -3.3832864 | 3.753172091 | 12.518134 | 0.000403021 | 0.0047009 | 0.4107143 | 3.0515873 |
| ENSG00000167861 | HID1 | - | -3.3832863 | 3.753172091 | 12.518049 | 0.00040304 | 0.0047009 | 0.4720812 | 3.0824873 |
| ENSG00000150637 | CD226 | K06567 | -3.3832861 | 3.753172091 | 12.517869 | 0.000403078 | 0.0047009 | 0.5029762 | 3.1220238 |
| ENSG00000187017 | ESPN | - | -3.3832861 | 3.753172091 | 12.517792 | 0.000403095 | 0.0047009 | 0.442623 | 2.6733021 |
| ENSG00000169180 | XPO6 | - | -3.383286 | 3.753172091 | 12.517768 | 0.0004031 | 0.0047009 | 0.4817778 | 3.1733333 |
| ENSG00000185252 | ZNF74 | K09228 | -3.383286 | 3.753172091 | 12.517752 | 0.000403104 | 0.0047009 | 0.4301242 | 2.878882 |
| ENSG00000196083 | IL1RAP | K04723 | -3.3832858 | 3.753172091 | 12.517561 | 0.000403145 | 0.0047009 | 0.4949054 | 3.1120815 |
| ENSG00000149806 | FAU | K02983 | -3.3832858 | 3.753172091 | 12.517509 | 0.000403156 | 0.0047009 | 0.3759399 | 2.7293233 |
| ENSG00000169435 | RASSF6 | K09854 | -3.3832858 | 3.753172091 | 12.517487 | 0.000403161 | 0.0047009 | 0.5474255 | 3.2303523 |
| ENSG00000183878 | UTY | K11447 | -3.3832857 | 3.753172091 | 12.517467 | 0.000403165 | 0.0047009 | 0.5 | 2.8545706 |
| ENSG00000146192 | FGD2 | K05721 | -3.3832855 | 3.753172091 | 12.517225 | 0.000403217 | 0.0047009 | 0.5022901 | 3.0305344 |
| ENSG00000170091 | HMP19 | - | -3.3832855 | 3.753172091 | 12.517207 | 0.000403221 | 0.0047009 | 0.4152047 | 3.0994152 |
| ENSG00000149428 | HYOU1 | K09486 | -3.3832855 | 3.753172091 | 12.517202 | 0.000403222 | 0.0047009 | 0.5365365 | 2.8998999 |
| ENSG00000183508 | FAM46C | - | -3.3832855 | 3.753172091 | 12.517181 | 0.000403227 | 0.0047009 | 0.5217391 | 3.140665 |
| ENSG00000146411 | SLC2A12 | K08149 | -3.3832853 | 3.753172091 | 12.516963 | 0.000403274 | 0.0047009 | 0.3922204 | 2.8849271 |
| ENSG00000171033 | PKIA | K15985 | -3.3832852 | 3.753172091 | 12.516927 | 0.000403282 | 0.0047009 | 0.6842105 | 2.3289474 |
| ENSG00000182472 | CAPN12 | K04740 | -3.3832852 | 3.753172091 | 12.516896 | 0.000403288 | 0.0047009 | 0.449235 | 2.9972184 |
| ENSG00000145284 | SCD5 | K00507 | -3.383285 | 3.753172091 | 12.516736 | 0.000403323 | 0.0047009 | 0.3636364 | 3.1939394 |
| ENSG00000172995 | ARPP21 | - | -3.3832849 | 3.753172091 | 12.516647 | 0.000403342 | 0.0047009 | 0.5559656 | 2.6728167 |
| ENSG00000182103 | FAM181B | - | -3.3832849 | 3.753172091 | 12.51661 | 0.00040335 | 0.0047009 | 0.3615023 | 2.4366197 |
| ENSG00000142002 | DPP9 | K08656 | -3.3832847 | 3.753172091 | 12.516422 | 0.000403391 | 0.0047009 | 0.4831839 | 3.1334081 |
| ENSG00000173451 | THAP2 | - | -3.3832847 | 3.753172091 | 12.516372 | 0.000403402 | 0.0047009 | 0.4122807 | 3.0745614 |
| ENSG00000141076 | UTP4 | K14548 | -3.3832846 | 3.753172091 | 12.516349 | 0.000403406 | 0.0047009 | 0.4328571 | 2.9771429 |
| ENSG00000139154 | AEBP2 | K17452 | -3.3832846 | 3.753172091 | 12.516275 | 0.000403422 | 0.0047009 | 0.5647969 | 2.4197292 |
| ENSG00000173486 | FKBP2 | K09569 | -3.3832844 | 3.753172091 | 12.51614 | 0.000403452 | 0.0047009 | 0.3943662 | 2.9084507 |
| ENSG00000174640 | SLCO2A1 | K14345 | -3.3832844 | 3.753172091 | 12.516138 | 0.000403452 | 0.0047009 | 0.3452566 | 2.9533437 |
| ENSG00000176658 | MYO1D | K10356 | -3.3832844 | 3.753172091 | 12.516137 | 0.000403452 | 0.0047009 | 0.4662028 | 3.1858847 |
| ENSG00000132424 | PNISR | K13170 | -3.3832843 | 3.753172091 | 12.51602 | 0.000403477 | 0.0047009 | 0.6049689 | 2.8173913 |
| ENSG00000132932 | ATP8A2 | K14802 | -3.3832842 | 3.753172091 | 12.515903 | 0.000403503 | 0.0047009 | 0.4520202 | 3.0513468 |
| ENSG00000127152 | BCL11B | - | -3.3832841 | 3.753172091 | 12.515813 | 0.000403522 | 0.0047009 | 0.450783 | 2.6308725 |
| ENSG00000135119 | RNFT2 | - | -3.3832841 | 3.753172091 | 12.515785 | 0.000403528 | 0.0047009 | 0.3896396 | 2.9594595 |
| ENSG00000137700 | SLC37A4 | K08171 | -3.3832841 | 3.753172091 | 12.515772 | 0.000403531 | 0.0047009 | 0.3547672 | 3.0022173 |
| ENSG00000136531 | SCN2A | K04834 | -3.383284 | 3.753172091 | 12.515668 | 0.000403553 | 0.0047009 | 0.478803 | 3.1436409 |
| ENSG00000126767 | ELK1 | K04375 | -3.3832838 | 3.753172091 | 12.515497 | 0.00040359 | 0.0047009 | 0.4205607 | 2.6588785 |
| ENSG00000126070 | AGO3 | K11593 | -3.3832835 | 3.753172091 | 12.515181 | 0.000403659 | 0.0047009 | 0.4267442 | 3.044186 |
| ENSG00000124702 | KLHDC3 | - | -3.383283 | 3.753172091 | 12.514642 | 0.000403775 | 0.0047009 | 0.4190231 | 3.0282776 |
| ENSG00000124217 | MOCS3 | K11996 | -3.3832826 | 3.753172091 | 12.51428 | 0.000403853 | 0.0047009 | 0.4195652 | 2.7652174 |
| ENSG00000121897 | LIAS | K03644 | -3.3832821 | 3.753172091 | 12.513737 | 0.000403971 | 0.0047009 | 0.4784946 | 2.9650538 |
| ENSG00000117280 | RAB29 | K07916 | -3.3832817 | 3.753172091 | 12.513312 | 0.000404063 | 0.0047009 | 0.546798 | 2.9802956 |
| ENSG00000120662 | MTRF1 | K02835 | -3.3832816 | 3.753172091 | 12.513194 | 0.000404088 | 0.0047009 | 0.5213483 | 3.1460674 |
| ENSG00000119541 | VPS4B | K12196 | -3.3832815 | 3.753172091 | 12.513188 | 0.00040409 | 0.0047009 | 0.5067568 | 2.9054054 |
| ENSG00000119535 | CSF3R | K05061 | -3.3832815 | 3.753172091 | 12.513182 | 0.000404091 | 0.0047009 | 0.4310545 | 2.8991889 |
| ENSG00000119147 | C2orf40 | - | -3.3832815 | 3.753172091 | 12.513176 | 0.000404092 | 0.0047009 | 0.4932432 | 3.2162162 |
| ENSG00000110696 | C11orf58 | - | -3.3832813 | 3.753172091 | 12.512919 | 0.000404148 | 0.0047009 | 0.7540984 | 2.6010929 |
| ENSG00000109771 | LRP2BP | - | -3.3832809 | 3.753172091 | 12.512527 | 0.000404233 | 0.0047009 | 0.4985591 | 3.1469741 |
| ENSG00000108950 | FAM20A | - | -3.3832803 | 3.753172091 | 12.511954 | 0.000404356 | 0.0047009 | 0.4436229 | 3.0573013 |
| ENSG00000105650 | PDE4C | K13293 | -3.3832798 | 3.753172091 | 12.511404 | 0.000404476 | 0.0047009 | 0.5547753 | 2.8735955 |
| ENSG00000105143 | SLC1A6 | K05617 | -3.3832793 | 3.753172091 | 12.510854 | 0.000404595 | 0.0047009 | 0.3723404 | 2.9148936 |
| ENSG00000100425 | BRD1 | K11349 | -3.3832787 | 3.753172091 | 12.51032 | 0.00040471 | 0.0047009 | 0.4751892 | 2.8637511 |
| ENSG00000103550 | KNOP1 | - | -3.3832787 | 3.753172091 | 12.510303 | 0.000404714 | 0.0047009 | 0.4956332 | 2.9126638 |
| ENSG00000072201 | LNX1 | K10692 | -3.3832785 | 3.753172091 | 12.510041 | 0.000404771 | 0.0047009 | 0.4739011 | 2.8282967 |
| ENSG00000100362 | PVALB | - | -3.3832784 | 3.753172091 | 12.510024 | 0.000404774 | 0.0047009 | 0.5363636 | 2.8454545 |
| ENSG00000070761 | CFAP20 | - | -3.3832782 | 3.753172091 | 12.50979 | 0.000404825 | 0.0047009 | 0.4715026 | 3.3367876 |
| ENSG00000099139 | PCSK5 | K08654 | -3.3832781 | 3.753172091 | 12.509729 | 0.000404838 | 0.0047009 | 0.5193548 | 2.6903226 |
| ENSG00000074935 | TUBE1 | K10391 | -3.383278 | 3.753172091 | 12.509583 | 0.00040487 | 0.0047009 | 0.4736842 | 2.9347368 |
| ENSG00000070614 | NDST1 | K02576 | -3.383278 | 3.753172091 | 12.509539 | 0.00040488 | 0.0047009 | 0.4387755 | 3.2278912 |
| ENSG00000073464 | CLCN4 | K05012 | -3.3832779 | 3.753172091 | 12.509521 | 0.000404883 | 0.0047009 | 0.4026316 | 3.0776316 |
| ENSG00000083457 | ITGAE | K06524 | -3.3832779 | 3.753172091 | 12.509428 | 0.000404904 | 0.0047009 | 0.4885496 | 2.8736217 |
| ENSG00000065802 | ASB1 | K10323 | -3.3832778 | 3.753172091 | 12.509335 | 0.000404924 | 0.0047009 | 0.3880597 | 2.8656716 |
| ENSG00000069424 | KCNAB2 | K04883 | -3.3832777 | 3.753172091 | 12.509287 | 0.000404934 | 0.0047009 | 0.4626506 | 2.9228916 |
| ENSG00000089009 | RPL6 | K02934 | -3.3832777 | 3.753172091 | 12.509273 | 0.000404937 | 0.0047009 | 0.34375 | 3.1284722 |
| ENSG00000089250 | NOS1 | K13240 | -3.3832775 | 3.753172091 | 12.509118 | 0.000404971 | 0.0047009 | 0.4775204 | 2.9822888 |
| ENSG00000092607 | TBX15 | K10182 | -3.3832775 | 3.753172091 | 12.509108 | 0.000404973 | 0.0047009 | 0.5033223 | 2.7408638 |
| ENSG00000000003 | TSPAN6 | K17295 | -3.3832775 | 3.753172091 | 12.5091 | 0.000404975 | 0.0047009 | 0.4081633 | 3.1346939 |
| ENSG00000005189 | LOC81691 | K14570 | -3.3832773 | 3.753172091 | 12.50888 | 0.000405022 | 0.0047009 | 0.4418605 | 3.0155039 |
| ENSG00000054392 | HHAT | - | -3.3832771 | 3.753172091 | 12.50866 | 0.00040507 | 0.0047009 | 0.3684211 | 3.3076923 |
| ENSG00000151135 | TMEM263 | - | -3.3789869 | 4.961218588 | 11.147338 | 0.000841524 | 0.0078319 | 0.4137931 | 2.387931 |
| ENSG00000172354 | GNB2 | K04537 | -3.3787017 | 5.945721831 | 10.630648 | 0.001112287 | 0.0095882 | 0.4852941 | 2.7294118 |
| ENSG00000160271 | RALGDS | K08732 | -3.3782154 | 5.868074426 | 10.864536 | 0.000980233 | 0.0089557 | 0.5142232 | 2.8555799 |
| ENSG00000065911 | MTHFD2 | K13403 | -3.375383 | 4.958262277 | 10.364704 | 0.001284475 | 0.0099512 | 0.4 | 2.7657143 |
| ENSG00000162636 | FAM102B | - | -3.3680472 | 5.351226213 | 10.857941 | 0.00098373 | 0.0089805 | 0.5333333 | 2.7027778 |
| ENSG00000173273 | TNKS | K10799 | -3.3631598 | 5.871257524 | 10.895904 | 0.000963772 | 0.0088193 | 0.4400904 | 2.6616428 |
| ENSG00000176624 | MEX3C | K15686 | -3.3587924 | 4.945308116 | 11.081777 | 0.000871802 | 0.0080679 | 0.4810319 | 2.5584219 |
| ENSG00000137965 | IFI44 | - | -3.3587923 | 4.945308116 | 11.081899 | 0.000871745 | 0.0080679 | 0.4977477 | 3.0608108 |
| ENSG00000134020 | PEBP4 | - | -3.3587922 | 4.945308116 | 11.082298 | 0.000871557 | 0.0080679 | 0.4669604 | 3.0352423 |
| ENSG00000131115 | ZNF227 | K09228 | -3.3587921 | 4.945308116 | 11.082399 | 0.00087151 | 0.0080679 | 0.4843554 | 2.9962453 |
| ENSG00000115419 | GLS | K01425 | -3.358792 | 4.945308116 | 11.082619 | 0.000871406 | 0.0080679 | 0.4289985 | 2.8654709 |
| ENSG00000171914 | TLN2 | K06271 | -3.3534916 | 6.624485591 | 12.331235 | 0.000445441 | 0.0050871 | 0.5043273 | 2.6117231 |
| ENSG00000168792 | ABHD15 | K13707 | -3.347173 | 5.633898278 | 11.320082 | 0.000766733 | 0.0074949 | 0.3846154 | 2.9423077 |
| ENSG00000010165 | METTL13 | - | -3.3471729 | 5.633898278 | 11.320405 | 0.0007666 | 0.0074949 | 0.4577969 | 3.0114449 |
| ENSG00000110497 | AMBRA1 | K17985 | -3.3431407 | 5.331406909 | 11.642842 | 0.000644501 | 0.0070112 | 0.5138675 | 2.7218798 |
| ENSG00000088876 | ZNF343 | K09228 | -3.3305464 | 5.620702449 | 11.25912 | 0.000792328 | 0.0074949 | 0.478125 | 2.940625 |
| ENSG00000188566 | NDOR1 | - | -3.3256274 | 5.616561504 | 10.421123 | 0.001245821 | 0.0097042 | 0.4240924 | 3.0165017 |
| ENSG00000112406 | HECA | - | -3.3256274 | 5.616561504 | 10.421219 | 0.001245756 | 0.0097042 | 0.3867403 | 2.7053407 |
| ENSG00000174791 | RIN1 | K17638 | -3.3235081 | 5.127275723 | 10.645967 | 0.001103112 | 0.0095882 | 0.4214559 | 2.706258 |
| ENSG00000160209 | LOC105372824 | - | -3.3061541 | 6.433204123 | 12.232689 | 0.000469595 | 0.0053205 | 0.4839744 | 2.9583333 |
| ENSG00000156453 | PCDH1 | K16498 | -3.2996673 | 5.108542655 | 10.562168 | 0.001154258 | 0.0095882 | 0.5464834 | 2.7178658 |
| ENSG00000038274 | MAT2B | K00789 | -3.2996673 | 5.108542655 | 10.56233 | 0.001154156 | 0.0095882 | 0.4461078 | 2.9820359 |
| ENSG00000168389 | MFSD2A | - | -3.2996671 | 5.108542655 | 10.563158 | 0.001153639 | 0.0095882 | 0.3959484 | 3.0976059 |
| ENSG00000143256 | PFDN2 | K09549 | -3.2939539 | 5.104121009 | 11.394469 | 0.000736631 | 0.0074949 | 0.525974 | 2.5974026 |
| ENSG00000172247 | C1QTNF4 | - | -3.2865089 | 5.098235645 | 10.508129 | 0.001188505 | 0.0095882 | 0.3860182 | 2.787234 |
| ENSG00000164076 | CAMKV | K08812 | -3.2812991 | 5.094157609 | 10.486987 | 0.001202182 | 0.0095882 | 0.5329341 | 2.7265469 |
| ENSG00000078246 | TULP3 | - | -3.270738 | 4.688068799 | 10.82377 | 0.001002052 | 0.0091148 | 0.493014 | 2.9221557 |
| ENSG00000118482 | PHF3 | - | -3.270738 | 4.688068799 | 10.823782 | 0.001002046 | 0.0091148 | 0.5801864 | 2.8430603 |
| ENSG00000156345 | CDK20 | K08817 | -3.2707379 | 4.688068799 | 10.82403 | 0.001001912 | 0.0091148 | 0.3901734 | 3.1040462 |
| ENSG00000175764 | TTLL11 | K16604 | -3.2639872 | 4.682847257 | 10.801286 | 0.001014296 | 0.0091936 | 0.42 | 2.8475 |
| ENSG00000151062 | CACNA2D4 | K04861 | -3.248116 | 5.890216476 | 10.812868 | 0.00100797 | 0.0091507 | 0.4870017 | 3.0311958 |
| ENSG00000124614 | RPS10 | K02947 | -3.2459592 | 6.101521615 | 11.302588 | 0.000773991 | 0.0074949 | 0.3953488 | 3.1569767 |
| ENSG00000160539 | PLPP7 | - | -3.2392822 | 4.849682853 | 10.401515 | 0.00125912 | 0.0097784 | 0.3247232 | 2.8265683 |
| ENSG00000137269 | LRRC1 | - | -3.2389785 | 4.66357291 | 10.724047 | 0.001057521 | 0.0095402 | 0.5515267 | 2.9618321 |
| ENSG00000165209 | STRBP | K13200 | -3.2345219 | 5.351790861 | 10.637113 | 0.001108406 | 0.0095882 | 0.4732143 | 2.796131 |
| ENSG00000103740 | ACSBG1 | K15013 | -3.172173 | 6.358042328 | 10.695194 | 0.001074142 | 0.0095882 | 0.4820442 | 2.9834254 |
| ENSG00000145782 | ATG12 | K08336 | -3.1095723 | 6.309285793 | 10.366521 | 0.001283212 | 0.0099481 | 0.5 | 2.8285714 |
| ENSG00000180801 | ARSJ | K12375 | -3.0925628 | 6.197445174 | 10.518694 | 0.00118173 | 0.0095882 | 0.4524207 | 3.0233723 |
| ENSG00000163430 | FSTL1 | - | -3.0848814 | 3.518550409 | 11.414264 | 0.000728824 | 0.0074832 | 0.5584416 | 2.8928571 |
| ENSG00000159840 | ZYX | K06273 | -3.084881 | 3.518550409 | 11.413846 | 0.000728988 | 0.0074832 | 0.4248252 | 2.7255245 |
| ENSG00000164713 | BRI3 | - | -3.084881 | 3.518550409 | 11.413806 | 0.000729003 | 0.0074832 | 0.264 | 3.008 |
| ENSG00000158710 | TAGLN2 | K20526 | -3.0848806 | 3.518550409 | 11.413428 | 0.000729152 | 0.0074832 | 0.4681818 | 2.8681818 |
| ENSG00000164736 | SOX17 | K04495 | -3.0848806 | 3.518550409 | 11.413348 | 0.000729183 | 0.0074832 | 0.4057971 | 2.6835749 |
| ENSG00000154330 | PGM5 | K15636 | -3.0848803 | 3.518550409 | 11.41301 | 0.000729316 | 0.0074832 | 0.4338624 | 2.8924162 |
| ENSG00000165806 | CASP7 | K04397 | -3.0848801 | 3.518550409 | 11.412889 | 0.000729363 | 0.0074832 | 0.4458763 | 2.9664948 |
| ENSG00000146112 | PPP1R18 | K17559 | -3.0848799 | 3.518550409 | 11.412592 | 0.00072948 | 0.0074832 | 0.5448613 | 2.7553018 |
| ENSG00000165905 | GYLTL1B | K09668 | -3.0848797 | 3.518550409 | 11.412432 | 0.000729543 | 0.0074832 | 0.3925104 | 3.148405 |
| ENSG00000145649 | GZMA | K01352 | -3.0848795 | 3.518550409 | 11.412173 | 0.000729644 | 0.0074832 | 0.4007634 | 2.8664122 |
| ENSG00000169908 | TM4SF1 | - | -3.0848795 | 3.518550409 | 11.412128 | 0.000729662 | 0.0074832 | 0.3405172 | 2.8491379 |
| ENSG00000167601 | AXL | K05115 | -3.0848793 | 3.518550409 | 11.411974 | 0.000729723 | 0.0074832 | 0.4630872 | 2.8590604 |
| ENSG00000145431 | PDGFC | K05450 | -3.0848791 | 3.518550409 | 11.411739 | 0.000729815 | 0.0074832 | 0.4956522 | 2.9971014 |
| ENSG00000170264 | FAM161A | K16772 | -3.0848791 | 3.518550409 | 11.411665 | 0.000729844 | 0.0074832 | 0.5837989 | 3.0740223 |
| ENSG00000167895 | TMC8 | - | -3.084879 | 3.518550409 | 11.411647 | 0.000729851 | 0.0074832 | 0.362259 | 3.1432507 |
| ENSG00000167617 | CDC42EP5 | - | -3.0848789 | 3.518550409 | 11.411516 | 0.000729902 | 0.0074832 | 0.3310811 | 2.5202703 |
| ENSG00000144736 | SHQ1 | K14764 | -3.0848787 | 3.518550409 | 11.411305 | 0.000729985 | 0.0074832 | 0.5875217 | 3 |
| ENSG00000176401 | EID2B | - | -3.0848786 | 3.518550409 | 11.411202 | 0.000730026 | 0.0074832 | 0.4285714 | 2.6149068 |
| ENSG00000179299 | NSUN7 | - | -3.0848785 | 3.518550409 | 11.411003 | 0.000730104 | 0.0074832 | 0.4860724 | 3.0292479 |
| ENSG00000142686 | C1orf216 | - | -3.0848784 | 3.518550409 | 11.410899 | 0.000730145 | 0.0074832 | 0.5065502 | 2.6899563 |
| ENSG00000144724 | PTPRG | K16667 | -3.0848783 | 3.518550409 | 11.410871 | 0.000730156 | 0.0074832 | 0.5204152 | 2.9446367 |
| ENSG00000179902 | C1orf194 | - | -3.0848783 | 3.518550409 | 11.410865 | 0.000730158 | 0.0074832 | 0.5266272 | 3.1301775 |
| ENSG00000144668 | ITGA9 | K06585 | -3.0848783 | 3.518550409 | 11.410853 | 0.000730163 | 0.0074832 | 0.4657005 | 2.9217391 |
| ENSG00000144063 | MALL | - | -3.0848783 | 3.518550409 | 11.410835 | 0.00073017 | 0.0074832 | 0.3464052 | 3.4836601 |
| ENSG00000143942 | CHAC2 | - | -3.0848783 | 3.518550409 | 11.410818 | 0.000730177 | 0.0074832 | 0.4836957 | 3.1684783 |
| ENSG00000186130 | ZBTB6 | K10493 | -3.0848783 | 3.518550409 | 11.410791 | 0.000730187 | 0.0074832 | 0.5495283 | 2.9316038 |
| ENSG00000178537 | SLC25A20 | K15109 | -3.0848782 | 3.518550409 | 11.410739 | 0.000730208 | 0.0074832 | 0.3322259 | 3.0166113 |
| ENSG00000180304 | OAZ2 | K16613 | -3.0848782 | 3.518550409 | 11.410728 | 0.000730212 | 0.0074832 | 0.4603175 | 2.973545 |
| ENSG00000181513 | ACBD4 | - | -3.0848781 | 3.518550409 | 11.410591 | 0.000730266 | 0.0074832 | 0.3870968 | 2.8504399 |
| ENSG00000184619 | KRBA2 | - | -3.084878 | 3.518550409 | 11.410454 | 0.00073032 | 0.0074832 | 0.5487805 | 3.0426829 |
| ENSG00000141428 | C18orf21 | - | -3.0848779 | 3.518550409 | 11.410414 | 0.000730336 | 0.0074832 | 0.4454545 | 2.9409091 |
| ENSG00000186074 | CD300LF | K20395 | -3.0848779 | 3.518550409 | 11.410316 | 0.000730374 | 0.0074832 | 0.5180328 | 2.9016393 |
| ENSG00000140416 | TPM1 | K10373 | -3.0848775 | 3.518550409 | 11.409973 | 0.000730509 | 0.0074832 | 0.7239264 | 2.892638 |
| ENSG00000139083 | ETV6 | K03211 | -3.0848772 | 3.518550409 | 11.409533 | 0.000730682 | 0.0074832 | 0.5221239 | 3.1393805 |
| ENSG00000138658 | ZGRF1 | - | -3.084877 | 3.518550409 | 11.40938 | 0.000730742 | 0.0074832 | 0.5594106 | 2.9529468 |
| ENSG00000138449 | SLC40A1 | K14685 | -3.0848769 | 3.518550409 | 11.409227 | 0.000730802 | 0.0074832 | 0.3992995 | 2.9369527 |
| ENSG00000137880 | GCHFR | - | -3.0848765 | 3.518550409 | 11.408839 | 0.000730955 | 0.0074832 | 0.4761905 | 3.0952381 |
| ENSG00000131471 | AOC3 | K00276 | -3.0848762 | 3.518550409 | 11.408451 | 0.000731108 | 0.0074832 | 0.4220183 | 3.0222805 |
| ENSG00000123607 | TTC21B | K19673 | -3.0848758 | 3.518550409 | 11.407988 | 0.00073129 | 0.0074832 | 0.5022796 | 3.0942249 |
| ENSG00000119787 | ATL2 | K17339 | -3.0848754 | 3.518550409 | 11.407525 | 0.000731472 | 0.0074832 | 0.5248714 | 3.0325901 |
| ENSG00000112769 | LAMA4 | K06241 | -3.0848752 | 3.518550409 | 11.407334 | 0.000731547 | 0.0074832 | 0.5216676 | 2.8628634 |
| ENSG00000115594 | IL1R1 | K04386 | -3.0848751 | 3.518550409 | 11.407198 | 0.000731601 | 0.0074832 | 0.486819 | 3.1915641 |
| ENSG00000110719 | TCIRG1 | K02154 | -3.084875 | 3.518550409 | 11.407075 | 0.000731649 | 0.0074832 | 0.3759036 | 3.0819277 |
| ENSG00000116698 | SMG7 | K14409 | -3.0848749 | 3.518550409 | 11.407062 | 0.000731655 | 0.0074832 | 0.4949066 | 2.9550085 |
| ENSG00000109072 | VTN | K06251 | -3.0848747 | 3.518550409 | 11.406816 | 0.000731751 | 0.0074832 | 0.5209205 | 3.0083682 |
| ENSG00000107874 | CUEDC2 | - | -3.0848745 | 3.518550409 | 11.406557 | 0.000731853 | 0.0074832 | 0.5400697 | 2.8919861 |
| ENSG00000107816 | LZTS2 | - | -3.0848743 | 3.518550409 | 11.406299 | 0.000731955 | 0.0074832 | 0.5022422 | 2.6562033 |
| ENSG00000103994 | ZNF106 | - | -3.084874 | 3.518550409 | 11.406039 | 0.000732057 | 0.0074832 | 0.575146 | 2.7419012 |
| ENSG00000102096 | PIM2 | K08806 | -3.0848738 | 3.518550409 | 11.40578 | 0.000732159 | 0.0074832 | 0.3890675 | 2.9614148 |
| ENSG00000101608 | MYL12A | K12757 | -3.0848736 | 3.518550409 | 11.40555 | 0.00073225 | 0.0074832 | 0.6440678 | 3.0338983 |
| ENSG00000092841 | MYL6 | K12751 | -3.0848732 | 3.518550409 | 11.405136 | 0.000732413 | 0.0074832 | 0.5420168 | 2.8319328 |
| ENSG00000089820 | ARHGAP4 | K20122 | -3.0848729 | 3.518550409 | 11.404723 | 0.000732576 | 0.0074832 | 0.494929 | 2.8407708 |
| ENSG00000085491 | SLC25A24 | K14684 | -3.0848725 | 3.518550409 | 11.404309 | 0.000732739 | 0.0074832 | 0.4716981 | 3.0104822 |
| ENSG00000074660 | SCARF1 | - | -3.0848724 | 3.518550409 | 11.404214 | 0.000732777 | 0.0074832 | 0.4240964 | 2.4120482 |
| ENSG00000070010 | UFD1L | K14016 | -3.0848722 | 3.518550409 | 11.404038 | 0.000732846 | 0.0074832 | 0.4918567 | 2.9837134 |
| ENSG00000075624 | ACTB | K05692 | -3.0848721 | 3.518550409 | 11.403896 | 0.000732902 | 0.0074832 | 0.4933333 | 2.9226667 |
| ENSG00000066739 | ATG2B | K17906 | -3.0848721 | 3.518550409 | 11.403824 | 0.000732931 | 0.0074832 | 0.5250241 | 2.9595765 |
| ENSG00000130311 | DDA1 | K11792 | -3.0802902 | 5.928787393 | 10.405277 | 0.001256557 | 0.0097761 | 0.5686275 | 3.0588235 |
| ENSG00000183160 | TMEM119 | - | -3.0257592 | 3.475627011 | 11.290725 | 0.000778953 | 0.0074949 | 0.4628975 | 2.5194346 |
| ENSG00000169896 | ITGAM | K06461 | -3.025759 | 3.475627011 | 11.290488 | 0.000779052 | 0.0074949 | 0.4787511 | 2.8811795 |
| ENSG00000168918 | INPP5D | K03084 | -3.0257588 | 3.475627011 | 11.290252 | 0.000779151 | 0.0074949 | 0.5155593 | 2.980656 |
| ENSG00000160255 | ITGB2 | K06464 | -3.0257585 | 3.475627011 | 11.290001 | 0.000779257 | 0.0074949 | 0.4655397 | 2.7542263 |
| ENSG00000157168 | NRG1 | K05455 | -3.0257581 | 3.475627011 | 11.289596 | 0.000779426 | 0.0074949 | 0.5203366 | 2.772791 |
| ENSG00000143341 | HMCN1 | K17341 | -3.0257578 | 3.475627011 | 11.289192 | 0.000779596 | 0.0074949 | 0.4784383 | 2.7705413 |
| ENSG00000135637 | CCDC142 | - | -3.0257574 | 3.475627011 | 11.288787 | 0.000779766 | 0.0074949 | 0.3946667 | 2.8213333 |
| ENSG00000132514 | CLEC10A | K06721 | -3.025757 | 3.475627011 | 11.288383 | 0.000779936 | 0.0074949 | 0.5 | 2.9113924 |
| ENSG00000053702 | NRIP2 | K13215 | -3.0257568 | 3.475627011 | 11.288239 | 0.000779996 | 0.0074949 | 0.4697509 | 2.8327402 |
| ENSG00000065621 | GSTO2 | K00799 | -3.0257568 | 3.475627011 | 11.288152 | 0.000780033 | 0.0074949 | 0.436214 | 3.2674897 |
| ENSG00000122122 | SASH3 | - | -3.0257567 | 3.475627011 | 11.288065 | 0.000780069 | 0.0074949 | 0.5657895 | 2.7552632 |
| ENSG00000129226 | CD68 | K06501 | -3.0257566 | 3.475627011 | 11.287978 | 0.000780106 | 0.0074949 | 0.4350282 | 2.6553672 |
| ENSG00000173409 | ARV1 | - | -3.0245154 | 6.212807593 | 10.439324 | 0.001233604 | 0.0096352 | 0.4123711 | 3.2302406 |
| ENSG00000154654 | NCAM2 | K06491 | -3.0092049 | 3.463676172 | 11.263075 | 0.000790641 | 0.0074949 | 0.5376344 | 2.9020311 |
| ENSG00000136425 | CIB2 | - | -3.0092048 | 3.463676172 | 11.262932 | 0.000790702 | 0.0074949 | 0.5828877 | 3.2032086 |
| ENSG00000138642 | HERC6 | - | -3.0092047 | 3.463676172 | 11.262912 | 0.000790711 | 0.0074949 | 0.462818 | 3.0117417 |
| ENSG00000140386 | SCAPER | - | -3.0092047 | 3.463676172 | 11.262891 | 0.00079072 | 0.0074949 | 0.5414286 | 2.8935714 |
| ENSG00000141469 | SLC14A1 | K08716 | -3.0092047 | 3.463676172 | 11.26287 | 0.000790728 | 0.0074949 | 0.352809 | 2.9370787 |
| ENSG00000147481 | SNTG1 | - | -3.0092047 | 3.463676172 | 11.262862 | 0.000790732 | 0.0074949 | 0.4874275 | 2.9709865 |
| ENSG00000155755 | TMEM237 | - | -3.0092046 | 3.463676172 | 11.262738 | 0.000790785 | 0.0074949 | 0.5171569 | 2.9607843 |
| ENSG00000146453 | PNLDC1 | - | -3.0092045 | 3.463676172 | 11.262648 | 0.000790823 | 0.0074949 | 0.4689266 | 3.2485876 |
| ENSG00000143858 | SYT2 | K19902 | -3.0092044 | 3.463676172 | 11.262545 | 0.000790867 | 0.0074949 | 0.4844869 | 2.9761337 |
| ENSG00000136238 | RAC1 | K04392 | -3.0092043 | 3.463676172 | 11.262462 | 0.000790902 | 0.0074949 | 0.4312796 | 3 |
| ENSG00000145864 | GABRB2 | K05181 | -3.0092043 | 3.463676172 | 11.262434 | 0.000790915 | 0.0074949 | 0.4589844 | 3.2304688 |
| ENSG00000156127 | BATF | K09034 | -3.0092042 | 3.463676172 | 11.2624 | 0.000790929 | 0.0074949 | 0.576 | 2.848 |
| ENSG00000156162 | DPY19L4 | - | -3.0092042 | 3.463676172 | 11.262326 | 0.00079096 | 0.0074949 | 0.4301521 | 3.3526971 |
| ENSG00000157823 | AP3S2 | K12399 | -3.0092041 | 3.463676172 | 11.262252 | 0.000790992 | 0.0074949 | 0.4634146 | 3.1707317 |
| ENSG00000145476 | CYP4V2 | K07427 | -3.0092041 | 3.463676172 | 11.262219 | 0.000791006 | 0.0074949 | 0.4590476 | 3.2038095 |
| ENSG00000135446 | CDK4 | K02089 | -3.0092038 | 3.463676172 | 11.261991 | 0.000791103 | 0.0074949 | 0.4158416 | 2.990099 |
| ENSG00000135314 | KHDC1 | - | -3.0092038 | 3.463676172 | 11.261914 | 0.000791136 | 0.0074949 | 0.4261603 | 3.1181435 |
| ENSG00000157837 | SPPL3 | K09598 | -3.0092037 | 3.463676172 | 11.261877 | 0.000791152 | 0.0074949 | 0.375 | 3.0286458 |
| ENSG00000135127 | CCDC64 | K16756 | -3.0092037 | 3.463676172 | 11.261836 | 0.000791169 | 0.0074949 | 0.609075 | 2.8027923 |
| ENSG00000131378 | RFTN1 | - | -3.0092036 | 3.463676172 | 11.261758 | 0.000791202 | 0.0074949 | 0.5380623 | 2.7456747 |
| ENSG00000130592 | LSP1 | K14957 | -3.0092035 | 3.463676172 | 11.26168 | 0.000791236 | 0.0074949 | 0.5267666 | 2.6188437 |
| ENSG00000128604 | IRF5 | K09446 | -3.0092034 | 3.463676172 | 11.261602 | 0.000791269 | 0.0074949 | 0.4513619 | 3.0544747 |
| ENSG00000128045 | RASL11B | K07853 | -3.0092034 | 3.463676172 | 11.26158 | 0.000791278 | 0.0074949 | 0.4354839 | 2.875 |
| ENSG00000160972 | PPP1R16A | K17458 | -3.0092033 | 3.463676172 | 11.261503 | 0.000791311 | 0.0074949 | 0.4734848 | 2.719697 |
| ENSG00000117602 | RCAN3 | K17905 | -3.0092031 | 3.463676172 | 11.261299 | 0.000791398 | 0.0074949 | 0.6182573 | 3 |
| ENSG00000162378 | ZYG11B | K10350 | -3.009203 | 3.463676172 | 11.261128 | 0.000791471 | 0.0074949 | 0.4596774 | 3.0040323 |
| ENSG00000116138 | DNAJC16 | K09536 | -3.0092029 | 3.463676172 | 11.261017 | 0.000791518 | 0.0074949 | 0.5076726 | 3.2672634 |
| ENSG00000163584 | RPL22L1 | K02891 | -3.0092026 | 3.463676172 | 11.260754 | 0.000791631 | 0.0074949 | 0.5774648 | 3.2535211 |
| ENSG00000115318 | LOXL3 | K00280 | -3.0092026 | 3.463676172 | 11.260735 | 0.000791639 | 0.0074949 | 0.4488712 | 2.7715803 |
| ENSG00000110446 | SLC15A3 | K14638 | -3.0092023 | 3.463676172 | 11.260453 | 0.000791759 | 0.0074949 | 0.3184165 | 3.0051635 |
| ENSG00000163661 | PTX3 | - | -3.0092022 | 3.463676172 | 11.26038 | 0.00079179 | 0.0074949 | 0.4750656 | 2.808399 |
| ENSG00000105948 | TTC26 | K19685 | -3.009202 | 3.463676172 | 11.260171 | 0.000791879 | 0.0074949 | 0.5036101 | 3.2075812 |
| ENSG00000163879 | DNALI1 | K10410 | -3.0092019 | 3.463676172 | 11.260006 | 0.00079195 | 0.0074949 | 0.5285714 | 2.9214286 |
| ENSG00000103546 | SLC6A2 | K05035 | -3.0092018 | 3.463676172 | 11.259889 | 0.000791999 | 0.0074949 | 0.3630573 | 3.2324841 |
| ENSG00000164062 | APEH | K01303 | -3.0092015 | 3.463676172 | 11.259632 | 0.000792109 | 0.0074949 | 0.4640434 | 2.9375848 |
| ENSG00000102984 | ZNF821 | - | -3.0092012 | 3.463676172 | 11.259364 | 0.000792223 | 0.0074949 | 0.5606796 | 2.788835 |
| ENSG00000164294 | GPX8 | K00432 | -3.0092011 | 3.463676172 | 11.259259 | 0.000792268 | 0.0074949 | 0.3971292 | 3.3014354 |
| ENSG00000181031 | RPH3AL | K19939 | -3.0092008 | 3.463676172 | 11.258924 | 0.000792411 | 0.0074949 | 0.4507937 | 2.6952381 |
| ENSG00000165195 | PIGA | K03857 | -3.0092008 | 3.463676172 | 11.258885 | 0.000792428 | 0.0074949 | 0.4235537 | 3.0082645 |
| ENSG00000101335 | MYL9 | K12755 | -3.0092007 | 3.463676172 | 11.258839 | 0.000792448 | 0.0074949 | 0.6104651 | 3.0290698 |
| ENSG00000180917 | CMTR2 | K14590 | -3.0092007 | 3.463676172 | 11.258833 | 0.00079245 | 0.0074949 | 0.4545455 | 3.1402597 |
| ENSG00000059145 | UNKL | - | -3.0092007 | 3.463676172 | 11.258788 | 0.000792469 | 0.0074949 | 0.4502046 | 2.6780355 |
| ENSG00000072071 | ADGRL1 | K04592 | -3.0092006 | 3.463676172 | 11.258717 | 0.0007925 | 0.0074949 | 0.4620081 | 2.9151967 |
| ENSG00000075461 | CACNG4 | K04869 | -3.0092005 | 3.463676172 | 11.258646 | 0.00079253 | 0.0074949 | 0.4495413 | 2.969419 |
| ENSG00000177879 | AP3S1 | K12399 | -3.0092005 | 3.463676172 | 11.258627 | 0.000792538 | 0.0074949 | 0.4870466 | 3.0362694 |
| ENSG00000100239 | PPP6R2 | K15500 | -3.0092005 | 3.463676172 | 11.258601 | 0.000792549 | 0.0074949 | 0.5217391 | 2.7101449 |
| ENSG00000085117 | CD82 | K06509 | -3.0092005 | 3.463676172 | 11.258575 | 0.00079256 | 0.0074949 | 0.4082397 | 3.0187266 |
| ENSG00000041353 | RAB27B | K07886 | -3.0092004 | 3.463676172 | 11.258542 | 0.000792574 | 0.0074949 | 0.5321101 | 2.9816514 |
| ENSG00000174600 | CMKLR1 | K04245 | -3.0092004 | 3.463676172 | 11.258529 | 0.00079258 | 0.0074949 | 0.3780161 | 3.1930295 |
| ENSG00000165868 | HSPA12A | - | -3.0092004 | 3.463676172 | 11.258512 | 0.000792587 | 0.0074949 | 0.482963 | 2.9866667 |
| ENSG00000089351 | GRAMD1A | - | -3.0092004 | 3.463676172 | 11.258504 | 0.000792591 | 0.0074949 | 0.511772 | 2.8946716 |
| ENSG00000183134 | PTGDR2 | K06715 | -3.0092004 | 3.463676172 | 11.258494 | 0.000792595 | 0.0074949 | 0.3113924 | 2.9291139 |
| ENSG00000099377 | HSD3B7 | K12408 | -3.0092003 | 3.463676172 | 11.258433 | 0.000792621 | 0.0074949 | 0.3766938 | 3.0135501 |
| ENSG00000171724 | VAT1L | - | -3.0092003 | 3.463676172 | 11.258432 | 0.000792622 | 0.0074949 | 0.4844869 | 2.8520286 |
| ENSG00000099795 | NDUFB7 | K03963 | -3.0092002 | 3.463676172 | 11.258362 | 0.000792651 | 0.0074949 | 0.4671533 | 3.2189781 |
| ENSG00000169750 | RAC3 | K07861 | -3.0092002 | 3.463676172 | 11.258334 | 0.000792663 | 0.0074949 | 0.421875 | 3.0104167 |
| ENSG00000011677 | GABRA3 | K05175 | -3.0092002 | 3.463676172 | 11.258297 | 0.000792679 | 0.0074949 | 0.4654472 | 3.0711382 |
| ENSG00000169429 | CXCL8 | K10030 | -3.0092001 | 3.463676172 | 11.258236 | 0.000792705 | 0.0074949 | 0.3838384 | 3 |
| ENSG00000167664 | TMIGD2 | K16668 | -3.0092 | 3.463676172 | 11.258139 | 0.000792747 | 0.0074949 | 0.4007092 | 2.7765957 |
| ENSG00000184163 | FAM132A | K21410 | -3.0091999 | 3.463676172 | 11.258064 | 0.000792778 | 0.0074949 | 0.3807947 | 2.7119205 |
| ENSG00000011422 | PLAUR | K03985 | -3.0091999 | 3.463676172 | 11.258051 | 0.000792784 | 0.0074949 | 0.480597 | 2.6477612 |
| ENSG00000186288 | PABPC1L2A | K13126 | -3.0091999 | 3.463676172 | 11.257975 | 0.000792817 | 0.0074949 | 0.475 | 3.06 |
| ENSG00000196177 | ACADSB | K09478 | -3.0091998 | 3.463676172 | 11.257885 | 0.000792855 | 0.0074949 | 0.4675926 | 2.9097222 |
| ENSG00000005108 | THSD7A | - | -3.0091997 | 3.463676172 | 11.257806 | 0.000792889 | 0.0074949 | 0.4876282 | 2.7718769 |
| ENSG00000120992 | LYPLA1 | K06128 | -2.5192292 | 3.124697212 | 10.471141 | 0.001212538 | 0.0095882 | 0.3913043 | 2.7521739 |
| ENSG00000122490 | PQLC1 | - | -2.5192289 | 3.124697212 | 10.470946 | 0.001212666 | 0.0095882 | 0.3616236 | 3.1549816 |
| ENSG00000119408 | NEK6 | K20875 | -2.5192287 | 3.124697212 | 10.470829 | 0.001212743 | 0.0095882 | 0.426513 | 3.092219 |
| ENSG00000122687 | FTSJ2 | K02427 | -2.5192285 | 3.124697212 | 10.470751 | 0.001212794 | 0.0095882 | 0.398374 | 2.9308943 |
| ENSG00000122986 | HVCN1 | - | -2.5192281 | 3.124697212 | 10.470556 | 0.001212922 | 0.0095882 | 0.4102564 | 3.3186813 |
| ENSG00000118094 | TREH | K01194 | -2.5192281 | 3.124697212 | 10.470517 | 0.001212948 | 0.0095882 | 0.4888508 | 3.1578045 |
| ENSG00000124256 | ZBP1 | K12965 | -2.5192278 | 3.124697212 | 10.470361 | 0.00121305 | 0.0095882 | 0.4708625 | 2.6456876 |
| ENSG00000117620 | SLC35A3 | K15272 | -2.5192275 | 3.124697212 | 10.470205 | 0.001213152 | 0.0095882 | 0.4114441 | 3.1171662 |
| ENSG00000124493 | GRM4 | K04607 | -2.5192274 | 3.124697212 | 10.470166 | 0.001213178 | 0.0095882 | 0.4133772 | 2.9901316 |
| ENSG00000125388 | GRK4 | K08291 | -2.519227 | 3.124697212 | 10.469971 | 0.001213306 | 0.0095882 | 0.4982699 | 3.1107266 |
| ENSG00000117560 | FASLG | K04389 | -2.5192268 | 3.124697212 | 10.469859 | 0.00121338 | 0.0095882 | 0.3843416 | 3.113879 |
| ENSG00000125735 | TNFSF14 | K05477 | -2.5192267 | 3.124697212 | 10.469776 | 0.001213434 | 0.0095882 | 0.3875 | 2.9 |
| ENSG00000125898 | FAM110A | - | -2.5192263 | 3.124697212 | 10.469582 | 0.001213562 | 0.0095882 | 0.379661 | 2.5898305 |
| ENSG00000117151 | CTBS | K12310 | -2.5192262 | 3.124697212 | 10.469514 | 0.001213607 | 0.0095882 | 0.4883117 | 3.0675325 |
| ENSG00000127526 | SLC35E1 | K15283 | -2.5192259 | 3.124697212 | 10.469387 | 0.00121369 | 0.0095882 | 0.3195122 | 3.0170732 |
| ENSG00000128203 | ASPHD2 | - | -2.5192256 | 3.124697212 | 10.469193 | 0.001213817 | 0.0095882 | 0.4281843 | 3.0135501 |
| ENSG00000116685 | LOC102724984 | - | -2.5192255 | 3.124697212 | 10.469168 | 0.001213834 | 0.0095882 | 0.3553223 | 2.9055472 |
| ENSG00000128886 | ELL3 | K15183 | -2.5192252 | 3.124697212 | 10.468999 | 0.001213945 | 0.0095882 | 0.5516373 | 2.9622166 |
| ENSG00000116260 | QSOX1 | K10758 | -2.5192251 | 3.124697212 | 10.468957 | 0.001213973 | 0.0095882 | 0.3842035 | 2.9558233 |
| ENSG00000130164 | LDLR | K12473 | -2.5192248 | 3.124697212 | 10.468804 | 0.001214073 | 0.0095882 | 0.5094937 | 2.7267932 |
| ENSG00000116151 | MORN1 | - | -2.5192247 | 3.124697212 | 10.468746 | 0.001214111 | 0.0095882 | 0.4124748 | 2.7806841 |
| ENSG00000131370 | SH3BP5 | - | -2.5192245 | 3.124697212 | 10.46861 | 0.0012142 | 0.0095882 | 0.621978 | 2.6945055 |
| ENSG00000116133 | DHCR24 | K09828 | -2.5192243 | 3.124697212 | 10.468535 | 0.00121425 | 0.0095882 | 0.4108527 | 3.3352713 |
| ENSG00000131446 | MGAT1 | K00726 | -2.5192241 | 3.124697212 | 10.468416 | 0.001214328 | 0.0095882 | 0.4089888 | 3.1797753 |
| ENSG00000115956 | PLEK | K19993 | -2.5192239 | 3.124697212 | 10.468324 | 0.001214389 | 0.0095882 | 0.5 | 3.1028571 |
| ENSG00000138771 | SHROOM3 | K18625 | -2.5192238 | 3.124697212 | 10.468258 | 0.001214432 | 0.0095882 | 0.507014 | 2.6853707 |
| ENSG00000131697 | NPHP4 | K16478 | -2.5192238 | 3.124697212 | 10.468222 | 0.001214455 | 0.0095882 | 0.4284712 | 2.9172511 |
| ENSG00000115648 | MLPH | - | -2.5192235 | 3.124697212 | 10.468112 | 0.001214528 | 0.0095882 | 0.5633333 | 2.69 |
| ENSG00000140006 | WDR89 | - | -2.5192234 | 3.124697212 | 10.468057 | 0.001214564 | 0.0095882 | 0.5348837 | 2.8604651 |
| ENSG00000132016 | C19orf57 | - | -2.5192234 | 3.124697212 | 10.468028 | 0.001214583 | 0.0095882 | 0.5254491 | 2.4266467 |
| ENSG00000138744 | NAAA | K13720 | -2.5192232 | 3.124697212 | 10.467918 | 0.001214656 | 0.0095882 | 0.4094708 | 3.0362117 |
| ENSG00000115641 | FHL2 | K14380 | -2.5192232 | 3.124697212 | 10.467901 | 0.001214666 | 0.0095882 | 0.4455696 | 2.9164557 |
| ENSG00000140488 | CELF6 | K13207 | -2.5192231 | 3.124697212 | 10.467857 | 0.001214695 | 0.0095882 | 0.3638254 | 2.6548857 |
| ENSG00000132305 | IMMT | K17785 | -2.519223 | 3.124697212 | 10.467834 | 0.00121471 | 0.0095882 | 0.525066 | 2.817942 |
| ENSG00000115112 | TFCP2L1 | K09275 | -2.5192228 | 3.124697212 | 10.46769 | 0.001214805 | 0.0095882 | 0.5114823 | 3.0584551 |
| ENSG00000142694 | EVA1B | - | -2.5192227 | 3.124697212 | 10.467657 | 0.001214827 | 0.0095882 | 0.5393939 | 2.8545455 |
| ENSG00000133401 | PDZD2 | - | -2.5192227 | 3.124697212 | 10.46764 | 0.001214838 | 0.0095882 | 0.5051074 | 2.5473758 |
| ENSG00000138382 | METTL5 | - | -2.5192225 | 3.124697212 | 10.467578 | 0.001214879 | 0.0095882 | 0.4508197 | 3.057377 |
| ENSG00000113916 | BCL6 | K15618 | -2.5192224 | 3.124697212 | 10.467478 | 0.001214944 | 0.0095882 | 0.4830028 | 2.8427762 |
| ENSG00000143190 | POU2F1 | K09364 | -2.5192223 | 3.124697212 | 10.467457 | 0.001214958 | 0.0095882 | 0.502611 | 2.4060052 |
| ENSG00000133800 | LYVE1 | K19012 | -2.5192223 | 3.124697212 | 10.467447 | 0.001214965 | 0.0095882 | 0.4844721 | 2.8540373 |
| ENSG00000113070 | HBEGF | K08523 | -2.519222 | 3.124697212 | 10.467267 | 0.001215083 | 0.0095882 | 0.3894231 | 2.8990385 |
| ENSG00000143891 | GALM | K01785 | -2.5192219 | 3.124697212 | 10.467257 | 0.00121509 | 0.0095882 | 0.4590643 | 2.9678363 |
| ENSG00000134216 | CHIA | K01183 | -2.5192219 | 3.124697212 | 10.467253 | 0.001215093 | 0.0095882 | 0.4663866 | 2.9264706 |
| ENSG00000137393 | RNF144B | K11975 | -2.5192219 | 3.124697212 | 10.467237 | 0.001215103 | 0.0095882 | 0.3927393 | 2.8052805 |
| ENSG00000134812 | GIF | K14615 | -2.5192216 | 3.124697212 | 10.46706 | 0.00121522 | 0.0095882 | 0.4820144 | 2.8369305 |
| ENSG00000145919 | BOD1 | - | -2.5192216 | 3.124697212 | 10.467057 | 0.001215221 | 0.0095882 | 0.4378378 | 2.4810811 |
| ENSG00000112964 | GHR | K05080 | -2.5192216 | 3.124697212 | 10.467055 | 0.001215223 | 0.0095882 | 0.5565891 | 2.9503876 |
| ENSG00000137193 | PIM1 | K04702 | -2.5192213 | 3.124697212 | 10.466897 | 0.001215327 | 0.0095882 | 0.4632588 | 3.1118211 |
| ENSG00000135070 | ISCA1 | K13628 | -2.5192212 | 3.124697212 | 10.466866 | 0.001215347 | 0.0095882 | 0.4883721 | 2.8062016 |
| ENSG00000148690 | FRA10AC1 | K13121 | -2.5192212 | 3.124697212 | 10.466857 | 0.001215353 | 0.0095882 | 0.615873 | 3.2190476 |
| ENSG00000112115 | IL17A | K05489 | -2.5192212 | 3.124697212 | 10.466844 | 0.001215362 | 0.0095882 | 0.4451613 | 2.9225806 |
| ENSG00000135951 | TSGA10 | - | -2.5192208 | 3.124697212 | 10.466673 | 0.001215474 | 0.0095882 | 0.6432665 | 2.9240688 |
| ENSG00000148834 | GSTO1 | K00799 | -2.5192208 | 3.124697212 | 10.466658 | 0.001215484 | 0.0095882 | 0.4854772 | 3.1784232 |
| ENSG00000110697 | PITPNM1 | - | -2.5192208 | 3.124697212 | 10.466632 | 0.001215501 | 0.0095882 | 0.4694534 | 2.7749196 |
| ENSG00000136153 | LMO7 | K06084 | -2.5192205 | 3.124697212 | 10.46648 | 0.001215601 | 0.0095882 | 0.5905276 | 2.9478417 |
| ENSG00000149292 | TTC12 | - | -2.5192204 | 3.124697212 | 10.466458 | 0.001215616 | 0.0095882 | 0.5095628 | 2.8879781 |
| ENSG00000136869 | TLR4 | K10160 | -2.5192201 | 3.124697212 | 10.466286 | 0.001215729 | 0.0095882 | 0.4839094 | 3.1537545 |
| ENSG00000152904 | GGPS1 | K00804 | -2.5192201 | 3.124697212 | 10.466259 | 0.001215747 | 0.0095882 | 0.54 | 3.2533333 |
| ENSG00000110046 | ATG2A | K17906 | -2.51922 | 3.124697212 | 10.466214 | 0.001215776 | 0.0095882 | 0.4649123 | 2.8529412 |
| ENSG00000152939 | MARVELD2 | K17291 | -2.5192197 | 3.124697212 | 10.466059 | 0.001215878 | 0.0095882 | 0.4928315 | 3.1577061 |
| ENSG00000154975 | CA10 | - | -2.5192193 | 3.124697212 | 10.46586 | 0.001216009 | 0.0095882 | 0.4817073 | 3.1036585 |
| ENSG00000108947 | EFNB3 | K05463 | -2.5192192 | 3.124697212 | 10.465811 | 0.001216042 | 0.0095882 | 0.35 | 2.6588235 |
| ENSG00000157502 | MUM1L1 | - | -2.5192191 | 3.124697212 | 10.46575 | 0.001216082 | 0.0095882 | 0.6077586 | 2.9497126 |
| ENSG00000157315 | TMED6 | K20351 | -2.5192191 | 3.124697212 | 10.46572 | 0.001216101 | 0.0095882 | 0.4791667 | 3.1958333 |
| ENSG00000157111 | TMEM171 | - | -2.519219 | 3.124697212 | 10.46569 | 0.001216121 | 0.0095882 | 0.4351852 | 2.7777778 |
| ENSG00000157005 | SST | K05237 | -2.5192189 | 3.124697212 | 10.465661 | 0.001216141 | 0.0095882 | 0.4741379 | 2.75 |
| ENSG00000157796 | WDR19 | K19671 | -2.5192187 | 3.124697212 | 10.465554 | 0.001216211 | 0.0095882 | 0.4836066 | 3.0298063 |
| ENSG00000106927 | AMBP | - | -2.5192185 | 3.124697212 | 10.465407 | 0.001216307 | 0.0095882 | 0.4744318 | 2.8607955 |
| ENSG00000158062 | UBXN11 | - | -2.5192183 | 3.124697212 | 10.465297 | 0.00121638 | 0.0095882 | 0.4826923 | 2.8461538 |
| ENSG00000158473 | CD1D | K06448 | -2.5192178 | 3.124697212 | 10.46504 | 0.001216549 | 0.0095882 | 0.4268657 | 3.080597 |
| ENSG00000162390 | ACOT11 | K12417 | -2.5192178 | 3.124697212 | 10.46504 | 0.001216549 | 0.0095882 | 0.4711697 | 2.9159802 |
| ENSG00000105576 | TNPO2 | K18727 | -2.5192177 | 3.124697212 | 10.465003 | 0.001216573 | 0.0095882 | 0.4804905 | 3.0479376 |
| ENSG00000162458 | FBLIM1 | - | -2.5192174 | 3.124697212 | 10.464816 | 0.001216697 | 0.0095882 | 0.3957219 | 2.7192513 |
| ENSG00000158488 | CD1E | K06448 | -2.5192173 | 3.124697212 | 10.464783 | 0.001216719 | 0.0095882 | 0.3943299 | 3.064433 |
| ENSG00000160326 | SLC2A6 | K08144 | -2.5192171 | 3.124697212 | 10.464654 | 0.001216803 | 0.0095882 | 0.3116371 | 2.9447732 |
| ENSG00000104883 | PEX11G | K13353 | -2.5192169 | 3.124697212 | 10.4646 | 0.001216839 | 0.0095882 | 0.3651452 | 2.9087137 |
| ENSG00000162777 | DENND2D | K20161 | -2.5192169 | 3.124697212 | 10.464592 | 0.001216844 | 0.0095882 | 0.4543524 | 3.1677282 |
| ENSG00000159674 | SPON2 | - | -2.5192168 | 3.124697212 | 10.464526 | 0.001216888 | 0.0095882 | 0.4531722 | 2.776435 |
| ENSG00000163251 | FZD5 | K02375 | -2.5192165 | 3.124697212 | 10.464369 | 0.001216991 | 0.0095882 | 0.3401709 | 2.991453 |
| ENSG00000160179 | ABCG1 | K05679 | -2.5192163 | 3.124697212 | 10.464269 | 0.001217057 | 0.0095882 | 0.3907767 | 2.9538835 |
| ENSG00000104728 | ARHGEF10 | K16727 | -2.5192162 | 3.124697212 | 10.464196 | 0.001217105 | 0.0095882 | 0.5200877 | 2.8429511 |
| ENSG00000163263 | C1orf189 | - | -2.5192161 | 3.124697212 | 10.464146 | 0.001217138 | 0.0095882 | 0.4653465 | 3.2574257 |
| ENSG00000163464 | CXCR1 | K04175 | -2.5192157 | 3.124697212 | 10.463922 | 0.001217285 | 0.0095882 | 0.3485714 | 3.24 |
| ENSG00000103326 | CAPN15 | K08582 | -2.5192154 | 3.124697212 | 10.463791 | 0.001217372 | 0.0095882 | 0.4152855 | 2.7117864 |
| ENSG00000163736 | PPBP | K10029 | -2.5192153 | 3.124697212 | 10.463699 | 0.001217432 | 0.0095882 | 0.453125 | 2.6875 |
| ENSG00000178597 | PSAPL1 | K12382 | -2.5192149 | 3.124697212 | 10.463488 | 0.001217572 | 0.0095882 | 0.3973129 | 2.7236084 |
| ENSG00000164040 | PGRMC2 | K17278 | -2.5192148 | 3.124697212 | 10.463476 | 0.00121758 | 0.0095882 | 0.465587 | 2.5708502 |
| ENSG00000102312 | PORCN | K00181 | -2.5192147 | 3.124697212 | 10.463387 | 0.001217638 | 0.0095882 | 0.329718 | 3.2494577 |
| ENSG00000180828 | BHLHE22 | K09086 | -2.5192146 | 3.124697212 | 10.463323 | 0.001217681 | 0.0095882 | 0.3858268 | 2.1154856 |
| ENSG00000177694 | NAALADL2 | K01301 | -2.5192145 | 3.124697212 | 10.463278 | 0.00121771 | 0.0095882 | 0.5220126 | 2.9446541 |
| ENSG00000164162 | ANAPC10 | K03357 | -2.5192144 | 3.124697212 | 10.463253 | 0.001217727 | 0.0095882 | 0.5135135 | 3.0378378 |
| ENSG00000181830 | SLC35C1 | K15279 | -2.5192142 | 3.124697212 | 10.463158 | 0.001217789 | 0.0095882 | 0.3626374 | 3.0192308 |
| ENSG00000177663 | IL17RA | K05164 | -2.5192141 | 3.124697212 | 10.463068 | 0.001217849 | 0.0095882 | 0.4688222 | 2.9110855 |
| ENSG00000164972 | C9orf24 | K18634 | -2.519214 | 3.124697212 | 10.46303 | 0.001217873 | 0.0095882 | 0.4694656 | 3.0229008 |
| ENSG00000181982 | CCDC149 | - | -2.5192139 | 3.124697212 | 10.462993 | 0.001217898 | 0.0095882 | 0.5796296 | 2.8018519 |
| ENSG00000102290 | PCDH11X | K16498 | -2.5192139 | 3.124697212 | 10.462983 | 0.001217905 | 0.0095882 | 0.5337788 | 2.8240535 |
| ENSG00000186335 | SLC36A2 | K14209 | -2.5192139 | 3.124697212 | 10.462954 | 0.001217924 | 0.0095882 | 0.3602484 | 3.068323 |
| ENSG00000182330 | PRAMEF8 | - | -2.5192136 | 3.124697212 | 10.462828 | 0.001218007 | 0.0095882 | 0.4409283 | 3.0147679 |
| ENSG00000165915 | SLC39A13 | K14719 | -2.5192136 | 3.124697212 | 10.462807 | 0.00121802 | 0.0095882 | 0.3450135 | 2.7358491 |
| ENSG00000177511 | ST8SIA3 | K06613 | -2.5192135 | 3.124697212 | 10.462744 | 0.001218062 | 0.0095882 | 0.4263158 | 3.2789474 |
| ENSG00000183513 | COA5 | K18178 | -2.5192133 | 3.124697212 | 10.462663 | 0.001218115 | 0.0095882 | 0.4459459 | 2.9054054 |
| ENSG00000186377 | CYP4X1 | K07428 | -2.5192133 | 3.124697212 | 10.462651 | 0.001218123 | 0.0095882 | 0.4381139 | 3.2770138 |
| ENSG00000186106 | ANKRD46 | - | -2.5192133 | 3.124697212 | 10.462638 | 0.001218132 | 0.0095882 | 0.4741379 | 2.9353448 |
| ENSG00000166603 | MC4R | K04202 | -2.5192133 | 3.124697212 | 10.462634 | 0.001218134 | 0.0095882 | 0.3674699 | 3.0692771 |
| ENSG00000165949 | IFI27 | - | -2.5192132 | 3.124697212 | 10.462585 | 0.001218167 | 0.0095882 | 0.2377049 | 2.1885246 |
| ENSG00000101443 | WFDC2 | - | -2.5192132 | 3.124697212 | 10.462578 | 0.001218171 | 0.0095882 | 0.4274194 | 2.3629032 |
| ENSG00000166228 | PCBD1 | K01724 | -2.5192131 | 3.124697212 | 10.462551 | 0.00121819 | 0.0095882 | 0.4615385 | 3.1346154 |
| ENSG00000183798 | EMILIN3 | - | -2.519213 | 3.124697212 | 10.462499 | 0.001218224 | 0.0095882 | 0.4477807 | 2.6501305 |
| ENSG00000177042 | TMEM80 | - | -2.5192129 | 3.124697212 | 10.46242 | 0.001218276 | 0.0095882 | 0.361991 | 2.8552036 |
| ENSG00000167207 | NOD2 | K10165 | -2.5192128 | 3.124697212 | 10.462365 | 0.001218312 | 0.0095882 | 0.4153846 | 2.9278846 |
| ENSG00000166183 | ASPG | K13278 | -2.5192127 | 3.124697212 | 10.462362 | 0.001218314 | 0.0095882 | 0.3979058 | 2.6876091 |
| ENSG00000184347 | SLIT3 | K06850 | -2.5192127 | 3.124697212 | 10.462334 | 0.001218332 | 0.0095882 | 0.448366 | 2.7633987 |
| ENSG00000187535 | IFT140 | K19672 | -2.5192127 | 3.124697212 | 10.462334 | 0.001218332 | 0.0095882 | 0.501368 | 2.9856361 |
| ENSG00000186073 | C15orf41 | - | -2.5192127 | 3.124697212 | 10.462321 | 0.001218341 | 0.0095882 | 0.466899 | 3.1533101 |
| ENSG00000100918 | REC8 | K13054 | -2.5192124 | 3.124697212 | 10.462174 | 0.001218438 | 0.0095882 | 0.4588665 | 3.1535649 |
| ENSG00000185697 | MYBL1 | K09421 | -2.5192124 | 3.124697212 | 10.46217 | 0.001218441 | 0.0095882 | 0.5691489 | 2.9853723 |
| ENSG00000170889 | RPS9 | K02997 | -2.5192123 | 3.124697212 | 10.462111 | 0.00121848 | 0.0095882 | 0.3865979 | 3.1752577 |
| ENSG00000170085 | SIMC1 | - | -2.5192123 | 3.124697212 | 10.462106 | 0.001218483 | 0.0095882 | 0.4927048 | 2.8585859 |
| ENSG00000169223 | LMAN2 | K10082 | -2.5192123 | 3.124697212 | 10.462101 | 0.001218486 | 0.0095882 | 0.4578652 | 3.0449438 |
| ENSG00000167645 | YIF1B | K20362 | -2.5192122 | 3.124697212 | 10.462096 | 0.001218489 | 0.0095882 | 0.3343949 | 3.0414013 |
| ENSG00000175785 | PRIMA1 | - | -2.5192122 | 3.124697212 | 10.462096 | 0.00121849 | 0.0095882 | 0.3267974 | 2.8366013 |
| ENSG00000188001 | TPRG1 | - | -2.5192121 | 3.124697212 | 10.462017 | 0.001218542 | 0.0095882 | 0.5054545 | 3.0654545 |
| ENSG00000185888 | PRSS38 | - | -2.5192121 | 3.124697212 | 10.462005 | 0.001218549 | 0.0095882 | 0.3558282 | 2.8742331 |
| ENSG00000173627 | APOBEC4 | K18773 | -2.519212 | 3.124697212 | 10.461977 | 0.001218568 | 0.0095882 | 0.4659401 | 3.0980926 |
| ENSG00000172831 | CES2 | K03927 | -2.5192119 | 3.124697212 | 10.461925 | 0.001218602 | 0.0095882 | 0.4285714 | 2.94061 |
| ENSG00000170946 | DNAJC24 | K17867 | -2.5192117 | 3.124697212 | 10.461802 | 0.001218684 | 0.0095882 | 0.6241611 | 3.0134228 |
| ENSG00000100433 | KCNK10 | K04920 | -2.5192116 | 3.124697212 | 10.461769 | 0.001218705 | 0.0095882 | 0.4640884 | 2.9226519 |
| ENSG00000171865 | RNASEH1 | K03469 | -2.5192115 | 3.124697212 | 10.461708 | 0.001218745 | 0.0095882 | 0.4335664 | 2.9090909 |
| ENSG00000188051 | TMEM221 | - | -2.5192115 | 3.124697212 | 10.4617 | 0.001218751 | 0.0095882 | 0.3024055 | 2.6082474 |
| ENSG00000171634 | BPTF | K11728 | -2.5192111 | 3.124697212 | 10.461492 | 0.001218888 | 0.0095882 | 0.5902823 | 2.7728168 |
| ENSG00000171773 | NXNL1 | - | -2.5192111 | 3.124697212 | 10.46149 | 0.001218889 | 0.0095882 | 0.5188679 | 2.9811321 |
| ENSG00000188266 | HYKK | K18201 | -2.5192109 | 3.124697212 | 10.461383 | 0.00121896 | 0.0095882 | 0.4879357 | 3.0589812 |
| ENSG00000099797 | TECR | K10258 | -2.5192109 | 3.124697212 | 10.461364 | 0.001218972 | 0.0095882 | 0.3571429 | 3.4902597 |
| ENSG00000189143 | CLDN4 | K06087 | -2.5192107 | 3.124697212 | 10.461253 | 0.001219045 | 0.0095882 | 0.3062201 | 2.7129187 |
| ENSG00000188747 | NOXA1 | K21432 | -2.5192105 | 3.124697212 | 10.46116 | 0.001219107 | 0.0095882 | 0.389234 | 2.679089 |
| ENSG00000188487 | INSC | - | -2.5192103 | 3.124697212 | 10.461066 | 0.001219169 | 0.0095882 | 0.462867 | 2.7115717 |
| ENSG00000088356 | PDRG1 | - | -2.5192101 | 3.124697212 | 10.460938 | 0.001219254 | 0.0095882 | 0.5413534 | 3.1353383 |
| ENSG00000088280 | ASAP3 | K12488 | -2.5192089 | 3.124697212 | 10.460328 | 0.001219656 | 0.0095882 | 0.5005537 | 2.7740864 |
| ENSG00000087269 | NOP14 | K14766 | -2.5192078 | 3.124697212 | 10.459718 | 0.001220059 | 0.0095882 | 0.5355893 | 2.9743291 |
| ENSG00000086506 | HBQ1 | K13827 | -2.5192066 | 3.124697212 | 10.459108 | 0.001220462 | 0.0095882 | 0.4014085 | 2.9084507 |
| ENSG00000086062 | B4GALT1 | K07966 | -2.5192055 | 3.124697212 | 10.458498 | 0.001220865 | 0.0095882 | 0.4170854 | 2.9170854 |
| ENSG00000083444 | PLOD1 | K00473 | -2.5192043 | 3.124697212 | 10.457887 | 0.001221269 | 0.0095882 | 0.4704264 | 3.1801926 |
| ENSG00000078725 | BRINP1 | - | -2.5192041 | 3.124697212 | 10.45774 | 0.001221366 | 0.0095882 | 0.4875164 | 3.1484888 |
| ENSG00000077616 | NAALAD2 | K01301 | -2.5192034 | 3.124697212 | 10.457363 | 0.001221615 | 0.0095882 | 0.4689189 | 3.1405405 |
| ENSG00000072954 | TMEM38A | - | -2.5192026 | 3.124697212 | 10.456987 | 0.001221864 | 0.0095882 | 0.3311037 | 3.1371237 |
| ENSG00000072571 | HMMR | K06267 | -2.5192025 | 3.124697212 | 10.456918 | 0.00122191 | 0.0095882 | 0.6468966 | 3.0427586 |
| ENSG00000066230 | SLC9A3 | K12040 | -2.5192024 | 3.124697212 | 10.456848 | 0.001221956 | 0.0095882 | 0.4196643 | 3.0671463 |
| ENSG00000065485 | PDIA5 | K09583 | -2.5192023 | 3.124697212 | 10.456778 | 0.001222002 | 0.0095882 | 0.4643545 | 3.1811175 |
| ENSG00000065457 | ADAT1 | K15440 | -2.5192021 | 3.124697212 | 10.456709 | 0.001222048 | 0.0095882 | 0.4322709 | 2.8486056 |
| ENSG00000064652 | SNX24 | K17941 | -2.519202 | 3.124697212 | 10.456639 | 0.001222094 | 0.0095882 | 0.4752475 | 3.3861386 |
| ENSG00000053372 | MRTO4 | K14815 | -2.5192018 | 3.124697212 | 10.456534 | 0.001222164 | 0.0095882 | 0.5481172 | 3.083682 |
| ENSG00000043462 | LCP2 | K07361 | -2.5192016 | 3.124697212 | 10.456428 | 0.001222234 | 0.0095882 | 0.5478424 | 2.9962477 |
| ENSG00000039537 | C6 | K03995 | -2.5192014 | 3.124697212 | 10.456322 | 0.001222304 | 0.0095882 | 0.5321199 | 2.8351178 |
| ENSG00000035115 | SH3YL1 | K20523 | -2.5192012 | 3.124697212 | 10.456216 | 0.001222374 | 0.0095882 | 0.4532164 | 2.8157895 |
| ENSG00000013588 | GPRC5A | K08468 | -2.5191999 | 3.124697212 | 10.455546 | 0.001222817 | 0.0095882 | 0.394958 | 3.2044818 |
| ENSG00000008710 | PKD1 | K04985 | -2.5191987 | 3.124697212 | 10.454876 | 0.001223261 | 0.0095882 | 0.407855 | 2.7738787 |
| ENSG00000007402 | CACNA2D2 | K04859 | -2.5191974 | 3.124697212 | 10.454205 | 0.001223705 | 0.0095882 | 0.4978317 | 3.0242845 |
| ENSG00000007341 | ST7L | - | -2.5191962 | 3.124697212 | 10.453535 | 0.00122415 | 0.0095882 | 0.4452174 | 3.0591304 |
| ENSG00000004809 | SLC22A16 | K08212 | -2.5191959 | 3.124697212 | 10.453402 | 0.001224238 | 0.0095882 | 0.3968804 | 3.1334489 |

**Table S5. Oxygen (O) content and carbon (C) content of up regulated proteins and down regulated proteins in glioma.**

|  | **Differential expressed proteins** | | |
| --- | --- | --- | --- |
|  | Number of up regulated proteins | Mean [C] | Mean [O] |
| Cerebral cortex | 1254 | 2.934 | 0.469 |
| Glioma | 226 | 2.916 | 0.481 |
| P value (Wilcoxon test） | _ | 0.125 | 0.02 |
| P value (Kolmogorov-Smirnov test) | _ | 0.335 | 0.0195 |

**Table S6. Pathways enriched by genes encoding up regulated and down regulated proteins.**

| **Type** | **Gene Set Name** | **k/K** | **p-value** | **FDR q-value** | **Genes in Gene Set** | **Genes in Overlap** |
| --- | --- | --- | --- | --- | --- | --- |
| Up regulated proteins | Cell cycle | 0.0938 | 1.13e-12 | 2.09e-10 | 128 | 12 |
|  | p53 signaling pathway | 0.0725 | 1.84e-5 | 1.37e-3 | 69 | 5 |
|  | DNA replication | 0.1111 | 2.40e-5 | 1.37e-3 | 36 | 4 |
|  | VEGF signaling pathway | 0.0658 | 2.95e-5 | 1.37e-3 | 76 | 5 |
|  | Oocyte meiosis | 0.0439 | 2.03e-4 | 7.56e-3 | 114 | 5 |
|  | MAPK signaling pathway | 0.0262 | 2.75-4 | 8.52e-3 | 267 | 7 |
|  | Peroxisome | 0.0513 | 4.98e-4 | 1.32e-2 | 78 | 4 |
|  | Progesterone-mediated oocyte maturation | 0.0465 | 7.20e-4 | 1.62e-2 | 86 | 4 |
|  | Apoptosis | 0.0455 | 7.85e-4 | 1.62e-2 | 88 | 4 |
|  | Pathways in cancer | 0.0213 | 9.29e-4 | 1.73e-2 | 328 | 7 |
|  | Arginine and proline metabolism | 0.0556 | 2.07e-3 | 3.51e-2 | 54 | 3 |
|  | Arachidonic acid metabolism | 0.0517 | 2.54e-3 | 3.94e-2 | 58 | 3 |
|  | Glycolysis / Gluconeogenesis | 0.0484 | 3.08e-3 | 4.40e-2 | 62 | 3 |
|  | alpha-Linolenic acid metabolism | 0.1053 | 3.50e-3 | 4.65e-2 | 19 | 2 |
| Down regulated proteins | Focal adhesion | 0.1393 | 4.27e-13 | 7.94e-11 | 201 | 28 |
|  | Tight junction | 0.1567 | 3.68e-11 | 3.43e-9 | 134 | 21 |
|  | Regulation of actin cytoskeleton | 0.1204 | 8.21e-11 | 5.09e-9 | 216 | 26 |
|  | Calcium signaling pathway | 0.118 | 7.55e-9 | 3.51e-7 | 178 | 21 |
|  | Leukocyte transendothelial migration | 0.1441 | 9.88e-9 | 3.64e-7 | 118 | 17 |
|  | Neuroactive ligand-receptor interaction | 0.0956 | 1.17e-8 | 3.64e-7 | 272 | 26 |
|  | ECM-receptor interaction | 0.1667 | 2.96e-8 | 7.81e-7 | 84 | 14 |
|  | MAPK signaling pathway | 0.0936 | 3.36e-8 | 7.81e-7 | 267 | 25 |
|  | Axon guidance | 0.1318 | 3.85e-8 | 7.95e-7 | 129 | 17 |
|  | Endocytosis | 0.1093 | 6.22e-8 | 1.16e-6 | 183 | 20 |
|  | Chemokine signaling pathway | 0.1053 | 1.16e-7 | 1.96e-6 | 190 | 20 |
|  | Cytokine-cytokine receptor interaction | 0.0899 | 1.35e-7 | 2.09e-6 | 267 | 24 |
|  | Long-term potentiation | 0.1571 | 1.64e-6 | 2.35e-5 | 70 | 11 |
|  | Alzheimer's disease | 0.1006 | 1.91e-6 | 2.48e-5 | 169 | 17 |
|  | Cell adhesion molecules (CAMs) | 0.1119 | 2.00e-6 | 2.48e-5 | 134 | 15 |
|  | Fc epsilon RI signaling pathway | 0.1392 | 5.55e-6 | 6.46e-5 | 79 | 11 |
|  | Epithelial cell signaling in Helicobacter pylori infection | 0.1471 | 8.97e-6 | 9.81e-5 | 68 | 10 |
|  | GnRH signaling pathway | 0.1188 | 1.12e-5 | 1.12e-4 | 101 | 12 |
|  | Hypertrophic cardiomyopathy (HCM) | 0.1294 | 1.14e-5 | 1.12e-4 | 85 | 11 |
|  | Pathways in cancer | 0.0701 | 1.63e-5 | 1.51e-4 | 328 | 23 |
|  | Dilated cardiomyopathy | 0.1196 | 2.44e-5 | 2.07e-4 | 92 | 11 |
|  | Arrhythmogenic right ventricular cardiomyopathy (ARVC) | 0.1316 | 2.45e-5 | 2.07e-4 | 76 | 10 |
|  | Vascular smooth muscle contraction | 0.1043 | 4.19e-5 | 3.39e-4 | 115 | 12 |
|  | SNARE interactions in vesicular transport | 0.1842 | 4.52e-5 | 3.51e-4 | 38 | 7 |
|  | Amyotrophic lateral sclerosis (ALS) | 0.1509 | 5.83e-5 | 4.34e-4 | 53 | 8 |
|  | Melanogenesis | 0.1078 | 6.41e-5 | 4.58e-4 | 102 | 11 |
|  | Complement and coagulation cascades | 0.1304 | 6.65e-5 | 4.58e-4 | 69 | 9 |
|  | Lysosome | 0.0992 | 6.92e-5 | 4.60e-4 | 121 | 12 |
|  | Long-term depression | 0.1286 | 7.46e-5 | 4.79e-4 | 70 | 9 |
|  | Huntington's disease | 0.0811 | 9.48e-5 | 5.87e-4 | 185 | 15 |
|  | Gap junction | 0.1111 | 1.06e-4 | 6.37e-4 | 90 | 10 |
|  | Phosphatidylinositol signaling system | 0.1184 | 1.43e-4 | 8.29e-4 | 76 | 9 |
|  | Wnt signaling pathway | 0.0861 | 1.50e-4 | 8.44e-4 | 151 | 13 |
|  | Natural killer cell mediated cytotoxicity | 0.0876 | 2.26e-4 | 1.24e-3 | 137 | 12 |
|  | Prion diseases | 0.1714 | 2.42e-4 | 1.28e-3 | 35 | 6 |
|  | Neurotrophin signaling pathway | 0.0873 | 4.20e-4 | 2.17e-3 | 126 | 11 |
|  | Inositol phosphate metabolism | 0.1296 | 4.45e-4 | 2.24e-3 | 54 | 7 |
|  | Leishmania infection | 0.1111 | 5.13e-4 | 2.51e-3 | 72 | 8 |
|  | Adherens junction | 0.1067 | 6.77e-4 | 3.15e-3 | 75 | 8 |
|  | B cell receptor signaling pathway | 0.1067 | 6.77e-4 | 3.15e-3 | 75 | 8 |
|  | Oxidative phosphorylation | 0.0815 | 7.51e-4 | 3.41e-3 | 135 | 11 |
|  | Insulin signaling pathway | 0.0803 | 8.48e-4 | 3.76e-3 | 137 | 11 |
|  | Cardiac muscle contraction | 0.1 | 1.04e-3 | 4.49e-3 | 80 | 8 |
|  | Toll-like receptor signaling pathway | 0.0882 | 1.27e-3 | 5.37e-3 | 102 | 9 |
|  | Glutathione metabolism | 0.12 | 1.71e-3 | 7.06e-3 | 50 | 6 |
|  | ErbB signaling pathway | 0.092 | 1.79e-3 | 7.24e-3 | 87 | 8 |
|  | Hematopoietic cell lineage | 0.0909 | 1.93e-3 | 7.62e-3 | 88 | 8 |
|  | Pancreatic cancer | 0.1 | 2.11e-3 | 8.19e-3 | 70 | 7 |
|  | Proximal tubule bicarbonate reclamation | 0.1739 | 2.59e-3 | 9.81e-3 | 23 | 4 |
|  | Viral myocarditis | 0.0959 | 2.69e-3 | 1.00e-2 | 73 | 7 |
|  | Vibrio cholerae infection | 0.1071 | 3.06e-3 | 1.12e-2 | 56 | 6 |
|  | VEGF signaling pathway | 0.0921 | 3.38e-3 | 1.21e-2 | 76 | 7 |
|  | Fc gamma R-mediated phagocytosis | 0.0825 | 3.54e-3 | 1.24e-2 | 97 | 8 |
|  | Glycosphingolipid biosynthesis - lacto and neolacto series | 0.1538 | 4.11e-3 | 1.41e-2 | 26 | 4 |
|  | NOD-like receptor signaling pathway | 0.0968 | 5.10e-3 | 1.72e-2 | 62 | 6 |
|  | Glyoxylate and dicarboxylate metabolism | 0.1875 | 7.35e-3 | 2.38e-2 | 16 | 3 |
|  | Adipocytokine signaling pathway | 0.0896 | 7.44e-3 | 2.38e-2 | 67 | 6 |
|  | Parkinson's disease | 0.0677 | 7.51e-3 | 2.38e-2 | 133 | 9 |
|  | Apoptosis | 0.0795 | 7.54e-3 | 2.38e-2 | 88 | 7 |
|  | Prostate cancer | 0.0787 | 8.01e-3 | 2.48e-2 | 89 | 7 |
|  | Alanine, aspartate and glutamate metabolism | 0.125 | 8.75e-3 | 2.63e-2 | 32 | 4 |
|  | Steroid biosynthesis | 0.1765 | 8.75e-3 | 2.63e-2 | 17 | 3 |

**Table S7. Functional dissection of up regulated proteins in glioma.**

| **Pathway** | **O** | **C** | **C:O** | **Protein name** | **Class** | **Description** |
| --- | --- | --- | --- | --- | --- | --- |
| MAPK signaling pathway | 0.68125 | 2.78125 | 4.082568807 | MAX | Other oncoprotein/transcription factor | A member of the basic helix-loop-helix leucine zipper (bHLHZ) family of transcription factors |
| Pathways in cancer | 0.68125 | 2.78125 | 4.082568807 | MAX | Other oncoprotein/transcription factor | A member of the basic helix-loop-helix leucine zipper (bHLHZ) family of transcription factors |
| Cell cycle | 0.564157706 | 3.035842294 | 5.38119441 | CCNB3 | Cyclins | The protein encoded by this gene belongs to the highly conserved cyclin family, whose members are characterized by a dramatic periodicity in protein abundance through the cell cycle. |
| P53 signaling pathway | 0.564157706 | 3.035842294 | 5.38119441 | CCNB3 | Cyclins | The protein encoded by this gene belongs to the highly conserved cyclin family, whose members are characterized by a dramatic periodicity in protein abundance through the cell cycle. |
| Progesterone-mediated oocyte | 0.564157706 | 3.035842294 | 5.38119441 | CCNB3 | Cyclins | The protein encoded by this gene belongs to the highly conserved cyclin family, whose members are characterized by a dramatic periodicity in protein abundance through the cell cycle. |
| Maturation | 0.560375147 | 2.899179367 | 5.173640163 | MCM3 | Cell cycle | The protein encoded by this gene is one of the highly conserved mini-chromosome maintenance proteins (MCM) that are involved in the initiation of eukaryotic genome replication. |
| DNA replication | 0.560375147 | 2.899179367 | 5.173640163 | MCM3 | Cell cycle | The protein encoded by this gene is one of the highly conserved mini-chromosome maintenance proteins (MCM) that are involved in the initiation of eukaryotic genome replication. |
| Cell cycle | 0.546894032 | 2.96589525 | 5.423162581 | MCM6 | Cell cycle | The protein encoded by this gene is one of the highly conserved mini-chromosome maintenance proteins (MCM) that are essential for the initiation of eukaryotic genome replication. |
| DNA replication | 0.546894032 | 2.96589525 | 5.423162581 | MCM6 | Cell cycle | The protein encoded by this gene is one of the highly conserved mini-chromosome maintenance proteins (MCM) that are essential for the initiation of eukaryotic genome replication. |
| P53 signaling pathway | 0.545126354 | 3.007220217 | 5.51655629 | CASP3 | Other oncoprotein/apoptosis | This gene encodes a protein that belongs to a highly conserved family of cysteinyl aspartate-specific proteases that function as essential regulators of programmed cell death through apoptosis. |
| MAPK signaling pathway | 0.545126354 | 3.007220217 | 5.51655629 | CASP3 | Other oncoprotein/apoptosis | This gene encodes a protein that belongs to a highly conserved family of cysteinyl aspartate-specific proteases that function as essential regulators of programmed cell death through apoptosis. |
| Apoptosis | 0.545126354 | 3.007220217 | 5.51655629 | CASP3 | Other oncoprotein/apoptosis | This gene encodes a protein that belongs to a highly conserved family of cysteinyl aspartate-specific proteases that function as essential regulators of programmed cell death through apoptosis. |
| Pathways in cancer | 0.545126354 | 3.007220217 | 5.51655629 | CASP3 | Other oncoprotein/apoptosis | This gene encodes a protein that belongs to a highly conserved family of cysteinyl aspartate-specific proteases that function as essential regulators of programmed cell death through apoptosis. |
| Cell cycle | 0.539823009 | 2.904867257 | 5.38114754 | MCM2 | Cyclins | The protein encoded by this gene belongs to the highly conserved cyclin family, whose members are characterized by a dramatic periodicity in protein abundance through the cell cycle. |
| DNA replication | 0.539823009 | 2.904867257 | 5.38114754 | MCM2 | Cyclins | The protein encoded by this gene belongs to the highly conserved cyclin family, whose members are characterized by a dramatic periodicity in protein abundance through the cell cycle. |
| VEGF signaling pathway | 0.535714286 | 3 | 5.599999997 | CHP2 | Other oncoprotein/signaling | This gene product is a small calcium-binding protein that regulates cell pH by controlling plasma membrane-type Na+/H+ exchange activity |
| Oocyte meiosis | 0.535714286 | 3 | 5.599999997 | CHP2 | Other oncoprotein/signaling | This gene product is a small calcium-binding protein that regulates cell pH by controlling plasma membrane-type Na+/H+ exchange activity |
| MAPK signaling pathway | 0.535714286 | 3 | 5.599999997 | CHP2 | Other oncoprotein/signaling | This gene product is a small calcium-binding protein that regulates cell pH by controlling plasma membrane-type Na+/H+ exchange activity |
| Apoptosis | 0.535714286 | 3 | 5.599999997 | CHP2 | Other oncoprotein/signaling | This gene product is a small calcium-binding protein that regulates cell pH by controlling plasma membrane-type Na+/H+ exchange activity |
| MAPK signaling pathway | 0.529032258 | 3.006451613 | 5.68292683 | FGF1 | Other oncoprotein/signaling | Fibroblast growth factor 1 |
| Pathways in cancer | 0.529032258 | 3.006451613 | 5.68292683 | FGF1 | Other oncoprotein/signaling | Fibroblast growth factor 1 |
| Cell cycle | 0.52189781 | 3.035279805 | 5.815850818 | ANAPC2 | Cell cycle | Anaphase promoting complex subunit 2 |
| Oocyte meiosis | 0.52189781 | 3.035279805 | 5.815850818 | ANAPC2 | Cell cycle | Anaphase promoting complex subunit 2 |
| Progesterone-mediated oocyte maturation | 0.52189781 | 3.035279805 | 5.815850818 | ANAPC2 | Cell cycle | Anaphase promoting complex subunit 2 |
| Arginine and proline metabolism | 0.519626168 | 3.059813084 | 5.888489211 | P4HA2 | Other oncoprotein/metabolism | Prolyl 4-hydroxylase subunit alpha 2 |
| Pathways in cancer | 0.510075567 | 3.070528967 | 6.019753083 | STAT5A | Other oncoprotein/signaling | Signal transducer and activator of transcription 5A [*Mus musculus* |
| Apoptosis | 0.507662835 | 3.007662835 | 5.924528304 | CASP10 | Other oncoprotein/apoptosis | This gene encodes a protein which is a member of the cysteine-aspartic acid protease (caspase) family. |
| Cell cycle | 0.507649513 | 2.945757997 | 5.802739728 | MCM7 | Cyclins | The protein encoded by this gene is one of the highly conserved mini-chromosome maintenance proteins (MCM) that are essential for the initiation of eukaryotic genome replication. |
| DNA replication | 0.507649513 | 2.945757997 | 5.802739728 | MCM7 | Cyclins | The protein encoded by this gene is one of the highly conserved mini-chromosome maintenance proteins (MCM) that are essential for the initiation of eukaryotic genome replication. |
| Peroxisome | 0.497584541 | 3.019323671 | 6.067961165 | IDH1 | Other oncoprotein/metabolism | Isocitrate dehydrogenase(NADP(+)) 1, cytosolic |
| Cell cycle | 0.485018727 | 3.024344569 | 6.23552123 | RBL1 | Cell cycle | RB transcriptional corepressor like 1 |
| Cell cycle | 0.482578397 | 2.897212544 | 6.003610112 | CDC7 | Cell cycle | This gene encodes a cell division cycle protein with kinase activity that is critical for the G1/S transition. |
| Glycolysis / Gluconeogenesis | 0.480825959 | 2.820058997 | 5.865030671 | FBP2 | Other oncoprotein/metabolism | Fructose-bisphosphatase 2 |
| Cell cycle | 0.478114478 | 3.205387205 | 6.704225353 | CDK1 | Cell cycle | Cyclin dependent kinase 1. The protein encoded by this gene is a member of the Ser/Thr protein kinase family. This protein is a catalytic subunit of the highly conserved protein kinase complex known as M-phase promoting factor (MPF), which is essential for G1/S and G2/M phase transitions of eukaryotic cell cycle. |
| P53 signaling pathway | 0.478114478 | 3.205387205 | 6.704225353 | CDK1 | Cell cycle | Cyclin dependent kinase 1. The protein encoded by this gene is a member of the Ser/Thr protein kinase family. This protein is a catalytic subunit of the highly conserved protein kinase complex known as M-phase promoting factor (MPF), which is essential for G1/S and G2/M phase transitions of eukaryotic cell cycle. |
| Oocyte meiosis | 0.478114478 | 3.205387205 | 6.704225353 | CDK1 | Cell cycle | Cyclin dependent kinase 1. The protein encoded by this gene is a member of the Ser/Thr protein kinase family. This protein is a catalytic subunit of the highly conserved protein kinase complex known as M-phase promoting factor (MPF), which is essential for G1/S and G2/M phase transitions of eukaryotic cell cycle. |
| Progesterone-mediated oocyte maturation | 0.478114478 | 3.205387205 | 6.704225353 | CDK1 | Cell cycle | Cyclin dependent kinase 1. The protein encoded by this gene is a member of the Ser/Thr protein kinase family. This protein is a catalytic subunit of the highly conserved protein kinase complex known as M-phase promoting factor (MPF), which is essential for G1/S and G2/M phase transitions of eukaryotic cell cycle. |
| MAPK signaling pathway | 0.475206612 | 2.844628099 | 5.986086951 | EGFR | Other oncoprotein/signaling | The protein encoded by this gene is a transmembrane glycoprotein that is a member of the protein kinase superfamily. This protein is a receptor for members of the epidermal growth factor family. |
| Pathways in cancer | 0.475206612 | 2.844628099 | 5.986086951 | EGFR | Other oncoprotein/signaling | The protein encoded by this gene is a transmembrane glycoprotein that is a member of the protein kinase superfamily. This protein is a receptor for members of the epidermal growth factor family. |
| VEGF signaling pathway | 0.466666667 | 2.846153846 | 6.098901094 | PLA2G12B | Other oncoprotein | The protein encoded by this gene belongs to the phospholipase A2 (PLA2) group of enzymes, which function in glycolipid hydrolysis with the release of free fatty acids and lysophospholipids. |
| MAPK signaling pathway | 0.466666667 | 2.846153846 | 6.098901094 | PLA2G12B | Other oncoprotein | The protein encoded by this gene belongs to the phospholipase A2 (PLA3) group of enzymes, which function in glycolipid hydrolysis with the release of free fatty acids and lysophospholipids. |
| Arachidonic acid metabolism | 0.466666667 | 2.846153846 | 6.098901094 | PLA2G12B | Other oncoprotein | The protein encoded by this gene belongs to the phospholipase A2 (PLA4) group of enzymes, which function in glycolipid hydrolysis with the release of free fatty acids and lysophospholipids. |
| Alpha-Linolenic acid metabolism | 0.466666667 | 2.846153846 | 6.098901094 | PLA2G12B | Other oncoprotein | The protein encoded by this gene belongs to the phospholipase A2 (PLA5) group of enzymes, which function in glycolipid hydrolysis with the release of free fatty acids and lysophospholipids. |
| VEGF signaling pathway | 0.463576159 | 3.170529801 | 6.839285713 | PTGS2 | Other oncoprotein | Prostaglandin-endoperoxide synthase (PTGS), also known as cyclooxygenase, is the key enzyme in prostaglandin biosynthesis, and acts both as a dioxygenase and as a peroxidase. |
| Pathways in cancer | 0.463576159 | 3.170529801 | 6.839285713 | PTGS2 | Other oncoprotein | Prostaglandin-endoperoxide synthase (PTGS), also known as cyclooxygenase, is the key enzyme in prostaglandin biosynthesis, and acts both as a dioxygenase and as a peroxidase. |
| Arachidonic acid metabolism | 0.463576159 | 3.170529801 | 6.839285713 | PTGS2 | Other oncoprotein | Prostaglandin-endoperoxide synthase (PTGS), also known as cyclooxygenase, is the key enzyme in prostaglandin biosynthesis, and acts both as a dioxygenase and as a peroxidase. |
| VEGF signaling pathway | 0.462354189 | 2.72640509 | 5.896788987 | NFATC1 | Regulators of apoptosis | Nuclear factor of activated T cells, cytoplasmic, calcineurin dependent 1 |
| Peroxisome | 0.458803122 | 3.071986123 | 6.695652178 | NOS2 | Other oncoprotein | Nitric oxide is a reactive free radical which acts as a biologic mediator in several processes, including neurotransmission and antimicrobial and antitumoral activities. |
| Pathways in cancer | 0.458803122 | 3.071986123 | 6.695652178 | NOS2 | Other oncoprotein | Nitric oxide is a reactive free radical which acts as a biologic mediator in several processes, including neurotransmission and antimicrobial and antitumoral activities. |
| Arginine and proline metabolism | 0.458803122 | 3.071986123 | 6.695652178 | NOS2 | Other oncoprotein | Nitric oxide is a reactive free radical which acts as a biologic mediator in several processes, including neurotransmission and antimicrobial and antitumoral activities. |
| Peroxisome | 0.45709571 | 3.140264026 | 6.870036094 | ABCD4 | Other oncoprotein | The protein encoded by this gene is a member of the superfamily of ATP-binding cassette (ABC) transporters. |
| Cell cycle | 0.452655889 | 2.972286374 | 6.566326532 | CCNB1 | Cyclins | The protein encoded by this gene is a regulatory protein involved in mitosis. |
| P53 signaling pathway | 0.452655889 | 2.972286374 | 6.566326532 | CCNB1 | Cyclins | The protein encoded by this gene is a regulatory protein involved in mitosis. |
| Oocyte meiosis | 0.452655889 | 2.972286374 | 6.566326532 | CCNB1 | Cyclins | The protein encoded by this gene is a regulatory protein involved in mitosis. |
| Progesterone-mediated oocyte maturation | 0.452655889 | 2.972286374 | 6.566326532 | CCNB1 | Cyclins | The protein encoded by this gene is a regulatory protein involved in mitosis. |
| Glycolysis / Gluconeogenesis | 0.446640316 | 3.031620553 | 6.787610622 | PGAM2 | Other oncoprotein | Phosphoglycerate mutase (PGAM) catalyzes the reversible reaction of 3-phosphoglycerate (3-PGA) to 2-phosphoglycerate (2-PGA) in the glycolytic pathway. |
| Cell cycle | 0.440881764 | 2.78757515 | 6.322727265 | CDC20 | Cell cycle | CDC20 appears to act as a regulatory protein interacting with several other proteins at multiple points in the cell cycle. |
| Oocyte meiosis | 0.440881764 | 2.78757515 | 6.322727265 | CDC20 | Cell cycle | CDC21 appears to act as a regulatory protein interacting with several other proteins at multiple points in the cell cycle. |
| Glycolysis / Gluconeogenesis | 0.440092166 | 2.801843318 | 6.366492145 | ENO3 | Regulators of apoptosis | This gene encodes one of the three enolase isoenzymes found in vertebrates. Enolase is a dimeric enzyme that converts 2-phosphoglycerate to phosphoenolpyruvate as part of the glycolytic pathway. |
| Apoptosis | 0.437194127 | 2.828711256 | 6.470149257 | AIFM1 | Cell cycle | This gene encodes a flavoprotein essential for nuclear disassembly in apoptotic cells, and it is found in the mitochondrial intermembrane space in healthy cells. |
| Peroxisome | 0.432664756 | 3.005730659 | 6.947019875 | ACSL1 | Other oncoprotein | The protein encoded by this gene is an isozyme of the long-chain fatty-acid-coenzyme A ligase family. |
| VEGF signaling pathway | 0.381944444 | 2.805555556 | 7.345454555 | PLA2G2A | Regulators of apoptosis | The protein encoded by this gene is a member of the phospholipase A2 family (PLA2). PLA2s constitute a diverse family of enzymes with respect to sequence, function, localization, and divalent cation requirements. |
| MAPK signaling pathway | 0.381944444 | 2.805555556 | 7.345454555 | PLA2G2A | Regulators of apoptosis | The protein encoded by this gene is a member of the phospholipase A2 family (PLA2). PLA3s constitute a diverse family of enzymes with respect to sequence, function, localization, and divalent cation requirements. |
| Arachidonic acid metabolism | 0.381944444 | 2.805555556 | 7.345454555 | PLA2G2A | Regulators of apoptosis | The protein encoded by this gene is a member of the phospholipase A2 family (PLA2). PLA4s constitute a diverse family of enzymes with respect to sequence, function, localization, and divalent cation requirements. |
| Alpha-Linolenic acid metabolism | 0.381944444 | 2.805555556 | 7.345454555 | PLA2G2A | Regulators of apoptosis | The protein encoded by this gene is a member of the phospholipase A2 family (PLA2). PLA5s constitute a diverse family of enzymes with respect to sequence, function, localization, and divalent cation requirements. |
| Cell cycle | 0.367088608 | 2.484177215 | 6.767241371 | CDKN1C | Cyclins | Cyclin dependent kinase inhibitor 1C |
| Arginine and proline metabolism | 0.346153846 | 2.534965035 | 7.323232327 | PYCRL | Other oncoprotein | Pyrroline-5-carboxylate reductase-like |
| P53 signaling pathway | 0.218390805 | 2.325670498 | 10.64912279 | BBC3 | Other oncoprotein | This gene encodes a member of the BCL-2 family of proteins. |
